# A comparative assessment of *in vitro* cytotoxic activity and phytochemical profiling of *Andrographis nallamalayana* J.L.Ellis and *Andrographis paniculata* (Burm. f.) Nees using UPLC-QTOF-MS/MS approach†

**DOI:** 10.1039/d1ra07496b

**Published:** 2021-11-08

**Authors:** Narender Goel, Rahul L. Gajbhiye, Moumita Saha, Chennuru Nagendra, Araveeti Madhusudhana Reddy, V. Ravichandiran, Krishna Das Saha, Parasuraman Jaisankar

**Affiliations:** Department of Natural Products, National Institute of Pharmaceutical Education and Research (NIPER-Kolkata) Chunilal Bhawan, 168, Maniktala Main Road 700054 Kolkata India; Cancer Biology and Inflammatory Disorder Division, CSIR-Indian Institute of Chemical Biology 4, Raja S. C. Mullick Road, Jadavpur Kolkata-700032 India; Department of Botany, Yogi Vemana University Vemanapuram Kadapa Andhra Pradesh 516005 India; Laboratory of Catalysis and Chemical Biology, Department of Organic and Medicinal Chemistry, CSIR-Indian Institute of Chemical Biology 4 Raja S. C. Mullick Road Kolkata-700032 India jaisankar@iicb.res.in +91-33-24995-790/774

## Abstract

*Andrographis paniculata* (Burm. f.) Nees and *Andrographis nallamalayana* J.L.Ellis have traditionally been used to treat various ailments such as mouth ulcers, intermittent fever, inflammation, snake bite. This study compares the comparative *in vitro* cytotoxic activity, and phytochemical profiling of methanol extract of *A. nallamalayana* (ANM) and *A. paniculata* (APM). UPLC-ESI-QTOF-MS/MS analysis has been performed. The cytotoxic activity of crude methanol extracts were evaluated against three different cancer cell lines (HCT 116, HepG2, and A549 cell line). Both plants' extract exhibited significant cytotoxic activity against tested cell lines in a dose-dependent manner. IC_50_ of ANM and APM in HCT 116 cell was 11.71 ± 2.48 μg ml^−1^ and 45.32 ± 0.86 μg ml^−1^ and in HepG2 cell line was 15.65 ± 2.25 μg ml^−1^ and 60.32 ± 1.05 μg ml^−1^ respectively. Cytotoxicity of these two extracts was comparatively similar in A549 cells. ANM induced cytotoxicity involved programmed cell death, externalisation of phosphatidylserine, ROS generation, up-regulation and down-regulation of major apoptotic markers. HRMS analysis of ANM and APM resulted in the identification of 59 and 42 compounds, respectively. Further, using the MS/MS fragmentation approach, 20 compounds, of which 18 compounds were identified for the first time from ANM, which belongs to phenolic acids, flavonoids, and their glycosides. Three known compounds, echioidinin, skullcapflavone I and 5,2′,6′-trihydroxy-7-methoxyflavone 2′-*O*-β-d-glucopyranoside, were isolated from *A. nallamalayana* and their crystal structures were reported for the first time. Subsequently, seven major compounds were identified in *A. nallamalayana* by direct comparison (retention time and UV-spectra) with authentic commercial standards and isolated compounds using HPLC-UV analysis. The cytotoxicity of phytochemicals from both the plants using *in silico* tools also justify their *in vitro* cytotoxic activity. It is the first report on the comparative characterisation of phytochemicals present in the methanolic extract of both the species of *Andrographis*, along with the cytotoxic activity of *A. nallamalayana*.

## Abstract 2

A comparative study of two Andrographis species have been done, and it was found that *Andrographis nallamalayana* J.L.Ellis is phytochemically and biologically different from *Andrographis paniculata* (Burm. f.) Nees.

## Graphical abstract

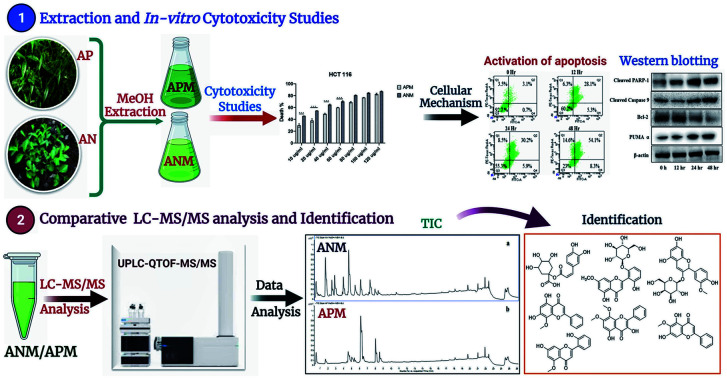

## 1 Introduction

Herbal medicines are the oldest kind of medicines the human race is aware of. Since time immemorial, plants have been used as sources for food, shelter and treating illness. Time and again, herbal drugs have been used by people all across the world. In India, they hold a special place. Due to the vast geographical difference, there is a drastic variety of medicinal plants, and they have been used for different ailments.^1^ With a large variety of plants, Acanthaceae is considered one of the top nine families of medicinal plants, including 2500 species and 250 genera.^2^ One of the important genera of Acanthaceae is *Andrographis*, which is widely used in the Indian medicine system.

Among several other members of the *Andrographis* genus, *Andrographis paniculata* (Burm. f.) Nees is an essential and well-studied medicinal plant. It has been used widely as a traditional medicine in India, China, Thailand and other Southeast Asian countries to treat various diseases such as wounds, malaria, jaundice, gonorrhoea, skin diseases, and boils.^3–6^ Various pharmacological studies have stated the importance of *A. paniculata* for treating diseases such as inflammation, influenza, diabetes, hypertension, ulcer.^7–10^ Various groups have also evaluated the *in vitro* and *in vivo* antitumor and immunomodulatory activity of *A. paniculata*.^11–14^ More than 55 *ent*-labdane diterpenoids, 30 flavonoids, eight quinic acids, four xanthones, and five rare noriridoids have been isolated from *A. paniculata*.^15–19^

*Andrographis nallamalayana* J.L.Ellis, commonly known as ‘Kachugadda’ is a lesser-known species of the *Andrographis* genus, an endemic procumbent herb distributed only in the core area of Nallamala Hills, Eastern Ghats, Andhra Pradesh, India. *A. nallamalayana* blended with *Acrocephalus indicus* and *Acrocephalus hispidus*, which is then boiled and further mixed with a lime pinch, the decoction is generally given orally for mouth ulcers by the tribals of Nallamala Hills, Eastern Ghats, Andhra Pradesh, India.^20,21^ Fresh root paste of *A. nallamalayana* mixed with leaves juice of *Becium filamentosum*, is also used in many regions as an antidote for snakebite.^21^ 5 g root of each *A. nallamalayana* and *A. indicus* ground, decoction prepared and given orally from the third day of delivery/menses for four days and also for the treatment of leucorrhoea by the Adivasi in the Eastern Ghats of Andhra Pradesh, India.^22^ Recent studies have shown that methanolic extract of *A. nallamalayana* used for antimicrobial,^23^ anti-psoriatic,^24^ anti-candidal^25^ and anti-proliferative, anti-inflammatory and pro-apoptotic activities.^26^ The preliminary phytochemical screening demonstrated the presence of flavonoids, alkaloids, phenols, steroids and triterpenoids. Parlapally *et al.* using GC/MS and LC/MS analysis, reported the presence of chromones, flavones/flavanones and their glycosides.^24^ UHPLC-ESI-QTOF-MS techniques have been used as a powerful analytical tool because of their high accuracy and sensitivity in characterising various complex natural products materials. The attained accurate mass spectra of elemental composition and tandem mass spectrometry (MS/MS) spectra allow detection and identification of the individual chemical structures.^27^ The novel drug development is a very complicated and time-consuming process. However, nearly 40% of the drug applicants failed due to unanticipated toxicity and adverse drug reactions. For the preliminary stage of drug development, computer-aided *in silico* strategies have become vital as they support more cost-effectively.^28–30^ For the development of bioactive phytoconstituents, the global research scenario recommends using virtual screening methods/technology.^31^ Prediction of possible pharmacological activity *via in silico* approach is based on the structure–activity relationship, which is usually correlated with the experimental data.^32,33^*In silico* studies combined with biological activities would reduce the time and cost for the development of novel drugs. *A. paniculata* is a mine for bitter compounds for medicinal purposes, but the scarcity of literature studies on *A. nallamalayana* related to phytochemical profiling and biological evaluation paves the way for this study. In this study, an attempt was made to determine the comparative *in vitro* cytotoxic activity and the phytochemical profiling of *A. nallamalayana* and *A. paniculata* and *in silico* prediction of cytotoxic activity of identified compounds in order to validate the ethnopharmacological use of these plants in India. To the best of our knowledge, it was the first report on the comparative characterisation of phytochemicals present in the methanolic extract of both the species of *Andrographis*, along with the cytotoxic activity of *A. nallamalayana*.

## 2 Materials and methods

### 2.1 Chemicals and reagents

Water, methanol and acetonitrile (LC-MS grade) were purchased from J. T. Baker (USA). MilliQ water (Millipore Elix 10 model, USA) was used for biological work. The additives, formic acid and acetic acid (LC-MS grade) were purchased from Sigma-Aldrich Co. (St. Louis, MO, USA). Hispidulin 7-glucoside (cat. no. SML2157), oroxylin A (cat. no. PHL82615), chlorogenic acid (cat. no. C3878), 4-di-*O*-caffeoylquinic acid (cat. no. SMB00224), quercetin, gallic acid standards and Folin–Ciocalteu reagents were purchased from Sigma-Aldrich Co. (St. Louis, MO, USA). Trypsin, Fetal Bovine Serum (FBS), ethylenediaminetetraacetic acid (EDTA), penicillin–streptomycin–neomycin (PSN) antibiotic, and Dulbecco's modified Eagle's medium (DMEM) were procured from Gibco BRL (Grand Island, NY, USA). 3-(4,5-Dimethylthiazol-2-yl)-2,5-diphenyltetrazolium bromide (45989, MTT-CAS 298-93-1-Calbiochem), dimethyl sulfoxide (DMSO), annexin-V/FITC/PI detection kits were obtained from Calbiochem, CA, USA. Plastic wares were procured from Genetix Biotech Asia Pvt. Ltd. HCT 116, Hep G2, A549, HEK 293 cell line was obtained from National Centre for Cell Science (NCCS), Pune, India.

### 2.2 Plant procurement and identification

#### 2.2.1 *Andrographis nallamalayana* J.L.Ellis

##### 2.2.1.1 Description

Procumbent herb with woody rootstock, 25–50 cm high; glabrous, very sparsely puberulous when young, black when dry. Leaves obovate or elliptic, glabrous. Flowers pedicellate, axillary and terminal racemes; pedicel 1.5 cm long. Capsule elliptic-oblong, sharply pointed, sparsely hairy. Seeds 4–6, brown, rugose.

##### 2.2.1.2 Specimen examined

Jyothi forest, Kadapa (YSR), Andhra Pradesh, India, 5110 CN & AMR, 02-09-2018. Coordinates: 15° 02 40.04′N, 78° 48 41.46′E, 356 m.

#### 2.2.2 *Andrographis paniculata* (Burm. f.) Nees

##### 2.2.2.1 Description

Perennial, erect or procumbent branched herb, 30 to 90 cm height; branches quadrangular, slightly winged. Leaves linear-obovate glabrous, apex acuminate. Panicle branches zigzag to 15 cm terminal; flowers unilateral, distant. Capsule oblong compressed minutely hairy; seeds 8–12, rugose.

##### 2.2.2.2 Specimen examined

Mallelathertham waterfalls, Mannur (NKL), Telangana, India, 5141 CN & AMR, 28-10-2018. Coordinates: 16° 15′ 961′′N 78°51′ 335′′E, 596 m.

The plants were adequately identified by taxonomist Dr A. Madhusudhana Reddy, Associate Professor, Dept. of Botany, Yogi Vemana University, Kadapa, Andhra Pradesh, India, in consultation with Herbarium of Botanical Survey of India (BSI) Deccan Regional Centre, Hyderabad. The above voucher numbers given to the herbarium sheets, and the herbarium sheets were deposited in the Herbarium, Department of Botany, Yogi Vemana University, Kadapa, Andhra Pradesh, India (ESI Fig. 1†).

### 2.3 Extraction and isolation

The leaves of *A. nallamalayana* (400 g) and *A. paniculata* (800 g) were shade dried for 7–8 days to achieve an optimum moisture content varied from 7% and 9%, respectively, before grinding to lesser particle size. The powdered leaves were defatted with petroleum ether (3 × 48 h) and then extracted with methanol (3 × 72 h) at room temperature using the cold maceration method. The methanol extracts of *A. nallamalayana* (53.38 g) and *A. paniculata* (87.25 g) were filtered through Whatman filter paper, and the filtrates were concentrated at 40 °C under reduced pressure. The extracts were stored at 4 °C in an airtight container until further use.

The crude methanolic extract (47.6 g) of *A. nallamalayana* was dissolved in a minimum amount of chloroform and adsorbed on silica gel. Air-dried slurry was chromatographed over silica gel (100–200 mesh). The column was eluted with chloroform/methanol in the order of increasing polarity. Eight fractions were collected based on the thin layer chromatography (TLC) profiles, Fraction 1 [FR-1, 100% CHCl_3_, 7.5 g, 15.75% w/w], Fraction 2 [FR-2, CHCl_3_ : MeOH (98 : 2), 3.2 g, 6.72% w/w], Fraction 3 [FR-3, CHCl_3_ : MeOH (95 : 5), 6.25 g, 13.13% w/w], Fraction 4 [FR-4, CHCl_3_ : MeOH (90 : 10), 3.2 g, 6.72% w/w], Fraction 5 [FR-5, CHCl_3_ : MeOH (85 : 15), 8.8 g, 18.48% w/w], Fraction 6 [FR-6, CHCl_3_ : MeOH (80 : 20), 6.21 g, 13.04% w/w], Fraction 7 [FR-7, CHCl_3_ : MeOH (75 : 25), 5.5 g, 11.55% w/w], Fraction 8 [FR-8, CHCl_3_ : MeOH (70 : 30), 6.3 g, 13.23% w/w]. Fraction 1 (FR-1, 7.5 g) was further chromatographically separated on a silica gel column (100–200 mesh) with chloroform/methanol in the order of increasing polarity to produce five subfractions (Subfraction 1-1-5) Among these five subfractions; subfraction two [Sub.FR-2, CHCl_3_ : MeOH (99 : 1)] yielded compound 1 (18.9 mg, 0.039% w/w) and subfraction three [Sub.FR-3, CHCl_3_ : MeOH (98 : 2)] yielded compound 2 (6.9 mg, 0.014% w/w). Similarly, further purification of fraction four through silica gel chromatography gave compound 3 (102.51 mg, 0.215% w/w). The structure elucidation of three compounds was carried out using various spectral techniques such as X-ray crystallography, ^1^H- and ^13^C-NMR, mass spectrometry, and comparison with literature.

### 2.4 Characterization and structural determination

Characterization and structural determination of three compounds isolated from methanolic extract of *A. nallamalayana* were established mainly based on single-crystal X-ray crystallography, 1D NMR and mass spectral studies. The single-crystal X-ray diffraction (XRD) data was collected on a Bruker D8 Venture system (Bruker, Billerica, Massachusetts, United States) with microfocus optics using CuKα (*λ* = 1.54178) radiation. The data for three compounds were analysed and processed using Bruker Apex III software suite,^34^ incorporated with multiple tools such as cell_now and RLATT to determine unit cell, SAINT-plus for data reduction SADABS for absorption correction. The structure solution was performed with SHELXT,^35^ and full matrix least-squares refinements were performed using the SHELXL^36^ suite of programs incorporated in either Apex III suite^34^ or Olex 2.0-1.3-alpha.^37^ A Bruker Avance-600 MHz superconducting FT-NMR spectrophotometer (Bruker, Billerica, Massachusetts, United States) with RT-TXI probe used to record ^1^H and ^13^C NMR spectra for the isolated compounds in DMSO-*d*_6_ and tetramethylsilane (TMS) as an internal standard. HRMS of compounds was obtained on Agilent 6545B Q-TOF LC/MS instrument (Agilent Technologies, Santa Clara, California, United States) in negative ionization mode.

### 2.5 Cell culture

HCT 116 (human colorectal carcinoma), HepG2 (hepatocellular carcinoma), A549 (human lung cancer), HEK 293 (human embryonic kidney cell) cells were grown in a humidified environment below 5% CO_2_ in DMEM media combined with 10% FBS and 1% antibiotic (PSN) at 37 °C. Cells were harvested with 0.5% trypsin and seeded at optimum density the day before the experiment was performed.

### 2.6 Cytotoxicity assay

Cytotoxicity of ANM and APM was determined by MTT [(3-(4,5-dimethythiazol-2-yl)-2,5-diphenyltetrazolium bromide)] assay.^38^ Cells were seeded into 96-well plates (1 × 10^6^ per well) and treated with different ANM and APM concentrations for 24 h before assessment using the MTT assay. Both the extracts were dissolved in 0.05% of DMSO to achieve extract concentrations of (10, 20, 40, 60, 80, 100 and 120 μg ml^−1^) and held in a humidified (5% CO_2_) atmosphere and kept in the incubator for 24 h at 37 °C. MTT (5 mg ml^−1^) was added after incubation, and the plates were additionally incubated for another 4 h. Using an ELISA reader, the absorbance of DMSO-soluble intracellular formazan salt was measured at 595 nm. This experiment was carried out in triplicate. The percentage of cell death was determined by calculating the percentage inhibition and IC_50_ value.

### 2.7 Determination of intracellular ROS generation (iROS)

Reactive oxygen species (ROS) generation was determined using the 2′,7′-dichlorofluorescein diacetate (H_2_DCFDA) dye which uses an increase in green fluorescence intensity to quantify the intracellular ROS generation with respect to untreated cells.^39^ The cells treated with the ANM (IC_50_) were incubated at 37 °C with 10 μM of H_2_DCFH-DA for 30 minutes following the flow cytometer determination (BD LSRFortessa, San Jose, CA, USA). The increase in DCF fluorescence directly redirects the ROS produced inside the cells, representing the mean DCF fluorescence intensity.

### 2.8 Detection of apoptosis by flow cytometry

Cell apoptosis is another critical parameter for the toxicity of materials. The determination of apoptosis and necrosis were analysed by flow cytometry using annexin-V–FITC/propidium iodide (PI) detection kit (Calbiochem, CA, USA).^40^ Briefly, in a six-well plate, HCT 116 cells were seeded and treated with ANM (IC_50_) time-dependently up to 48 h and were stained with annexin-V/FITC-PI as per the direction of the manufacturer (Calbiochem, Merck Millipore, Burlington, Massachusetts, USA). The percentage of live, apoptotic (early and late), and necrotic cells were quantified using a flow cytometer (BD LSR Fortessa, San Jose, CA, USA).

### 2.9 Western blotting

Total protein isolation from HCT 116 cells was performed using cell lysis buffer, which is supplemented by phosphatase and protease inhibitor cocktail; proteins have been quantified by BCA assay kit (Thermo Fisher Scientific).^41^ Using treated and non-treated cells, 40 μg of proteins were first isolated and then separated electrophoretically into SDS polyacrylamide gel (12%) which was later transferred to PVDF membrane (Immobilon-P, Millipore Company, Bedford, MA, USA) by using wet trans-blot system (Transblot: wet transfer cell; Bio-Rad Laboratories, Inc., Hercules, CA, USA). The membranes were blocked with BSA for 2 h and then incubated with primary antibodies anti-bcl2 (SC-7392), anti-cleaved PARP 1 (SC-56196), anti-PUMA-α (SC-37701), anti-cleaved-caspase-9 (SC-56076) and anti-β-actin (SC-47778) with 11.707 ± 2.482 μg ml^−1^, ANM (IC_50_) for 0, 12, 24, and 48 h. After thorough washing, secondary antibodies were conjugated by the membranes and incubated with HRP. By adding ECL substrates, immunoreactive bands were visualised. β-Actin was used as loading endogenous control.

### 2.10 *In vitro* wound healing assay

HCT 116 cells were seeded in 6-well plates and incubated at 37 °C overnight for 24 h. Using pipette tip thrice washed with PBS, a straight wound was rendered onto the confluence cell layer. The cells in serum-free DMEM medium were then treated with ANM (IC_50_). The wound repopulation gap width was measured and recorded at 0, 12, 24 and 48 h of incubation and was then compared to the original gap size at 0 h. The distance was calculated using the image processing system ImageJ, and the gap size was checked from the digital images at each point in time.^42^

### 2.11 Determination of total phenolic and flavonoid content

The Folin–Ciocalteu (F–C) colorimetric method was used to determine the total phenolic content described earlier.^43^ Different concentrations of gallic acid (25 to 1000 μg ml^−1^) have been prepared and used to generate the calibration curve using a linear fit (*y* = 0.048*x* + 0.063, *R*^2^ = 0.987). Total phenolic content was represented as gallic acid equivalent (GAE) in mg g^−1^ of dried extract weight (mg of gallic acid per g dry weight). All the samples were done in triplicates. The aluminium chloride colourimetric method described by Chia-chi Chang *et al.* was used to determine total flavonoid content.^44^ Various quercetin concentrations ranging from 0 to 500 μg ml^−1^ have been prepared and used to generate the calibration curve. The total content of flavonoid was calculated by using the calibration curve (*y* = 0.063*x* + 0.131, *R*^2^ = 0.970) and expressed in quercetin equivalents (QE) per gram dry extract weight. All the other determinations were carried out in triplicate.

### 2.12 UPLC-QTOF-MS and MS/MS analysis

Metabolite separation of *A. nallamalayana* and *A. paniculata* methanolic extract was performed on the Agilent 1290 Infinity LC system. 1.0 mg of dried extract was dissolved in 1 ml of LC-MS-grade methanol containing 0.1% formic acid (v/v) and filtered through a 0.2 μm PTFE membrane filter before the analysis was performed. The chromatographic separation was achieved on Agilent ZORBAX SB-C18 column (2.1 × 100 mm, 1.8 μm) as the stationary phase. A linear gradient of 0.1% (v/v) aqueous formic acid (A) and acetonitrile (B): 0–20.0 min, 10–40% B (v/v); 20.0–22.0 min, 40–100% B (v/v); 22.0–26.0 min, 100% B (v/v); 26.0–27.0 min, 100–10% B (v/v); 27.0–30.0 min, 10% B was used as mobile phase. Before the next injection, the column was reconditioned for 5 minutes. 0.5 ml min^−1^ flow rate with a 0.5 μl injected volume was used for analysis, the UPLC system assembled with a diode array detector (DAD) and an autosampler.

The Agilent 1290 Infinity LC device was coupled to Agilent 6545B Accurate-Mass Quadrupole Time-of-Flight (QTOF) for MS/MS study with Agilent Jet Stream Thermal Gradient Technology with electrospray ionisation (ESI) source. The analysis was performed in both positive and negative ionisation mode to obtain high-resolution mass spectra. The ESI parameters have been optimised as: the flow of drying gas (N_2_), 8 l min^−1^; temperature of drying gas, 150 °C. Other parameters were set as: fragmentor voltage, 150 V; skimmer voltage, 65 V; capillary voltage, 3500 V; nebuliser gas, 35 psig; nozzle voltage 1500 V. Fixed collision energies of 10, 20, 30, 40, 50 and 70 V were used for MS/MS analysis. The UPLC-QTOF data acquisition was performed using Agilent MassHunter Acquisition B.06.01 software (Agilent Technologies, Santa Clara, CA, USA). With Agilent MassHunter Qualitative Analysis B.07.00 (MassHunterQual, Agilent Technologies), the data were deconvoluted into individual chemical peaks using Molecular Feature Extractor (MFE). The prediction of molecular formula and accurate molecular mass for putative molecules were screened in databases such as METLIN, CAS and MassBank. Agilent Technologies has provided an accurate mass MS/MS Library (PCDL) for the METLIN Personal Compound Database. METLIN PCDL contains all compounds and additionally accurate mass Q-TOF-MS/MS library reference spectra.

### 2.13 HPLC-UV analysis of characterised compounds in crude methanolic extracts of *A. nallamalayana*

10 mg of vacuum-dried methanolic extract was dissolved in 1 ml of HPLC-grade methanol and filtered through a 0.2 μm PTFE membrane filter before analysis. Authentic commercial standards of chlorogenic acid, 3,4-di-*O*-caffeoylquinic acid, hispidulin 7-glucoside, oroxylin A and isolated compounds, *i.e.* compound 1, 2 and 3, were accurately weighed and dissolved in HPLC-grade methanol to achieve a concentration of 1 mg ml^−1^. The analysis was carried out in an HPLC system (Shimadzu, Kyoto, Japan) equipped with LC-20AD and LC-20AT prominence liquid chromatography pump, DGU-20A_3_ prominence degasser, CBM-20A prominence communications bus module, SPD-20A prominence UV/VIS detector. An aliquot of 20 μl was injected using SIL-20AC HT prominence autosampler. The separation was achieved on a Phenomenex reverse phase HPLC column (Luna^®^ RP C_18_ column 4.6 × 260 mm, 5*μ* particle size, column temperature; 25 °C), and elution was carried out using mobile phase consisted of water (A) and acetonitrile (B) with a gradient system, *i.e.*, 0–40 min, 0–70% B; 40–50 min, 70–100% B; 50–60 min, 100% B; 60–65 min, 0% B, flow 1 ml min^−1^. The eluate was monitored at 254 nm and 320 nm. Data analysis was performed by LC solution version 1.25 (Shimadzu, Kyoto, Japan).

### 2.14 Prediction of the *in silico* biological activity

#### 2.14.1 *In silico* prediction of anticancer activity using PASS

PASS (prediction of activity spectra for substances), a software program, was used to obtain the identified compounds biological activity spectrum, including the anticancer activity. PASS is a widely used web tool (http://www.pharmaexpert.ru/passonline) that contains more than 1 million biologically relevant compounds and can predict 7000 different pharmacological effects with 95% accuracy according to leave-one-out cross-validation (LOOCV) estimation. The program is based on the multilevel neighbourhoods of atoms (MNA). MNA descriptors were used to represent the chemical structure, and the prediction of activity is usually based on the structure–activity relationship (SAR) analysis of the training set according to the Bayesian algorithm.^45–47^ PASS represents the activity spectrum as “probable activity (Pa) or probable inactivity (Pi)”. The experimental value of Pa and Pi lies within the range of 0.000 to 1.000. When the value of Pa > Pi, *i.e.* if Pa > 0.7, then it represents that the compound would be experimentally active. A higher value of Pa reflects the significant biological effect experimentally and *vice versa*.^48,49^ The structure of all the identified phytoconstituents (20 compounds) were obtained from the PubChem database. The structures were submitted using the SMILES format and were subjected to the evaluation of the biological activity spectrum, including the anticancer activity.

#### 2.14.2 *In silico* prediction of cell line cytotoxicity with CLC-Pred tool

CLC-Pred (Cell-Line Cytotoxicity Predictor), a freely available web-service for cell-line cytotoxicity profile prediction tool, was used to predict the cytotoxicity of the identified compounds in various cell lines (http://way2drug.com/Cell-line/). This prediction of cytotoxicity is based on the PASS (prediction of activity spectra for substances) program, which uses structure–cell line toxicity relationship using special training sets with leave-one-out cross-validation procedure. The predicted cytotoxicity is represented by Pa and Pi values; if Pa > 0.5, the probability of cytotoxicity would be high (active), whereas Pi value represents inactivity.^28,50^ The structures were submitted in SMILES format and subjected for evaluation of cytotoxicity using the CLC-Pred tool.

### 2.15 Statistical analysis

Results were represented as mean ± SEM of the multiple data points. Statistical importance in the deference was calculated by the analysis of variance (ANOVA) and paired *T* test using GraphPad Prism (version 8.4.3) software where *p* < 0.05 was considered as significant.

## 3 Results and discussion

### 3.1 Assessment of *in vitro* cytotoxicity of crude methanolic extracts of *A. nallamalayana* and *A. paniculata*

**Fig. 1:**
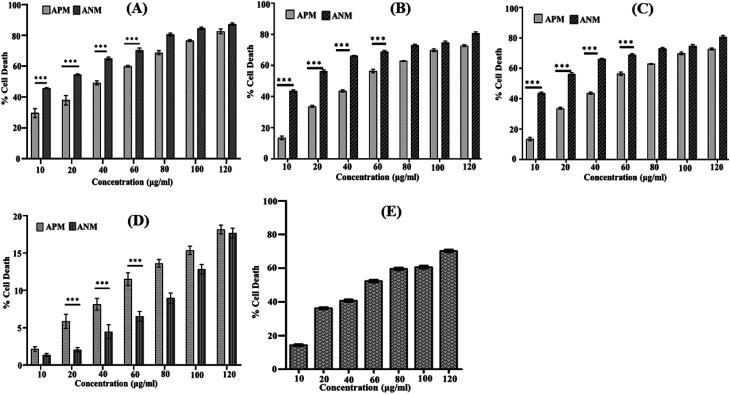
Cytotoxic effects of ANM and APM on different cancer cell lines: (A) HCT 116; (B) HepG2; (C) A549; (D) HEK 293 cells; (E) cytotoxicity of andrographolide (positive control) in HCT116 cells. All cells (1 × 10^6^ per well) were seeded in a 96-well plate and treated with different concentrations of ANM and APM extracts (10, 20, 40, 60, 80, 100, and 120 μg ml^−1^) for 24 h, and cytotoxicity was determined by MTT assay. Data are expressed as mean ± SEM for triplicate experiments. Here *** denotes *P* value < 0.0001; ** denotes *P* value < 0.001.

A comprehensive literature survey indicated only a few reports describe the cytotoxicity of *A. nallamalayana*,^26^ whereas previous studies showed that *A. paniculata* exhibited cytotoxic activities against several tested cancer cell lines.^51–54^ Motivated by these findings, we were also interested in investigating the role of ANM as an anti-proliferative agent. Our study revealed that methanolic extract of *A. nallamalayana* (ANM) and *A. paniculata* (APM) showed significant cytotoxicity towards three different types of cancer cell lines HepG2 (hepatocellular carcinoma), A549 (human lung cancer), HCT 116 (human colorectal carcinoma) in a dose-dependent manner as shown in Fig. 1A–C. Compared to APM, ANM effectively reduced cell viability in all tested cancer cell lines. The cytotoxicity of ANM was nearly four times higher than APM in HCT 116 and HepG2 cells. In HCT 116 cells, the IC_50_ of ANM and APM was 11.71 ± 2.48 μg ml^−1^ and 45.32 ± 0.86 μg ml^−1^, respectively, whereas, in HepG2 cells, it was 15.65 ± 2.25 μg ml^−1^ and 60.32 ± 1.05 μg ml^−1^, respectively. Cytotoxicity of these two extracts was comparatively similar in A549 cells (Table 1). Both extracts did not show significant cytotoxicity in HEK 293 cell line (human embryonic kidney cell) up to 120 μg ml^−1^ concentration (Fig. 1D). Andrographolide was used as the positive control, and the IC_50_ value of andrographolide (42.723 ± 0.668 μg ml^−1^) in HCT 116 cells was similar to the methanolic extract of *A. paniculata* (Fig. 1E). Our results are consistent with the prior studies, where an alcoholic extract of *A. paniculata* exhibited cytotoxic activity against HT-29 (human colon) and IMR-32 (human neuroblastoma) cancer cell lines resulted in 51.25 ± 0.85 and 50.25 ± 1.6% inhibition at 200 μg ml^−1^, respectively.^51^ In another study, methanolic extract of *A. paniculata* demonstrated significant anti-proliferative activity in MCF-7 (breast cancer) cell lines with minimum inhibition at a concentration of 31.25 μg ml^−1^.^52^ Dichloromethane fraction of methanol extract is also reported to maintain active compounds that contribute to the anticancer and immunostimulatory activity. The dichloromethane fraction significantly reduces the proliferation of HT 29 cells (colon cancer) and increases the proliferation of human peripheral blood lymphocytes (HPBLs) at low concentrations.^53^ Previously, the methanolic extract of *A. nallamalayana* reported for anti-proliferative activity against A375 and B16F10 melanoma cell lines.^26^ The cytotoxic activity of *A. nallamalayana* and *A. paniculata* was categorise according to the guidelines of National Cancer Institute (NCI) as follows: IC_50_ ≤ 20 μg ml^−1^ = highly active, IC_50_ 21–200 μg ml^−1^ = moderately active, IC_50_ 201–500 μg ml^−1^ = weakly active and IC_50_ > 501 μg ml^−1^ = inactive.^55,56^ Following the NCI guidelines, it was concluded that both the extracts showed moderate to high activity in cancer cell lines. Further experiments were focused on exploring the mechanism of cytotoxicity of the methanolic extract of *A. nallamalayana* as it showed the better cytotoxicity as compared to methanolic extract of *A. paniculata*.

**Table 1:** IC_50_ values of ANM and APM crude methanol extract in human cancer cell lines

Cell line	ANM (μg ml^−1^)	APM (μg ml^−1^)
HCT 116	11.717 ± 2.482	45.325 ± 0.859
HepG2	15.651 ± 2.258	60.325 ± 1.054
A549	81.868 ± 1.236	97.467 ± 1.496
HEK 293	>120	>120
Andrographolide	42.723 ± 0.668	—

### 3.2 Reactive oxygen species (ROS) generation by crude methanolic extract of *A. nallamalayana*

**Fig. 2:**
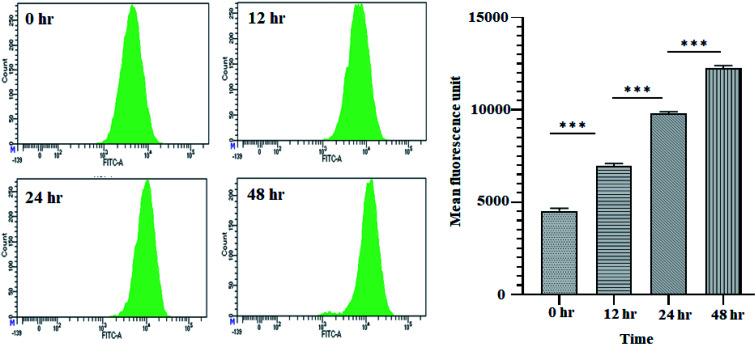
ANM induces reactive oxygen species (ROS) generation in HCT 116 cells. Cells treated with 11.707 ± 2.482 μg ml^−1^, ANM (IC_50_), for 0, 12, 24, and 48 h was studied for DCF fluorescence by flow cytometer. The left panel is for smooth histogram, and the right panel is the bar graph of mean fluorescence intensity. Data are expressed as mean ± SEM for triplicate experiments. Here *** denotes *P* value < 0.0001; ** denotes *P* value < 0.001.

It is well established that a rise in intracellular ROS (iROS) levels contribute to apoptosis-induced cell death, causing DNA damage and harm to other cell organelles.^57^ ROS production can be measured by 2′,7′-dichlorodihydrofluorescein diacetate (H_2_DCFDA), a non-fluorescent molecule. It was observed that following treatment with 11.707 ± 2.482 μg ml^−1^, ANM (IC_50_), the mean fluorescence intensity of dihydro-dichlorofluorescein (DCF) was increased significantly over time, indicating that ROS generation is directly related to ANM-induced cytotoxicity (Fig. 2). The relative DCF fluorescence intensity in ANM treated HCT16 cells increased in a time-dependent manner.

### 3.3 Annexin V–FITC/PI determination by flow cytometry of crude methanolic extract of *A. nallamalayana*

**Fig. 3:**
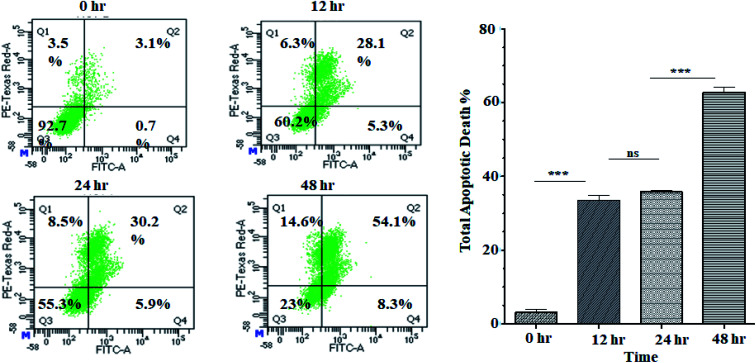
Flow cytometry analysis of annexin V–FITC/PI: HCT 116 cells were treated without or with ANM IC_50_ concentration value of 11.707 ± 2.482 μg ml^−1^ and incubated for 0, 12, 24, 48 h and analyzed by flow cytometer. The left column presents by the scattered plot of viable cells (Q3), percent dead cells at early apoptotic (Q4), late apoptotic (Q2), and necrotic phases (Q1). The right column presents a bar diagram of total apoptotic cells at different time points. Data are expressed as mean ± SEM for triplicate experiments. Here *** denotes *P* value < 0.0001; ** denotes *P* value < 0.001, and ns indicates non-significant.

Activation of apoptosis is an important strategy in the treatment of cancer. The externalisation of phosphatidylserine (PS) from the inner membrane to the cell's outer membrane is the main characteristic of early apoptosis, and late apoptosis is achieved through DNA fragmentation.^58^ To examine the possible induction of cell death (necrosis and/or apoptosis), experiment was performed using annexin V/propidium iodide assay by studying the exposed level of phosphatidylserine in the outer membrane of cells.^59^ In this assay, Q3, Q4, Q2 and Q1 reflect living cells, early apoptotic (EA), late apoptotic (LA), and necrotic, respectively. The percentage of apoptotic (early and late) cells were significantly increased in a time-dependent manner (5.3% EA/28.1% LA for 12 h, 5.9% EA/30.2% LA for 24 h and 8.3% EA/54.1% LA for 48 h) compared to control cells (0.7% EA and 3.1% LA) in ANM (IC_50_) treated cells (Fig. 3). A significant number of annexin-V–FITC positive cells indicated that ANM induced cytotoxicity in HCT 116 cells were triggered through apoptosis. The understanding of apoptosis can be used to develop new targeted medicines that stop cancer cells from growing and spreading.

### 3.4 Regulation of apoptosis markers by crude methanolic extract of *A. nallamalayana*

**Fig. 4:**
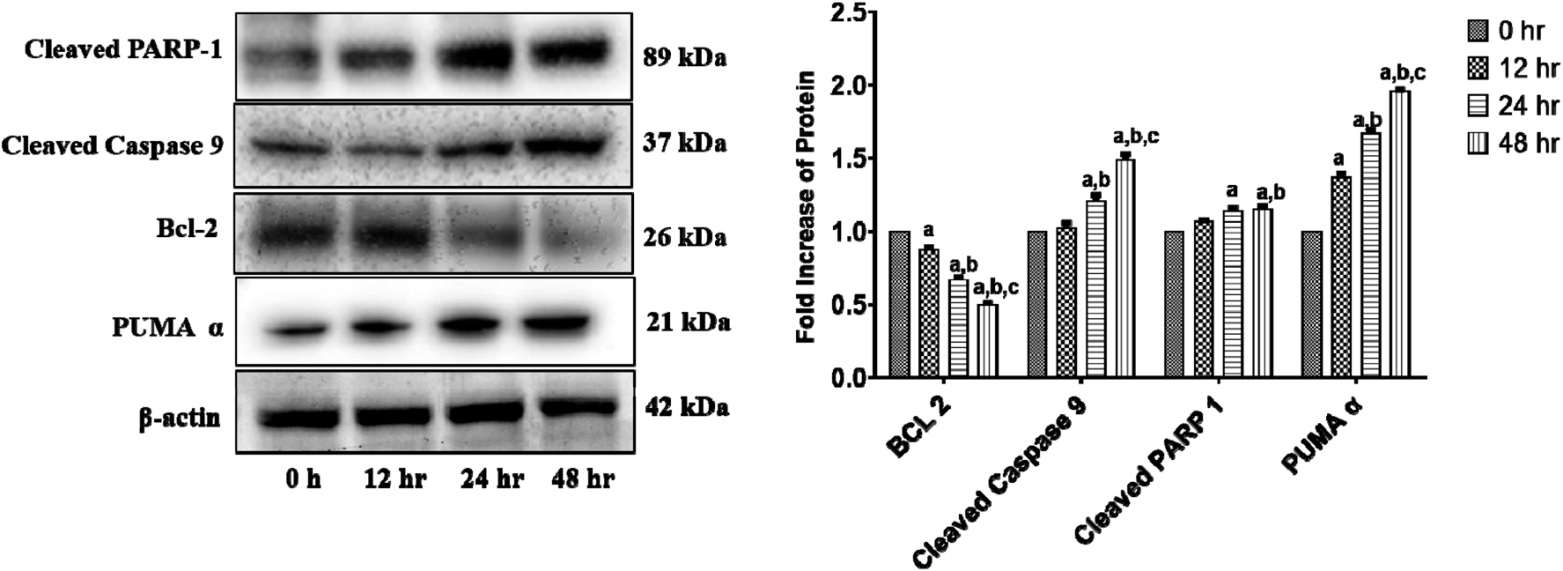
ANM alters the expression of intrinsic apoptosis pathway-related proteins in HCT 116 cells. Cells were treated with 11.707 ± 2.482 μg ml^−1^, ANM (IC_50_) for 0, 12, 24, 48 h. Expression levels of PUMA-α, Bcl-2, cleaved-caspase 9, and cleaved PARP-1 were detected by western blotting, and β-actin was used as the loading control. The blots were developed and captured by Azure Bio-system. The right column presents a bar diagram of densitometry data at different time points. Data are expressed as mean ± SEM for triplicate experiments. Here (a) *p* < 0.05 compared to 0 h, (b) *p* < 0.05 compared to 12 h, (c) *p* < 0.05 compared to 24 h [two-way ANOVA followed by Bonferroni *post hoc* test].

We investigated the levels of protein expressions related to the induction of apoptosis in HCT 116 cells after treatment with 11.707 ± 2.482 μg ml^−1^, ANM (IC_50_) for 0, 12, 24, and 48 h to investigate its effect on pro- and anti-apoptotic proteins. Previous studies in colon cancer cells showed that PUMA is a mitochondrial protein, and its mitochondrial position is necessary for apoptosis induction.^60^ PUMA (p53 up-regulated apoptosis modulator) a member of Bcl-2 homology 3 (BH3)-only subgroup of Bcl-2 family is one of the most effective killers. PUMA binds to Bcl-2 and Bcl-XL and induces a potential change of the mitochondrial membrane and activation of caspase.^61,62^ Bcl-2 suppresses mitochondrial apoptosis. The caspase family is at the apoptotic machinery centre, where all caspase enzyme plays a significant role in apoptosis execution. Cleavage of PARP-1 (poly[ADP-ribose]polymerase 1) promotes apoptosis by preventing DNA repair-induced survival and by blocking energy depletion-induced necrosis.^63^ PARP-1 cleavage produces an 89 kDa C-terminal fragment (containing the catalytic domain), and the 24 kDa N-terminal fragment with the DBD.^64^ It has been shown that the p24 fragment maintains its nucleolar localization, while p89 interacts with intact PARP-1 and blocks the PARP homodimerization, which is essential for enzyme activity.^65^ Western blot analysis showed that the main markers of apoptosis such as cleaved PARP1, PUMA-α, and cleaved-caspase 9 and Bcl-2 were up-regulated and down-regulated in ANM treated HCT cells (Fig. 4).

### 3.5 *In vitro* wound healing assay of crude methanolic extract of *A. nallamalayana*

**Fig. 5:**
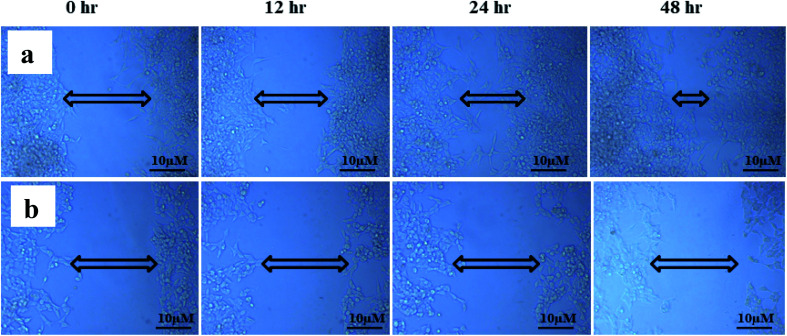
ANM's inhibitory effect on HCT 116 cell migration. (a) Control cells; (b) 11.707 ± 2.482 μg ml^−1^, ANM (IC_50_) treated cells. After treatment with ANM, the HCT 116 cell migration was decreased in a time-dependent manner (0, 12, 24, and 48 h). The result showed fewer or no cells in the denuded region.

The study of cell migration is of particular importance in cancer, as metastatic progression is the primary cause of death in cancer patients. Cancer can grow and spread all across the body only if cancer cells can migrate and invade *via* extracellular matrix (ECM) and intravasate into the bloodstream, binding to a distant site and eventually extravasate to form distant foci.^66,67^ The scratch wound assay was used to assess cell migration, a crucial step in forming metastatic foci. The scratch wound assay was carried out to detect ANM's inhibitory effect on HCT 116 cell migration. After treatment with ANM (IC_50_), the HCT 116 cell migration was decreased in a time-dependent manner (0, 12, 24 and 48 h). The result showed fewer or no cells in the denuded region, indicating that ANM could reduce site-specific cell migration (Fig. 5). Decreased migration in HCT 116 cells could be described as decreased metastatic potential. Over cell proliferation and migration are hallmarks of cancerous cells.^68^ The effectiveness of prospective cancer therapies is systematically estimated using *in vitro* cell-line proliferation screens. However, it is not clear whether tumour aggressiveness is more affected by the proliferative or migratory properties of cancer cells that make the therapy ineffective.^69^ Thus, inhibition of cell proliferation and migration is considered necessary in order to treat cancer effectively.^70^

### 3.6 Total phenolic and flavonoid content in crude methanolic extract of *A. nallamalayana* and *A. paniculata*

Phenolic and flavonoid compounds act as antioxidants due to their redox properties. Total phenolic content could be used as a basis for rapid antioxidant screening because of hydroxyl groups in phenolic compounds that facilitate free radical scavenging.^71^ Total phenolic content was determined using the Folin–Ciocalteu method in each extract. The results were derived from a standard calibration curve (*y* = 0.048*x* + 0.063, *R*^2^ = 0.987) of gallic acid (25 to 1000 μg ml^−1^) and expressed in gallic acid equivalents (GAE) per gram dry extract weight. Aluminium chloride colorimetric method was used to measure the flavonoids content in each methanolic extract. The results were derived from the calibration curve (*y* = 0.063*x* + 0.131, *R*^2^ = 0.970) of quercetin (0–500 μg ml^−1^) and expressed in quercetin equivalents (QE) per gram dry extract weight. The results were resumed in Table 2. The total phenolic content was found to be lower in ANM compared to APM while total flavonoid content was higher in ANM than APM. Phenolic and flavonoid are one of the most widely distributed secondary metabolites in the plant kingdom. Their anti-carcinogenic effects are primarily due to their ability to: (a) induce cell cycle arrest;^72,73^ (b) inhibit oncogenic signalling cascades controlling cell proliferation, angiogenesis, and apoptosis;^74–77^ (c) modulate ROS levels;^78–80^ (d) promote tumour suppressor proteins such as p53;^81,82^ and (e) halt cell migration.^83,84^ Multiple studies clearly suggest that the anticancer and apoptosis-inducing properties of polyphenolic compounds is mainly due to their prooxidant action rather than antioxidant activity.^85^ Flavonoids have a dual effect in terms of ROS homeostasis, acting as antioxidants under normal conditions and as powerful pro-oxidants in cancer cells, activating apoptotic pathways.^85,86^ Because of the presence of phenolic hydroxyl groups, flavonoids may directly scavenge ROS and chelate metal ions.^87,88^ The indirect antioxidant effects of flavonoids are associated with the activation of antioxidant enzymes, the repression of pro-oxidant enzymes, and stimulate the production of antioxidant enzyme and phase II detoxifying enzyme synthesis.^88^ The anticancer activity of flavonoid is mediated by both antioxidant and pro-oxidant activity.^89^ The high flavonoid and phenolic content could be responsible for the cytotoxicity of these crude extracts.

**Table 2:** Total phenolic and flavonoids content of methanolic extract of *A. nallamalayana* and *A. paniculata* aerial parts

Plant	Total phenolic content (mg GAE per g)	Total flavonoid content (mg QE per g dry mass (d.m.))
ANM	357.17 ± 1.29	474.98 ± 0.63
APM	408.60 ± 0.58	327.58 ± 0.90

### 3.7 Metabolite profiling by UPLC-QTOF-MS (HRMS) analysis of methanolic extract of *A. nallamalayana* and *A. paniculata* aerial parts

Since the phytochemical analysis showed that the extracts were rich in phenolic and flavonoid contents, they were used to identify and characterise metabolites using UPLC-QTOF-MS analysis. Accurate mass values (*m*/*z*) of all the primary ions identified in UPLC-MS analysis were screened against databases such as Metlin, MassBank and HMDB and literature within five ppm accuracy. Peak identification was carried out by matching retention times (Rt) and mass spectra with literature data and databases. The comparative phytochemical investigation revealed that both species have different chemical constituents. In UPLC-ESI-QTOF-MS analysis, 42 compounds were identified with andrographolides as the major constituents of *A. paniculata*, whereas a total of 59 compounds were identified from the methanolic extract of *A. nallamalayana*. Most of the compounds were identified for the first time from this species. Among all the identified compounds from both species, eight compounds were similar, *i.e.* chlorogenic acid, andrographidine B, 1,3-dicaffeoylquinic acid, apigenin 7-*O*-β-glucuronide, andrographidine D, andropaniculoside A, skullcapflavone I, oroxylin A (ESI Fig. 2†). The phytochemicals characterisation revealed that the identified compounds belong to phenolic acids, diterpenoids, flavonoids, and their glycosides. The names of the identified compounds, molecular formula, experimental mass (*m*/*z*), peak height, the retention time (min), score and adduct/ion species are summarised in ESI Tables S1 and S2.†

### 3.8 UPLC-QTOF-MS/MS analysis of methanolic extract of *A. nallamalayana* aerial parts

**Fig. 6:**
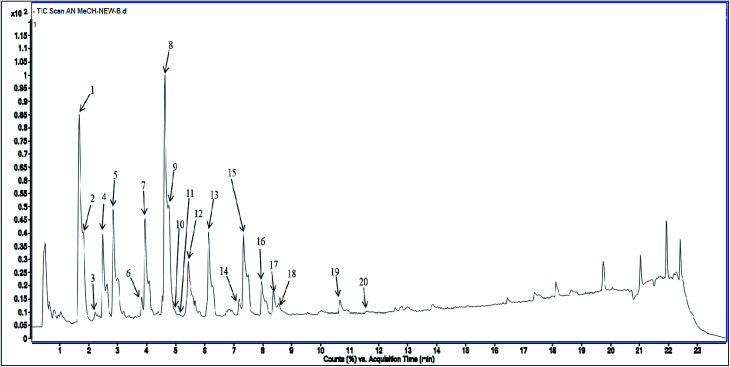
Total Ion Chromatogram (TIC) of methanol extract of *Andrographis nallamalayana* in the negative ionization mode.

**Fig. 7:**
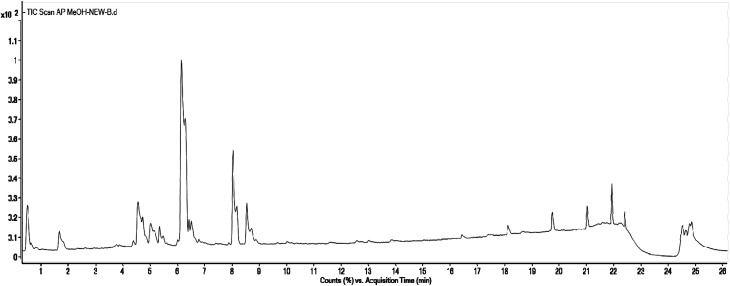
Total Ion Chromatogram (TIC) of methanol extract of *Andrographis paniculata* in the negative ionization mode.

Methanolic extract of *A. nallamalayana* aerial parts was analysed by UPLC-QTOF-MS/MS analysis using positive and negative ionisation mode. Usually, flavonoids in the negative mode exhibit better sensitivity and a cleaner mass spectral background than the positive mode. In the case of positive mode when there is no collision energy applied, most flavonoids usually gave [M + H]^+^, [M + Na]^+^ as molecular adduct ions along with different fragment ions in the complete scan mode, whereas most flavonoids predominantly yielded [M–H]^−^ ions in the negative mode.^90^ Therefore, the negative ion mode detection was selected for the analysis. A bunch of peaks were eluted with high relative abundance in the range of 1.5–15 minutes. All the compounds were characterised by their retention time, accurate mass, fragmentation patterns and UV spectra and by comparing it with the literature data. The total ion chromatograms (TIC) of the ANM and APM in the LC-MS/MS analysis are shown in Fig. 6 and 7. According to their elution order, compounds were numbered, keeping the same numbering of peaks. Four caffeoylquinic acids, one anthocyanidin-3-*O*-glycoside and 15 flavones/flavanol and their glycosides were characterised. For each identified compound, the UPLC-QTOF-MS/MS data were resumed in Table 3.

**Table 3:** Compounds identified by UPLC-QTOF-MS/MS analysis in methanol extracts of *A. nallamalayana*

Peak no.	RT (min)	HPLC-DAD *λ*_max_ (nm)	[M–H]^−^ (*m*/*z*)	Delta ppm	Fragments ions (*m*/*z*)	Accurate mass	Proposed molecular formula	Identification	References
1	1.66	217, 240sh, 324	353.090	−7.6	191, 179, 135, 173	354.095	C_16_H_18_O_9_	Chlorogenic acid	92
2	1.81	233, 305sh, 328	353.090	−7.6	191, 179, 173, 161	354.095	C_16_H_18_O_9_	1-*O*-Caffeoylquinic acid	93
3	2.21	255, 267sh, 352	477.105	−4.1	314, 299	478.111	C_22_H_22_O_12_	Isorhamnetin 3-glucoside	103
4	2.48	243, 303sh, 325	515.122	−5.2	353, 179, 191, 135	516.127	C_25_H_24_O_12_	3,4-Di-*O*-caffeoylquinic acid	92 and 94
5	2.83	226, 334	461.111	−5.3	299, 341, 285	462.116	C_22_H_22_O_11_	Hispidulin 7-glucoside (homoplantaginin)	104
6	3.82	256, 266sh, 348	447.095	−5.2	285, 175, 151, 133	448.101	C_21_H_20_O_11_	Luteolin 4′-glucoside	105
7	3.94	256, 271, 349	567.118	−4.6	285, 151, 101	568.122	C_28_H_24_O_13_	Neobignonoside	106 and 107
8	4.62	275, 327	461.112	−5.8	323, 299, 165, 284, 118	462.443	C_22_H_22_O_11_	5,2′,6′-Trihydroxy-7-methoxyflavone 2′-*O*-β-d-glucopyranoside	108
9	4.77	240, 260sh, 344	461.112	−6.6	341, 299, 165, 133, 137	462.116	C_22_H_22_O_11_	Luteolin 7-methyl ether 5-β-d-glucoside	109
10	4.94	229, 287, 309	337.095	−6.6	191, 173, 163, 119	338.100	C_16_H_18_O_8_	3-*p*-Coumaroyl quinic acid	95
11	5.07	277, 333	461.112	−6.4	299, 165, 161, 341	462.166	C_22_H_22_O_11_	Scutellarein 7-methyl ether 6-galactoside	110
12	5.43	258, 294, 332	581.133	−3.8	299, 165, 133	582.137	C_29_H_26_0_13_	2′′-*O*-Vanilloylvitexin	96
13	6.13	272, 307	429.085	−5.8	253, 175, 113	430.090	C_21_H_18_O_10_	Chrysin 7-glucuronide	111
14	7.18	279, 320	461.112	−3.6	299	461.110	C_22_H_22_O_11_	Peonidin 3-*O*-galactoside	112
15	7.33	265, 335	283.063	−7.4	268, 240, 165, 118	284.068	C_16_H_12_O_5_	Echioidinin	97
16	7.96	273, 321	283.063	−7.1	268, 240, 239, 211, 196, 165	284.068	C_16_H_12_O_5_	Oroxylin A	98 and 99
17	8.36	245, 277, 316	283.063	−7.4	268, 240, 211, 196, 165	284.068	C_16_H_12_O_5_	Wogonin	98 and 99
18	8.56	250sh, 271, 372	313.080	−4.3	283, 298	314.079	C_17_H_14_O_6_	3,5-Dihydroxy-7,8-dimethoxyflavone	100
19	10.65	270, 320	313.080	−4.9	298, 283, 255	314.079	C_17_H_14_O_6_	Skullcapflavone I	101
20	11.11	261, 276sh, 333	283.063	−5.3	268, 240, 239, 165, 121, 117	284.068	C_16_H_12_O_5_	7,2′-Dihydroxy-5-methoxyflavone	102

#### 3.8.1 Identification of phenolic acids

In the methanolic extract of *A. nallamalayana*, four caffeoylquinic acids were identified, but their correct identification is a rather tricky job due to widespread isomerism (geometrical and regional). In quinic acid, the linkage position of caffeoyl groups for monoacyl caffeoylquinic acids could be identified based on their characteristic fragmentation pathways. Diagnostic fragmentation ions (DFIs), *e.g. m*/*z* 173, 179 and 191 corresponds to [quinic acid–H–H_2_O], [caffeic acid–H]^−^ and [quinic acid–H]^−^ respectively, were suggested or calculated from the analysis of fragmentation pattern for each chemical class of chlorogenic acids (CGAs).^91^

Compound 1 (peak 1) displayed a deprotonated molecular ion peak at *m*/*z* 353.09 [M–H]^−^. In mass fragmentation, the base peak at *m*/*z* 191.0556 was obtained from the moiety of the quinic acid, [quinic acid–H]^−^. Additionally, the secondary peak at *m*/*z* 179.0344 (C_9_H_7_O_4_) was derived from the moiety of caffeic acid, [caffeic acid–H]^−^, together with a daughter ion at *m*/*z* 161.025. Compound 1, based on the fragmentation patterns and pseudo molecular ion at *m*/*z* 353 in the MS/MS experiment, was tentatively characterised and identified as chlorogenic acid.^92^ No distinct difference was observed in the MS/MS spectra of compound 1 and compound 2, but a secondary peak at *m*/*z* 135.0448 (C_8_H_7_O_2_), [caffeic acid–H–CO_2_]^−^ displayed by compound 1, which was absent in the spectra of compound 2. Based on the fragmentation pattern and previous literature reports, compound 2 (peak 2) was tentatively identified as 1-*O*-caffeoylquinic acid.^93^ Compounds 4 (peak 4) showed the [M–H]^−^ at *m*/*z* 515 (C_25_H_24_O_12_). The data analysed by mass suggested that this compound might be di-caffeoylquinic acids (DCQAs). The deprotonated molecular ions in the mass fragmentation spectra yielded characteristic fragments at *m*/*z* 353 [caffeoylquinic acid–H]^−^, 191 [quinic acid–H]^−^, 179 [caffeoyl–H]^−^, 173 [quinic acid–H–H_2_O]^−^ and 135 [caffeoyl–H–COO]^−^, which are specific to caffeoylquinic acids. The three isomeric compounds could be distinguished based on the relative intensity of molecular ion at *m*/*z* 335 [CQA–H_2_O–H]^−^. The base peak intensity at *m*/*z* 335 was higher in 3,4-DCQA (26% of base peak), barely detectable in 4,5-DCQA (2% of base peak). Fragment ion at *m*/*z* 173 in 3,4-DCQA mass spectrum was indicative of acylation at 4-position.^92,94^ Finally, compound 4 was provisionally identified as 3,4-di-*O*-caffeoylquinic acid. Compound 10 (peak 10) had the molecular ion at *m*/*z* 337 [M–H]^−^ and another secondary peak at *m*/*z* 191, 173, 163, 119 corresponds to [quinic acid–H]^−^, [quinic acid–H–H_2_O]^−^, [*p*-coumaric acid–H]^−^ and [p-coumaric acid–H–CO_2_]^−^ respectively. The ion at *m*/*z* 173 [QA–H–H_2_O]^−^ indicated that 4-OH of QA was substituted. Both compounds mass fragmentation spectra showed that ion at *m*/*z* 191 [QA–H]^−^ was indispensable. According to the fragmentation pattern and literature data compound, 10 was tentatively identified as 3-*p*-coumaroylquinic acid.^95^

#### 3.8.2 Identification of flavones

Compound 12 (peak 12) displayed a deprotonated molecular ion peak at *m*/*z* 581.1331 [M–H]^−^. The fragmentation pattern suggests that this compound may be a combination of orientin and vitexin derivatives because the retention time and molecular masses were identical. The fragment ions produced were found to be identical to those of the type II flavone C-glycosides. Compounds 12 were tentatively classified as 2′′-*O*-vanilloylvitexin based on earlier literature studies.^96^ Compound 15 (peak 15) exhibited deprotonated molecular ion at *m*/*z* 283 [M–H]^−^. The fragment ions at *m*/*z* 165.020 and 117.035 produced due to the loss of the C ring of flavone moiety along with a secondary peak at *m*/*z* 267 [M–OH]^−^. Compound 15 generated a radical anion at *m*/*z* 268 [M–H–CH_3_]˙. The loss of a 
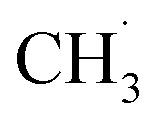
 radical from the deprotonated ion indicates the occurrence of the methoxy group. Based on the fragmentation behaviour and earlier literature data, compound 15 was characterised/identified as echioidinin.^97^ Compound 16 & 17 (peak 16 and 17) had the same *m*/*z* at 283 [M–H]^−^. The electrospray ionisation of compound 16 & 17 produced similar fragments ion as compound 16. Six ions were observed in MS/MS spectra for both compounds at *m*/*z* 268.03 [M–H–CH_3_]^−^˙, 240.04 [M–H–CO]^−^, 223.04 [M–H–CO_2_H]^−^˙, 211.04 [M–H–CO]^−^, 196.05, 165.02 under negative ionisation conditions. Although the fragment ions of compound 16 and 17 were identical, their MS/MS spectra could easily distinguish them. The relative abundance of the fragment ions at *m*/*z* 165 in compound 17 was higher than the fragment ion at *m*/*z* 211. On the contrary, it was the opposite in the case of compound 16. According to the fragmentation pattern, these isomers were tentatively identified as oroxylin A and wogonin.^98,99^ Compound 18 (peak 18) and compound 19 (peak 19) showed a deprotonated molecular ion at *m*/*z* 313 [M–H]^−^. By analysing their MS/MS spectra, it was concluded that both of them contain two –OCH_3_ groups since the ions of *m*/*z* 298 and 283 was observed, which indicates the presence of dimethoxylated flavanone. The fragment ion at *m*/*z* 255 [M–H–2CH_3_–CO]^−^ indicated a loss of CO from the parent ion. However, they were significantly different. The intensity of ion at *m*/*z* 298 was very weak in compound 18, whereas compound 19 showed the strong intensity of ion at *m*/*z* 298. Based on these data and earlier literature reports, compound 18 and 19 were putatively identified as 3,5-dihydroxy-7,8-dimethoxyflavone,^100^ and skullcapflavone I^101^ respectively. Compounds 20 (peak 20) exhibited [M–H]^−^ ion at *m*/*z* 283. A stable radical ion was formed at *m*/*z* 268 correspondings to [M–H–CH_3_]^−^. Compound 20 also showed minor daughter ions at *m*/*z* 240 and 239 due to CH_3_^−^ and CO or HCO loss, respectively. The peak at *m*/*z* 117 corresponds to the generation of the B-ring fragments. Fragment ions formed by A-ring at *m*/*z* 165 and *m*/*z* 121 indicate that methoxyl substituent occurs at the 8^th^ position. Finally, compound 20 was tentatively identified as 7,2′-dihydroxy-5-methoxyflavone.^102^

#### 3.8.3 Identification of flavonoid glycoside

Compound 3 (peak 3) displayed a pseudomolecular ion at *m*/*z* 477.1058 [M–H]^−^ and a secondary fragment ion at *m*/*z* 315 [M–162–H]^−^ was formed due to the loss of a hexose moiety. The fragmentation of the pseudo molecular ion also leads to a characteristic fragment ion corresponding to the aglycone moiety at *m*/*z* 315, designated to isorhamnetin. The fragment ion at *m*/*z* 299 indicates the loss of methyl group from the aglycone part, which confirmed the identification of isorhamnetin 3-*O*-glucoside based on fragmentation pattern, UV spectra and retention time in the extract of *A. nallamalayana*.^103^ Compound 5 (peak 5) with *m*/*z* at 461 [M–H]^−^ displayed fragment ion at *m*/*z* 299 due to the loss of a hexose moiety and secondary peaks at *m*/*z* 341, 285. Compound 5 was tentatively identified as hispidulin 7-glucoside (homoplantaginin).^104^ Similarly, Compound 6 (peak 6) displayed a deprotonated molecular ion at *m*/*z* 447 [M–H]^−^. The mass spectrum showed the high abundance of fragments at *m*/*z* 285 [M–H–162]^−^ is due to the loss of a hexose unit. The deprotonated aglycone fragment at *m*/*z* 285 suggested that it was originated from luteolin or kaempferol. Characteristic fragments at *m*/*z* 175, 151 and 133 confirmed luteolin as aglycone. Thus compound 6 was tentatively designated as luteolin 4′-glucoside.^105^ The molecular ion peak at *m*/*z* 567 [M–H]^−^ along with the characteristic fragment ion at *m*/*z* 285 [luteolin–H]^−^ supports the previously proposed structure. Compound 7 (peak 7) was, thus, tentatively identified as neobignonoside [luteolin-7-*O*-(6′′-*p*-hydroxybenzoyl-β-d-glucopyranoside)] from its negative ESI-MS/MS analysis.^106,107^ Compound 8 (peak 8) had the molecular ion at *m*/*z* 461 [M–H]^−^. Dissociation of the parent molecular ion at *m*/*z* 461 gave the subsequent fragment ion at *m*/*z* 299 [M–H–162]^−^ due to the loss of a sugar moiety and secondary peaks at *m*/*z* 165, 161, 341 in agreement with the previous report.^108^ Hence compound 8 was identified as 5,2′,6′-trihydroxy-7-methoxyflavone 2′-*O*-β-d-glucopyranoside. Compound 9 (peak 9) (*λ*_max_ 196-256-300) was tentatively identified as luteolin 7-methyl ether 5-β-d-glucoside which displayed [M–H]^−^ at *m*/*z* 461 and mass fragment ion at *m*/*z* 299 [aglycone–H]^−^, and secondary peaks at *m*/*z* 165, 133.^109^ Compound 11 (peak 11) based on its deprotonated molecular ion at *m*/*z* 461 [M–H]^−^ and fragments at *m*/*z* 299 [M–H–Hexose]^−^, 283 [M–H–Hexose–CH_3_]^−^, 269 [M–H–CO–H]^−^, tetraoxygenated A-ring 161, and monooxygenated B-ring fragment 117 was characterised as scutellarein 7-methyl ether 6-galactoside.^110^ Compound 13 (peak 13), due to loss of glucuronic acid, gave a [M–H]^−^ ion at *m*/*z* 429 and subsequent fragment ion at *m*/*z* 253. Based on fragmentation spectra, accurate mass and previous literature reports, compound 13 was tentatively identified as chrysin-7-glucuronide.^111^ Compound 14 (peak 14) possessed molecular ion [M–H]^−^ at *m*/*z* 461. Furthermore, the fragment ion at *m*/*z* 299 corresponds to the loss of glucose moiety, indicating the molecular ion of peonidin aglycone. Thus, compound 14 was tentatively identified as peonidin-3-*O*-galactoside.^112^ MS/MS fragmentation spectra of the identified compounds from *A. nallamalayana* given in ESI Fig. 3.†

### 3.9 Structure elucidation of chemicals constituents of methanol extract of *A. nallamalayana*

Three compounds were isolated in various yields from the methanol extract of *A. nallamalayana*. Compound 1 was crystallized with MeOH, which produced yellow needle crystals, mp 264–265°. The negative ion mass spectra showed [M–H]^−^ peak at *m*/*z* 283.09, correspondings to molecular formula C_16_H_12_O_5_. This was corroborated by the ^13^C-NMR spectrum, which showed signals for all the molecule's twelve carbons. Compound 2 was crystallized with MeOH, which gave the yellow needle-shaped crystals, mp 210–211°. Compound 2 gave [M–H]^−^ peak at *m*/*z* 313.12 in its HRMS corresponding to molecular formula C_17_H_14_O_6_, which was corroborated by ^13^C-NMR spectrum, which showed signals for all the fourteen carbons of the molecule. Compound 3, which was crystallized with MeOH, gave colourless needles, mp 138–139° showed [M–H]^−^ peak at *m*/*z* 461.11 in its negative ion mass spectra corresponding to molecular formula C_22_H_22_O_11_, corroborated by ^13^C-NMR spectrum, which showed signals for all the fourteen carbons of the molecule.

**Fig. 8:**
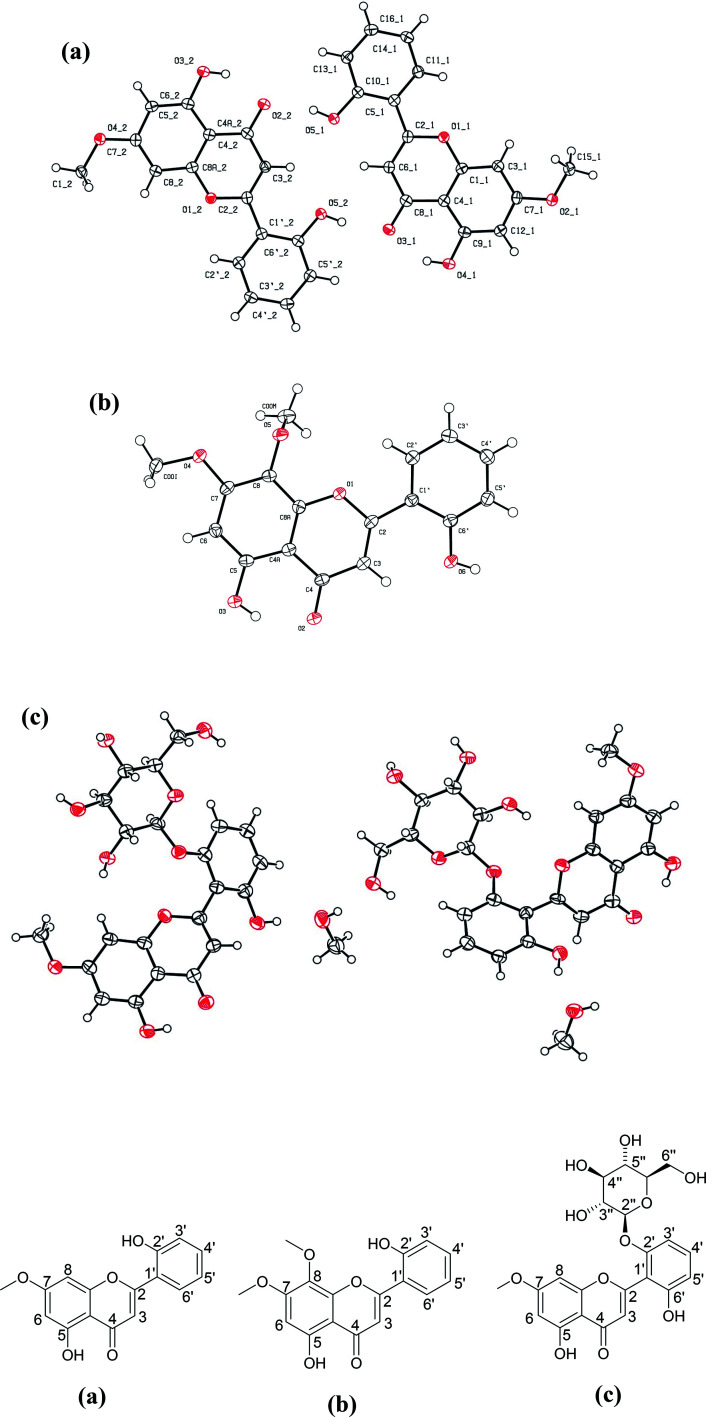
Crystal structures of three compounds isolated from *A. nallamalayana*. (a) Echioidinin (CCDC deposition no. 2072153); (b) skullcapflavone I (CCDC deposition no. 2072155); and (c) 5,2′,6′-trihydroxy-7-methoxyflavone 2′-*O*-β-d-glucopyranoside with methanol as solvent molecule (CCDC deposition no. 2072714).

The structures of three known compounds were characterised as echioidinin^113^ (compound 1), skullcapflavone I^114^ (compound 2), and 5,2′,6′-trihydroxy-7-methoxyflavone 2′-*O*-β-d-glucopyranoside^108^ (compound 3) based on their single-crystal X-ray diffraction (XRD) (Table 4) and comparison of their spectral data with literature (Table 5). The crystal structures of echioidinin (CCDC deposition no. 2072153); skullcapflavone I (CCDC deposition no. 2072155), and 5,2′,6′-trihydroxy-7-methoxyflavone 2′-*O*-β-d-glucopyranoside (CCDC deposition no. 2072714) were reported for the first time (Fig. 8). The crystal of compound 3 was twinned and treated accordingly with HKL5 format. The final residual factors or discrepancy indices (*R*_1_ values) of compound 3 was 6.95% which was due to the limited quality of the data. All crystals' thermal ellipsoid plot was represented in ESI along with the CheckCif alerts (Fig. S5, S11, and S17).†

**Table 4:** Crystallography data of three compounds isolated from *A. nallamalayana*

CCDC no.	2072153	2072155	2072714
Identification code	ANM_18_23_ Echioidinin_0m_a	PJ_KUC_0m_a_a	ANM_28_ML_25_0m_a
Empirical formula	C_16_H_12_O_5_	C_17_H_14_O_6_	C_22_H_22_O_11_
Formula weight	284.26	314.28	462.44
Temperature/K	100	100	100.0
Crystal system	Monoclinic	Monoclinic	Monoclinic
Space group	*P*2_1_/*n*	*P*2_1_/*c*	*P*2_1_
*a*/Å	7.0308(2)	4.9210(11)	11.5283(7)
*b*/Å	14.1809(4)	21.735(4)	8.0352(5)
*c*/Å	24.9877(6)	13.1847(11)	24.3766(15)
*α*/°	90	90	90
*β*/°	92.5360(10)	96.263(11)	103.495(4)
*γ*/°	90	90	90
Volume/Å^3^	2488.91(12)	1401.8(4)	2195.7(2)
*Z*	8	4	4
*ρ*_calc_g/cm^3^	1.517	1.489	1.496
*μ*/mm^−1^	0.953	0.959	1.044
*F*(000)	1184	656	1040.0
Crystal size/mm^3^	0.75 × 0.28 × 0.14	0.21 × 0.19 × 0.11	0.06 × 0.05 × 0.04
Radiation	CuKα (*λ* = 1.54178)	CuKα (*λ* = 1.54184)	CuKα (*λ* = 1.54178)
2*Θ* range for data collection/°	7.082 to 133.246	7.878 to 132.424	3.728 to 134.698
Index ranges	−8 ≤ *h* ≤ 6, −16 ≤ *k* ≤ 16, −29 ≤ *l* ≤ 29	−5 ≤ *h* ≤ 5, −25 ≤ *k* ≤ 25, −15 ≤ *l* ≤ 14	−13 ≤ *h* ≤ 13, −9 ≤ *k* ≤ 9, −29 ≤ *l* ≤ 28
Reflections collected	37 622	29 097	75 053
Independent reflections	4341 [*R*_int_ = 0.0634, *R*_sigma_ = 0.0354]	2436 [*R*_int_ = 0.0581, *R*_sigma_ = 0.0277]	7767 [*R*_int_ = 0.1096, *R*_sigma_ = 0.0547]
Data/restraints/parameters	4341/0/386	2436/0/212	7767/1/653
Goodness-of-fit on *F*^2^	1.113	1.066	1.090
Final *R* indexes [*I* ≥ 2*σ*(*I*)]	*R*_1_ = 0.0463, w*R*_2_ = 0.1237	*R*_1_ = 0.0442, w*R*_2_ = 0.1105	*R*_1_ = 0.0695, w*R*_2_ = 0.1774
Final *R* indexes [all data]	*R*_1_ = 0.0475, w*R*_2_ = 0.1248	*R*_1_ = 0.0460, w*R*_2_ = 0.1120	*R*_1_ = 0.0724, w*R*_2_ = 0.1816
Largest diff. peak/hole/e Å^−3^	0.28/−0.27	0.22/−0.28	0.46/−0.40
Flack parameter	NA	NA	?

**Table 5:** NMR data (DMSO-*d*_6_, ^1^H 600 MHz and ^13^C 150 MHz) of compounds isolated from *A. nallamalayana*

Position	1		2		3	
	*δ*_H_ (*J* in Hz)	*δ*_C_	*δ*_H_ (*J* in Hz)	*δ*_C_	*δ*_H_ (*J* in Hz)	*δ*_C_
2	—	161.99, C		161.58, C	—	162.24, C
3	7.11 (s)	109.62, CH	7.08 (s)	108.79, CH	6.32 (s)	113.01, CH
4	—	182.54, C	—	182.34, C	—	182.56, C
5	—	161.50, C	—	156.57, C	—	161.71, C
6	6.37 (d, *J* = 2.04)	98.36, CH	6.56 (s)	95.81, CH	6.39 (d, *J* = 2.4)	98.35, CH
7	—	165.67, C	—	158.42, C	—	165.64, C
8	6.76 (d, *J* = 1.5)	93.03, CH	—	128.40, C	6.61 (d, *J* = 2.4)	93.13, CH
9	—	157.86, C	—	148.90, C	—	158.87, C
10	—	105.11, C	—	103.58, C	—	105.54, C
1′	—	117.52, C	—	117.13, C	—	110.69, C
2′	—	157.32, C	—	156.93, C	—	157.00, C
3′	6.99 (t, *J* = 7.5 14.94)	117.43, CH	7.06–7.02 (m)	117.21, CH	6.73 (d, *J* = 8.4)	106.24, CH
4′	7.40 (td, *J* = 7.12, 15.28)	133.41, CH	7.43 (m)	133.07, CH	7.28 (t, *J* = 8.4, 16.6)	132.74, CH
5′	7.07 (d, *J* = 8.2)	119.85, CH	7.06–7.02 (m)	119.64, CH	6.65 (d, *J* = 8.4)	110.08, CH
6′	7.90 (dd, *J* = 1.6, 8.02)	128.93, CH	7.85 (dd, *J* = 7.87, 1.0)	128.26, CH	—	156.77, C
1′′	—	—	—	—	4.90 (d, *J* = 7.8)	101.12, CH
2′′	—	—	—	—	3.06 (m)	73.71, CH
3′′	—	—	—	—	3.21 (m)	77.22, CH
4′′	—	—	—	—	3.11 (br d)	70.13, CH
5′′	—	—	—	—	3.43 (br d)	77.63, CH
6′′	—	—	—	—	3.68 (br d), 3.44 (m)	61.19, CH_2_
O–CH_3_-7	3.85 (s)	56.48, CH_3_	3.89 (s)	56.46, CH_3_	3.84 (s)	56.56, CH_3_
O–CH_3_-8	—	—	3.80 (s)	61.10, CH_3_	—	—
OH-5	12.86 (s)	—	12.67 (s)	—	12.92 (s)	—
OH-2′	10.91 (s)	—	8.27 (s)	—	—	—
OH-6′	—	—	—	—	10.13 (s)	—

### 3.10 HPLC-UV analysis of methanolic leaf extract of *A. nallamalayana*

**Fig. 9:**
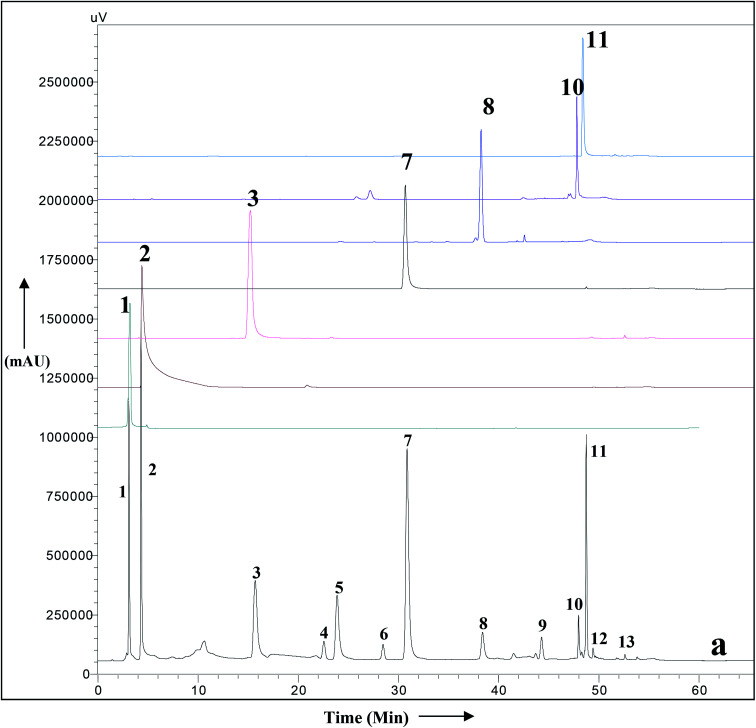
HPLC-UV chromatogram of the total methanolic extract of *A. nallamalayana* aerial parts (a), and authentic commercial standard/purified compounds: (1) chlorogenic acid; (2) 3,4-di-*O*-caffeoylquinic acid; (3) hispidulin 7-glucoside (homoplantaginin); (7) 5,2′,6′-trihydroxy-7-methoxyflavone 2′-*O*-β-d-glucopyranoside; (8) oroxylin A; (10) skullcapflavone I; (11) echioidinin. Mobile phase: water (A) and acetonitrile (B) with a gradient system, *i.e.*, 0–40 min, 0–70% B; 40–50 min, 70–100% B; 50–60 min, 100% B; 60–65 min, 0% B, flow 1 ml min^−1^, for other chromatographic conditions, see experimental part.

A simple RP HPLC method with a gradient of acetonitrile and water was used for the simultaneous identification of secondary metabolites of *A. nallamalayana*. The HPLC-UV chromatogram of methanolic leaf extract of *A. nallamalayana* showed 13 major and minor peaks (Fig. 9). Seven major peaks, peaks 1 and 2, respectively, identified as phenolic acids that correspond to chlorogenic acid (*λ* = 217, 240sh, 324 nm, rt: 3.08 min), and 3,4-di-*O*-caffeoylquinic acid (*λ* = 243, 303sh, 325 nm, rt: 4.29 min). Peaks 3 and 7 were identified as flavonoid glucoside, which corresponded to hispidulin 7-glucoside (homoplantaginin) (*λ* = 226, 334 nm, rt: 15.09 min), and 5,2′,6′-trihydroxy-7-methoxyflavone 2′-*O*-β-d-glucopyranoside (*λ* = 275, 327 nm, rt: 30.86 min). Peaks 8, 10, and 11 were identified as flavone derivatives corresponds to oroxylin A (*λ* = 273, 321 nm, rt: 38.39 min); skullcapflavone I (*λ* = 270, 320 nm; rt: 47.980 min) and echioidinin (*λ* = 265, 335 nm, rt: 48.742 min), respectively. HPLC-UV analysis revealed that flavonoids and phenolics were the main components of methanolic leaf extract of *A. nallamalayana*, which were also characterised by UPLC-QTOF-MS/MS analysis. Flavonoids are the most studied class of plant's secondary metabolites with well-defined physical and chemical properties. Flavonoids give a characteristic UV absorption pattern, making their UV/PDA spectra very distinctive and UV spectroscopy a preferred analytical tool for identification.^115^ Two characteristic bands observed in UV spectra of flavonoids, band I (*λ*_max_ 300–380 nm) is caused by ring B absorption, while band II (*λ*_max_ 240–280 nm) is caused by ring A absorption. These bands' location provides details on the class of flavonoids as well as their substitution pattern; hence, UV spectroscopy has been used as the primary tool for the quantification and identification of flavonoids for years.^116^ All peaks were identified by direct comparison (retention time and UV-spectra) with authentic commercial standards and isolated compounds.

### 3.11 Prediction of the *in silico* biological activity

#### 3.11.1 *In silico* anticancer activity prediction using PASS program

In order to mitigate the complexity and expenses of experimental *in vivo* screening of anticancer agents using tens of millions of natural and synthetic chemical compounds, *in silico* phenotypic screening methods are required.^50^ We used the successfully reported PASS (Prediction of Activity Spectra for Substances) algorithm to predict the anticancer activity of all the compounds identified from the methanolic extract of *A. nallamalayana*. 20 compounds were analysed by the PASS program for antitumor effects. The results obtained by the PASS prediction are shown in ESI (Table S3).† Among the screened compounds, 17 compounds showed significant probable anticancer/antineoplastic activity (Pa ≥ 0.9), whereas three compounds were inactive (Pa < 0.9). Isorhamnetin 3-glucoside showed the highest Pa values (0.974/0.001), whereas chlorogenic acid showed the minimum Pa value (0.846/0.004). The majority of compounds belong to phenolic acids, flavonoids and their glucoside which are well known for their anticancer activities.^117–119^ As evident from the findings, the scores for probable activity (Pa) were very close to 1, and the scores for probable inactivity (Pi) were very close to 0, indicating that these compounds are highly likely to be active in the *in vitro*/*in vivo* studies.

#### 3.11.2 *In silico* cell line cytotoxicity prediction using CLC-Pred tool

CLC-Pred, a well-known tool used in chemoinformatics and medicinal chemistry to predict the *in silico* cytotoxicity for tumour and non-tumour cell lines, was used to predict the cytotoxicity of the identified compounds. The estimated results that have been presented in Pa values, which is >0.5, are probably more active with the predicted cancer cell line. From the 20 compounds selected, 14 compounds showed aspirated outcome, and barely six compounds displayed negative results (Table S4†). The cytotoxicity represented by the compounds identified from the methanolic extract of *A. nallamalayana* matches the present study and literature survey.^119–121^

## 4 Conclusion

In the present study, the methanolic extract of two *Andrographis* species, *A. nallamalayana* and *A. paniculata*, showed significant cytotoxicity towards three different cancer cell in a dose-dependent manner. The cytotoxicity of ANM was nearly four times higher than APM in HCT 116 and HepG2 cells, whereas both extracts showed comparatively similar cytotoxicity in A549 cells and no or very less cytotoxicity in HEK 293 cells. Furthermore, ANM induced cell death involves apoptotic changes and inhibition of migration, ROS generation, up-regulation and down-regulation of main apoptotic markers as seen in HCT 116 cells. Although both species showed promising cytotoxic activity, the comparative phytochemical investigation revealed that both species had different chemical constituents. Both species were found to contain significant quantities of phenolics and flavonoids. Using UPLC-QTOF-MS (HRMS) analysis, andrographolides were identified as the major compounds of *A. paniculata*. Interestingly, andrographolides were not found in *A. nallamalayana*.

Further, using the MS/MS fragmentation approach, 20 compounds were characterized/identified from *A. nallamalayana*; out of 20, 18 compounds were identified for the first time from this species. Three known compounds, echioidinin, skullcapflavone I and 5,2′,6′-trihydroxy-7-methoxyflavone 2′-*O*-β-d-glucopyranoside, were isolated from *A. nallamalayana* and their crystal structures were reported for the first time. Subsequently, seven major compounds were identified in *A. nallamalayana* by direct comparison (retention time and UV-spectra) with authentic commercial standards and isolated compounds using HPLC-UV analysis. The prediction of anticancer activity using *in silico* tools also justifies the evaluation of the *in vitro* cytotoxic activity. Our experimental studies have validated the traditional use of *A. nallamalayana* and *A. paniculata* as an anticancer herbal drug. However, more studies are required to explore the role of *A. nallamalayana* in different *in vivo* cancer models so that it can contribute to the successful treatment of cancer in future.

## Author contributions

Narender Goel: conceptualization, methodology, investigation, writing-original draft. Rahul L. Gajbhiye: conceptualization, methodology, investigation, data curation, software. Moumita Saha: methodology, writing, formal analysis, data curation. Chennuru Nagendra: resources validation. Araveeti Madhusudhana Reddy: resources, validation, review & editing. V. Ravichandiran: project administration, funding acquisition, review & editing. Krishna Das Saha: formal analysis, data curation, writing-review & editing, validation. Parasuraman Jaisankar: supervision, conceptualization, project administration, writing-review & editing.

## Conflicts of interest

There are no conflicts to declare.

## Supplementary Material

RA-011-D1RA07496B-s001

RA-011-D1RA07496B-s002
