# Supplementary material for: A comparative assessment of in vitro cytotoxic activity and phytochemical profiling of Andrographis nallamalayana J.L.Ellis and Andrographis paniculata (Burm. f.) Nees using UPLC-QTOF-MS/MS approach
Source: RSC Adv. 2021 Nov 8;11(57):35918–36. doi: 10.1039/d1ra07496b (PMC9043227; doi:10.1039/d1ra07496b)
Supplement: RA-011-D1RA07496B-s001 [file RA-011-D1RA07496B-s001.pdf]

Electronic Supplementary Material (ESI) for RSC Advances.
This journal is © The Royal Society of Chemistry 2021

Supplementary Information (ESI)

A comparative assessment of *in-vitro* cytotoxic activity and phytochemical profiling of *Andrographis nallamalayana* J.L.Ellis and *Andrographis paniculata* (Burm.f.) Nees using UPLC-QTOF-MS/MS approach

Narender Goel[d,§], Rahul L. Gajbhiye[a,§], Moumita Saha[b,§], Chennuru Nagendra[c], Araveeti Madhusudhana Reddy[c], V. Ravichandiran[a], Krishna Das Saha[b], Parasuraman Jaisankar[d]*

[a]*Department of Natural Products, National Institute of Pharmaceutical Educational and Research (NIPER), ChunilalBhawan, 168, Maniktala Main Road, 700054, Kolkata, India*

[b]*Cancer Biology and Inflammatory Disorder Division, CSIR–Indian Institute of Chemical Biology, 4, Raja S C Mullick Road, Jadavpur, Kolkata-700032, India.*

[c]*Department of Botany, Yogi Vemana University, Vemanapuram, Kadapa, Andhra Pradesh 516005, India.*

[d]*Laboratory of Catalysis and Chemical Biology, Department of Organic and Medicinal Chemistry, CSIR*

***Corresponding author**

Prof. Dr. Parasuraman Jaisankar

Laboratory of Catalysis and Chemical Biology, Department of Organic and Medicinal Chemistry, CSIR-Indian Institute of Chemical Biology, 4 Raja S. C. Mullick Road, Kolkata - 700032, India.

Tel.: +91-33-24995-790/774

E-mail address: jaisankar@iicb.res.in

Table of Contents

Table S1. Compounds identified by UPLC-QTOF-MS (HRMS) analysis in methanolic extract of *A. nallampalayana*. S1

Table S2. Compounds identified by UPLC-QTOF-MS (HRMS) analysis in methanolic extract of *A. paniculata*. S3

Table S3. *In-silico* PASS prediction activity of compounds identified from the methanolic extract of *A. nallampalayana*. S5

Table S4. In-silico CLC-Pred cell line cytotoxicity prediction of compounds identified from the methanolic extract of *A. nallampalayana*. S6

Figure S1. Parts of studied Andrographis species **a)** Leaves of *Andrographis nallamalayana*; **b)** Leaves of *A. paniculat*....S8

Figure S2. Venn diagram for comparison of chemical constituents commonly present in both Andrographis species. **ANM)** *Andrographis nallamalayana* methanol extract; **APM)** *Andrographis paniculata* methanol extract. S8

Figure S3. MS/MS fragmentation spectra of the identified compounds from the methanolic extract of *A. nallamalayana*. S15

Figure S4. HRMS spectra of **Compound 1**. S16

Figure S5. Thermal ellipsoid plot of **Compound 1**. Ellipsoids are represented with 50% probability. S16

Figure S6. ^{1}H NMR of Compound 1 ....S17

Figure S7. ^{13}C NMR of Compound 1. ....S17

Figure S8. DEPT-90 NMR of Compound 1. ....S18

Figure S9. DEPT-135 NMR of Compound 1. ....S18

Figure S10. HRMS Spectra of Compound 2 ....S19

Figure S11. Thermal ellipsoid plot of **Compound 2**. Ellipsoids are represented with 50% probability. S19

Figure S12. ^{1}H NMR of Compound 2 ....S20

Figure S13. ^{13}C NMR of Compound 2. ....S20

Figure S14. DEPT-90 NMR of Compound 2. ....S21

Figure S15. DEPT-135 NMR Spectra of Compound 2. ....S21

Figure S16. HRMS Spectra of **Compound 3.** S22

Figure S17. Thermal ellipsoid plot of **Compound 3.** Ellipsoids are represented with 50% probability. S22

Figure S18. ^{1}H NMR of **Compound 3.** S23
Figure S19. ^{13}C NMR of **Compound 3**. S23
Figure S20. DEPT-90 NMR of **Compound 3**. S24
Figure S21. DEPT-135 NMR of **Compound 3**. S24
Figure S22: Raw validation data and unprocessed original images of gels and western blots……………………………........…………………………………...……………………..……S25

Table S1. Compounds identified by UPLC-QTOF-MS (HRMS) analysis in methanolic extract of *A. nallampalayana.*

General						Compound Identification	Species/ Adduct
**Formula**	***m/z***	**Mass**	**RT**	**Height**	**Score**	**Name**	
$C_{17}H_{16}O_7$	331.081	332.088	0.51	16508	76.38	3,4',5-Trihydroxy-3',7-dimethoxyflavanone	$[M-H]^-$
$C_{16}H_{26}O_{11}$	393.142	394.149	0.64	300110	89.8	1-(3-Methyl-2-butenoyl)-6-apiosylglucose	$[M-H]^-$
$C_{16}H_{20}O_{10}$	371.100	372.107	0.69	57779	91.72	Veranisatin C	$[M-H]^-$
$C_{18}H_{18}O_{10}$	393.082	394.089	0.70	10511	78.65	9-Hydroxy-4-methoxypsoralen 9-glucoside	$[M-H]^-$
$C_{20}H_{26}O_{13}$	473.131	474.138	0.73	14813	94.75	Trans-Caffeic acid [apiosyl-(1->6)-glucosyl] ester	$[M-H]^-$
$C_{16}H_{28}O_{11}$	395.156	396.163	0.82	7852	78.82	1-(3-Methylbutanoyl)-6-apiosylglucose	$[M-H]^-$
$C_{16}H_{22}O_{10}$	373.115	374.122	0.86	15911	75.63	Gardoside	$[M-H]^-$
$C_{14}H_{20}O_8$	315.110	316.117	0.91	91864	91.87	Vanilloloside	$[M-H]^-$
$C_{16}H_{24}O_{10}$	375.131	376.138	0.96	227160	89.37	(1RS,2RS)-Guaiacylglycerol 1-glucoside	$[M-H]^-$
$C_{15}H_{20}O_{10}$	359.099	360.107	0.97	20193	78.75	6'-Methoxypolygoacetophenoside	$[M-H]^-$
$C_{13}H_{16}O_8$	299.078	300.085	1.14	26797	77.01	Dimethyl fukiic acid	$[M-H]^-$
$C_{19}H_{18}O_{10}$	405.081	406.089	1.53	23613	80.03	Lancerin	$[M-H]^-$
$C_{16}H_{18}O_9$	353.090	354.318	1.66	44017	94.05	Chlorogenic Acid	$[M-H]^-$
$C_{16}H_{18}O_9$	353.090	354.316	1.81	91792	86.26	1-*O*-Caffeoylquinic acid	$[M-H]^-$
$C_{22}H_{22}O_{12}$	477.105	478.421	2.21	52316	88.11	Isorhamnetin 3-glucoside	$[M-H]^-$
$C_{25}H_{24}O_{12}$	515.122	516.454	2.48	22200	92.99	3,4-Di-*O*-caffeoylquinic acid	$[M-H]^-$
$C_{22}H_{22}O_{11}$	461.111	462.433	2.83	47475	97.22	Hispidulin 7-glucoside (Homoplantaginin)	$[M-H]^-$
$C_{16}H_{26}O_8$	345.157	346.164	3.67	74442	77.51	Glucosyl 6-hydroxy-2,6-dimethyl-2E,7-octadienoate	$[M-H]^-$
$C_{28}H_{32}O_{17}$	639.158	640.165	3.69	21080	89.09	Rhamnetin 3-sophoroside	$[M-H]^-$
$C_{23}H_{24}O_{12}$	491.121	492.128	3.81	109228	91.53	Andrographidine B	$[M-H]^-$
$C_{21}H_{20}O_{11}$	447.095	448.465	3.82	8134	82.2	Luteolin 4'-glucoside	$[M-H]^-$
$C_{28}H_{24}O_{13}$	567.118	568.512	3.94	615443	89.46	Neobignonoside	$[M-H]^-$
$C_{23}H_{22}O_{11}$	473.110	474.118	4.18	113468	92.79	Betavul garinglucoside	$[M-H]^-$
$C_{25}H_{24}O_{12}$	515.121	516.128	4.20	194542	92.11	1,3-Dicaffeoylquinic acid	$[M-H]^-$
$C_{30}H_{36}O_{14}$	619.202	620.209	4.31	8631	87.56	Matsutakeside I	$[M-H]^-$
$C_{27}H_{22}O_{12}$	537.103	538.110	4.39	57544	98.52	Melitric acid A	$[M-H]^-$
$C_{27}H_{34}O_{12}$	549.199	550.206	4.40	12572	74.15	Eucommin A	$[M-H]^-$
$C_{26}H_{32}O_{11}$	519.188	520.195	4.40	10682	73.58	(7'S,8'S)-4,7'-Epoxy-3,8'-bilign-7-ene-3',5-dimethoxy-4',9,9'-triol 4'-glucoside	$[M-H]^-$
$C_{28}H_{32}O_{16}$	623.163	624.170	4.41	17113	88.25	6,8-Diglucosyldiosmetin	$[M-H]^-$
$C_{30}H_{26}O_{15}$	625.121	626.128	4.53	10593	91.65	6''-Caffeoylhyperin	$[M-H]^-$
$C_{22}H_{22}O_{11}$	461.112	462.443	4.62	87630	98.93	5,2',6'-Trihydroxy-7-methoxyflavone 2'-*O*-β-D-glucopyranoside	$[M-H]^-$

$C_{22}H_{22}O_{11}$	461.112	462.122	4.77	96037	79.40	Luteolin 7-methyl ether 5-glucoside	[M-H]$^-$
$C_{21}H_{30}O_{8}$	409.188	410.195	4.91	5251	70.08	Pteroside D	[M-H]$^-$
$C_{16}H_{18}O_{8}$	337.095	338.315	4.94	20853	95.20	3-p-Coumaroylquinic acid	[M-H]$^-$
$C_{22}H_{22}O_{11}$	461.112	462.408	5.07	28512	96.26	Scutellarein 7-methyl ether 6-galactoside	[M-H]$^-$
$C_{28}H_{24}O_{12}$	551.121	552.128	5.40	46234	94.19	Schizotenuin F	[M-H]$^-$
$C_{29}H_{26}0_{13}$	581.133	582.512	5.43	133130	94.07	2''-*O*-Vanilloylvitexin	[M-H]$^-$
$C_{36}H_{38}O_{16}$	725.210	726.217	5.52	10093	92.36	Licorice glycoside A	[M-H]$^-$
$C_{23}H_{22}O_{10}$	457.115	458.122	5.52	129601	94.23	6''-*O*-Acetyldaidzin	[M-H]$^-$
$C_{37}H_{50}O_{20}$	813.283	814.290	5.60	13846	93.31	Jionoside B1	[M-H]$^-$
$C_{26}H_{38}O_{13}$	557.225	558.233	5.68	4417	72.58	Glaucarubol 15-*O*- β -D-glucopyranoside	[M-H]$^-$
$C_{30}H_{30}O_{15}$	629.149	630.156	5.74	5453	70.59	4-(4-Hydroxyphenyl)-2-butanone O-[2,6-digalloylglucoside]	[M-H]$^-$
$C_{21}H_{18}O_{11}$	445.079	446.086	6.06	92483	94.37	Apigenin 7-*O*-β-glucuronide	[M-H]$^-$
$C_{23}H_{24}O_{11}$	475.126	476.134	6.08	471825	87.79	Andrographidine D	[M-H]$^-$
$C_{21}H_{18}O_{10}$	429.085	430.476	6.13	49990	97.21	Chrysin 7-glucuronide	[M-H]$^-$
$C_{23}H_{24}O_{11}$	475.126	476.133	6.22	88020	94.01	Andropaniculoside A	[M-H]$^-$
$C_{25}H_{24}O_{10}$	483.131	484.138	6.34	16685	94.09	Silidianin	[M-H]$^-$
$C_{27}H_{30}O_{14}$	577.156	578.162	6.51	8783	77.34	Isorhoifolin	[M-H]$^-$
$C_{28}H_{30}O_{12}$	557.165	558.173	6.53	6822	90.27	Physalin K	[M-H]$^-$
$C_{25}H_{24}O_{13}$	531.115	532.123	6.75	106330	93.55	2'',6''-Diacetylorientin	[M-H]$^-$
$C_{26}H_{24}O_{13}$	543.113	544.120	6.86	14752	92.54	Maclurin 3-C-(6''-p-hydroxybenzoyl-glucoside)	[M-H]$^-$
$C_{15}H_{10}O_{4}$	253.051	254.059	6.86	21428	80.47	Apigenin	[M-H]$^-$
$C_{22}H_{22}O_{11}$	461.110	462.117	7.18	102038	84.27	Peonidin 3-galactoside	[M-H]$^-$
$C_{16}H_{12}O_{5}$	283.063	284.268	7.33	6210	95.18	Echioidinin	[M-H]$^-$
$C_{16}H_{12}O_{5}$	283.063	284.266	7.96	85845	88.07	Oroxylin A	[M-H]$^-$
$C_{16}H_{12}O_{5}$	283.063	284.268	8.36	7577	80.48	Wogonin	[M-H]$^-$
$C_{17}H_{14}O_{6}$	313.073	314.291	8.56	3740	75.86	3,5-Dihydroxy-7,8-dimethoxyflavone	[M-H]$^-$
$C_{17}H_{14}O_{6}$	313.073	314.291	10.65	24355	82.89	Skullcapflavone I	[M-H]$^-$
$C_{16}H_{12}O_{5}$	283.062	284.266	11.11	67233	91.27	7,2'-Dihydroxy-5-methoxyflavone	[M-H]$^-$

Table S2. Compounds identified by UPLC-QTOF-MS (HRMS) analysis in methanolic extract of *A. paniculata.*

General						Compound Identification	Species/ Adduct
**Formula**	***m/z***	**Mass**	**RT**	**Height**	**Score**	**Name**	
$C_{20}H_{28}O_6$	363.430	364.438	0.68	13905	80.57	Andrographic acid	$[M-H]^-$
$C_{17}H_{16}O_6$	315.082	316.09	0.91	12662	77.28	(2S)-5,2′-dihydroxy-7,8-dimethoxyflavanone	$[M-H]^-$
$C_{18}H_{16}O_8$	359.076	360.084	1.69	451509	92.66	Rosmarinic acid	$[M-H]^-$
$C_{23}H_{24}O_{12}$	491.121	492.128	3.81	18922	79.45	Andrographidine B	$[M-H]^-$
$C_{26}H_{42}O_{12}$	511.543	512.6215	3.95	22746	74.5	Andrographiside	$[M-H]^-$
$C_{16}H_{24}NO_5$	299.092	300.321	4.25	15689	75.95	Onysilin	$[M-H]^-$
$C_{20}H_{30}O_5$	349.203	350.210	4.38	155834	92.41	Andropanolide	$[M-H]^-$
$C_{25}H_{28}O_{13}$	535.451	536.482	4.39	130370	95.97	Andrographidine F	$[M-H]^-$
$C_{25}H_{24}O_{12}$	515.120	516.128	4.42	205579	96.1	1,3-Dicaffeoylquinic acid	$[M-H]^-$
$C_{23}H_{25}O_{11}$	476.131	477.139	4.53	88481	84.67	7,8-Dimethoxy-2'-hydroxy-5-O-β-D-glucopyranosyloxyflavone	$[M-H]^-$
$C_{21}H_{18}O_{11}$	445.079	446.086	4.61	215410	94.6	Apigenin 7-O-β-glucuronide	$[M-H]^-$
$C_{26}H_{40}O_{10}$	511.261	512.262	4.71	133661	92.86	Andrographiside	$[M-H]^-$
$C_{16}H_{18}O_9$	353.089	354.096	4.74	37153	91.31	Chlorogenic acid	$[M-H]^-$
$C_{22}H_{22}O_{11}$	461.110	462.118	4.78	8404	72.82	5,2’,6’-trihydroxy-7-methoxyflavone 2’-*O*- β -D-glucopyrdnoside	$[M-H]^-$
$C_{26}H_{40}O_8$	479.653	480.653	4.84	197830	92.17	3-*O*-β-D-glucopyranosyl-14,19-dideoxyandrographolide	$[M-H]^-$
$C_{22}H_{22}O_{10}$	445.400	446.408	5.03	79466	89.45	Isoswertisin	$[M-H]^-$
$C_{22}H_{22}O_{11}$	461.110	462.117	5.08	46906	75.91	Scutellarin-6-*O*-β-D-glucoside-7-methyl ether	$[M-H]^-$
$C_{20}H_{28}O_4$	331.193	332.200	5.23	91660	76.77	Deoxy-didehydroandrographolide	$[M-H]^-$
$C_{22}H_{22}O_{11}$	461.108	462.116	5.24	95963	93.89	Scutellarin-6- *O*-β-D -glucoside-7-methyl ether	$[M-H]^-$
$C_{22}H_{22}O_{10}$	445.114	446.122	5.26	11952	79.2	Isoswertisin	$[M-H]^-$
$C_{26}H_{40}O_{10}$	511.252	512.261	5.39	9527	76.81	3-*O*-β-D-glucopyranosyl andrographolide	$[M-H]^-$
$C_{26}H_{40}O_9$	495.262	496.273	5.5	59614	80.49	3-O-*β*-D-glucosyl-14-deoxyandrographolide	$[M-H]^-$
$C_{20}H_{32}O_6$	367.214	368.221	5.62	93418	90.4	Andrographolic acid	$[M-H]^-$
$C_{21}H_{20}O_{10}$	431.097	432.105	5.63	26788	87.72	5,4'-Dihydroxy-7-methoxy-8-*O*-*β*-D-glucopyranosyloxyflavone	$[M-H]^-$
$C_{25}H_{24}O_{12}$	515.120	516.128	5.7	25155	93.26	3,4-Dicaffeoylquinic acid	$[M-H]^-$
$C_{27}H_{28}O_{12}$	543.508	544.509	5.71	5238	75.88	Methyl-3,4-dicaffeoylquinate	$[M-H]^-$
$C_{20}H_{28}O_5$	347.187	348.194	5.92	54449	78.19	Deoxy-oxoandrographolide	$[M-H]^-$
$C_{23}H_{24}O_{11}$	475.126	476.134	6.08	47182	79.87	Andrographidine D	$[M-H]^-$
$C_{19}H_{26}O_4$	317.176	318.184	6.17	11366	78.14	14-deoxy-11,12-dihydroandrographolide	$[M-H]^-$
$C_{23}H_{24}O_{11}$	475.126	476.133	6.20	90020	91.24	Andropaniculoside A	$[M-H]^-$
$C_{20}H_{30}O_5$	349.203	350.210	6.29	227466	96.25	Andrographolide	$[M-H]^-$

$C_{18}H_{16}O_6$	327.082	328.094	6.7	19122	84.01	5-hydroxy-2′,7,8-trimethoxyflavone	$[M-H]^-$
$C_{26}H_{40}O_9$	495.260	496.2688	6.79	198832	92.7	Ninandrographolide	$[M-H]^-$
$C_{20}H_{28}O_4$	331.192	332.198	7.45	80862	77.78	14-deoxy-14,15-didehydroandrographolide	$[M-H]^-$
$C_{17}H_{14}O_6$	313.290	314.293	8.45	4881	79.85	Skullcapflavone I	$[M-H]^-$
$C_{21}H_{32}O_5$	363.217	364.224	8.79	39050	93.95	14-deoxy-12-methoxyandrographolide	$[M-H]^-$
$C_{26}H_{32}O_8$	283.263	284.262	9.14	4902	76.2	Oroxylin A	$[M-H]^-$
$C_{20}H_{24}O_2$	295.420	296.421	9.19	1096	84.42	Andrographolactone	$[M-H]^-$
$C_{19}H_{28}O_6$	351.188	352.189	9.67	11524	78.39	7-Hydroxy-14-deoxyandrographolide	$[M-H]^-$
$C_{18}H_{18}O_5$	313.142	314.115	11.69	7749	73.11	5,7,8-Trimethoxydihydroflavone	$[M-H]^-$
$C_{17}H_{14}O_6$	313.282	314.290	12.95	12906	80.30	Dihydroxydimethoxyflavone	$[M-H]^-$
$C_{20}H_{28}O_5$	347.429	348.430	17.99	4590	73.36	Dehydroandrographoline	$[M-H]^-$

Table S3. *In-silico* PASS prediction activity of compounds identified from the methanolic extract of *A. nallampalayana.*

**Sr. No**	**Compound Name**	**Pa***	**Pi***	**PASS Activity Prediction**
1	Isorhamnetin 3-glucoside	0.974	0.001	Anticarcinogenic
2	3-p-Coumaroyl quinic acid	0.961	0.001	Antimutagenic
3	3,4-Di-*O*-caffeoylquinic acid	0.955	0.001	Antimutagenic
4	2''-*O*-Vanilloylvitexin	0.949	0.002	Chemopreventive
5	Luteolin 4'-glucoside	0.948	0.002	Anticarcinogenic
6	Scutellarein 7-methyl ether 6-galactoside	0.947	0.002	Chemopreventive
7	Hispidulin 7-glucoside	0.943	0.002	Chemopreventive
8	Peonidin 3-*O*-galactoside	0.942	0.002	Chemopreventive
9	Wogonin	0.94	0.001	Antimutagenic
10	Luteolin 7-methyl ether 5-β-D-glucoside	0.924	0.002	Anticarcinogenic
11	3,5-Dihydroxy-7,8-dimethoxyflavone	0.917	0.002	Antimutagenic
12	1-*O*-caffeoylquinic acid	0.915	0.002	Antimutagenic
13	Neobignonoside	0.91	0.002	Anticarcinogenic
14	5,2’,6’-Trihydroxy-7-methoxyflavone 2’-*O*-β-D-glucopyranoside	0.91	0.002	Anticarcinogenic
15	Echioidinin	0.907	0.002	Antimutagenic
16	7,2'-Dihydroxy-5-methoxyflavone	0.906	0.002	Antimutagenic
17	Chrysin 7-glucuronide	0.9	0.002	Anticarcinogenic
18	Skullcapflavone I	0.896	0.002	Antimutagenic
19	Oroxylin A	0.881	0.002	Antimutagenic
20	Chlorogenic Acid	0.846	0.004	Anticarcinogenic

*Pa: Probable activity, Pi: Probable inactivity

Table S4. *In-silico* CLC-Pred cell line cytotoxicity prediction of compounds identified from the methanolic extract of *A. nallampalayana*.

Sr No	Identified Compound	Cell Line	Cell line full name	Tissue	Tumor Type	Pa*	Pi*
1	Chlorogenic Acid	NCI-H838	Non-small cell lung cancer	Lung	Carcinoma	0.601	0.02
		Hep G2	Hepatoblastoma	Liver	Hepatoblastoma	0.569	0.016
		RKO	Colon carcinoma	Colon	Carcinoma	0.271	0.203
2	1-*O*-Caffeoylquinic acid	NCI-H838	Non-small cell lung cancer	Lung	Carcinoma	0.581	0.023
		Hep G2	Hepatoblastoma	Liver	Hepatoblastoma	0.525	0.02
		RKO	Colon carcinoma	Colon	Carcinoma	0.273	0.198
3	Isorhamnetin 3-glucoside	HL-60	Promyeloblast leukaemia	Haematopoietic and lymphoid tissue	Leukemia	0.538	0.023
		NCI-H838	Non-small cell lung cancer	Lung	Carcinoma	0.488	0.057
		Caco-2	Colon adenocarcinoma	Colon	Adenocarcinoma	0.465	0.005
4	3,4-di-O-caffeoylquinic acid	NCI-H838	Non-small cell lung cancer	Lung	Carcinoma	0.588	0.022
		Hep G2	Hepatoblastoma	Liver	Hepatoblastoma	0.522	0.021
		LS174T	Colon adencocarcinoma	Colon	Adenocarcinoma	0.22	0.173
5	Hispidulin 7-glucoside (Homoplantaginin)	Caco-2	Colon adenocarcinoma	Colon	Adenocarcinoma	0.627	0.004
		NCI-H838	Non-small cell lung cancer	Lung	Carcinoma	0.512	0.043
		Hep G2	Hepatoblastoma	Liver	Hepatoblastoma	0.302	0.061
6	Luteolin 4'-glucoside	Caco-2	Colon adenocarcinoma	Colon	Adenocarcinoma	0.614	0.004
		NCI-H838	Non-small cell lung cancer	Lung	Carcinoma	0.505	0.047
		Hep G2	Hepatoblastoma	Liver	Hepatoblastoma	0.302	0.061
7	Neobignonoside	HL-60	Promyeloblast leukaemia	Haematopoietic and lymphoid tissue	Leukemia	0.502	0.027
		NCI-H838	Non-small cell lung cancer	Lung	Carcinoma	0.478	0.064
		Caco-2	Colon adenocarcinoma	Colon	Adenocarcinoma	0.435	0.006
8	5,2',6'-Trihydroxy-7-methoxyflavone 2'-*O*-β-D-glucopyranoside	HL-60	Promyeloblast leukaemia	Haematopoietic and lymphoid tissue	Leukemia	0.622	0.015
		NCI-H838	Non-small cell lung cancer	Lung	Carcinoma	0.505	0.047
		Caco-2	Colon adenocarcinoma	Colon	Adenocarcinoma	0.501	0.005
9	Luteolin 7-methyl ether 5-β-D-glucoside	NCI-H838	Non-small cell lung cancer	Lung	Carcinoma	0.504	0.047
		Caco-2	Colon adenocarcinoma	Colon	Adenocarcinoma	0.5	0.005
		Hep G2	Hepatoblastoma	Liver	Hepatoblastoma	0.332	0.053
10	3-p-coumaroyl quinic acid	NCI-H838	Non-small cell lung cancer	Lung	Carcinoma	0.562	0.026
		Hep G2	Hepatoblastoma	Liver	Hepatoblastoma	0.52	0.021
		SK-MEL-1	Metastatic melanoma	Skin	Melanoma	0.467	0.045
11	Scutellarein 7-methyl ether 6-	NCI-H838	Non-small cell lung cancer	Lung	Carcinoma	0.53	0.035

	galactoside	Caco-2	Colon adenocarcinoma	Colon	Adenocarcinoma	0.497	0.005
		Hep G2	Hepatoblastoma	Liver	Hepatoblastoma	0.343	0.051
12	2"-*O*-Vanilloylvitexin	NCI-H838	Non-small cell lung cancer	Lung	Carcinoma	0.51	0.044
		SK-MEL-1	Metastatic melanoma	Skin	Melanoma	0.416	0.085
		Caco-2	Colon adenocarcinoma	Colon	Adenocarcinoma	0.293	0.019
13	Chrysin 7-glucuronide	NCI-H838	Non-small cell lung cancer	Lung	Carcinoma	0.602	0.02
		SK-MEL-1	Metastatic melanoma	Skin	Melanoma	0.447	0.057
		Hep G2	Hepatoblastoma	Liver	Hepatoblastoma	0.427	0.033
14	Peonidin 3-galactoside	NCI-H838	Non-small cell lung cancer	Lung	Carcinoma	0.525	0.038
		HL-60	Promyeloblast leukaemia	Haematopoietic and lymphoid tissue	Leukemia	0.519	0.025
		Caco-2	Colon adenocarcinoma	Colon	Adenocarcinoma	0.359	0.011
15	Echioidinin	NCI-H187	Small cell lung carcinoma	Lung	Carcinoma	0.407	0.065
		COLO 320	Colon adenocarcinoma	Colon	Adenocarcinoma	0.334	0.003
		Hep G2	Hepatoblastoma	Liver	Hepatoblastoma	0.294	0.063
16	Oroxylin A	NCI-H187	Small cell lung carcinoma	Lung	Carcinoma	0.452	0.032
		Hep G2	Hepatoblastoma	Liver	Hepatoblastoma	0.409	0.037
		COLO 320	Colon adenocarcinoma	Colon	Adenocarcinoma	0.39	0.002
17	Wogonin	HOP-18	Non-small cell lung carcinoma	Lung	Carcinoma	0.426	0.023
		LS174T	Colon adencocarcinoma	Colon	Adenocarcinoma	0.302	0.033
		Hep G2	Hepatoblastoma	Liver	Hepatoblastoma	0.192	0.112
18	3,5-Dihydroxy-7,8-dimethoxyflavone	Hs 683	Oligodendroglioma	Brain	Glioma	0.408	0.111
		HOP-18	Non-small cell lung carcinoma	Lung	Carcinoma	0.321	0.063
		COLO 320	Colon adenocarcinoma	Colon	Adenocarcinoma	0.315	0.003
19	Skullcapflavone I	PC-6	Small cell lung carcinoma	Lung	Carcinoma	0.404	0.04
		Hep G2	Hepatoblastoma	Liver	Hepatoblastoma	0.386	0.042
		LS174T	Colon adencocarcinoma	Colon	Adenocarcinoma	0.337	0.016
20	7,2'-Dihydroxy-5-methoxyflavone	NCI-H187	Small cell lung carcinoma	Lung	Carcinoma	0.449	0.033
		COLO 320	Colon adenocarcinoma	Colon	Adenocarcinoma	0.408	0.002
		Hep G2	Hepatoblastoma	Liver	Hepatoblastoma	0.404	0.038

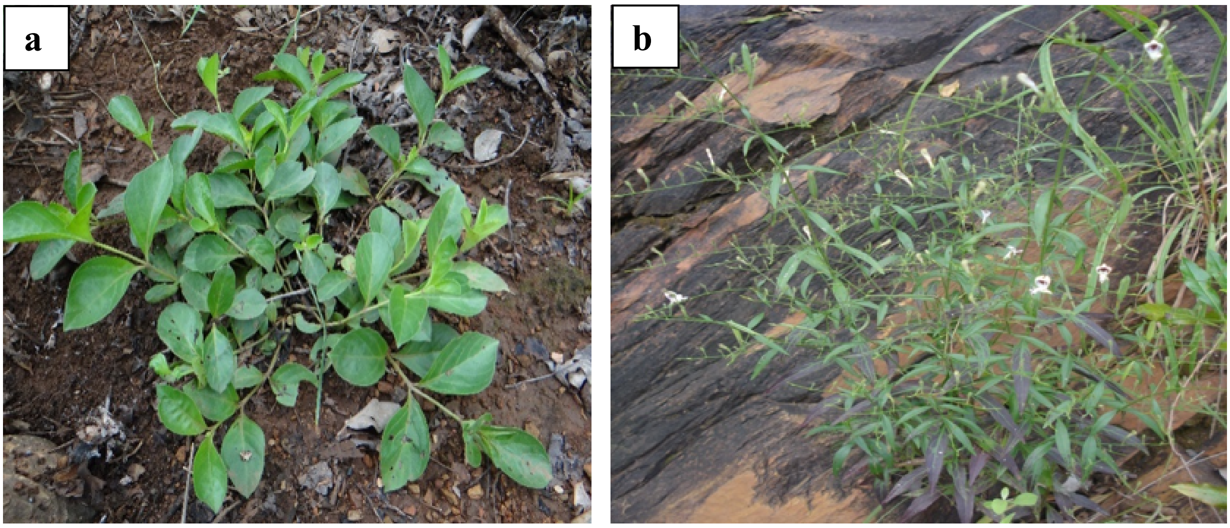

Figure S1. Parts of studied Andrographis species **a)** Leaves of *Andrographis nallamalayana*; **b)** Leaves of *Andrographis paniculata*.

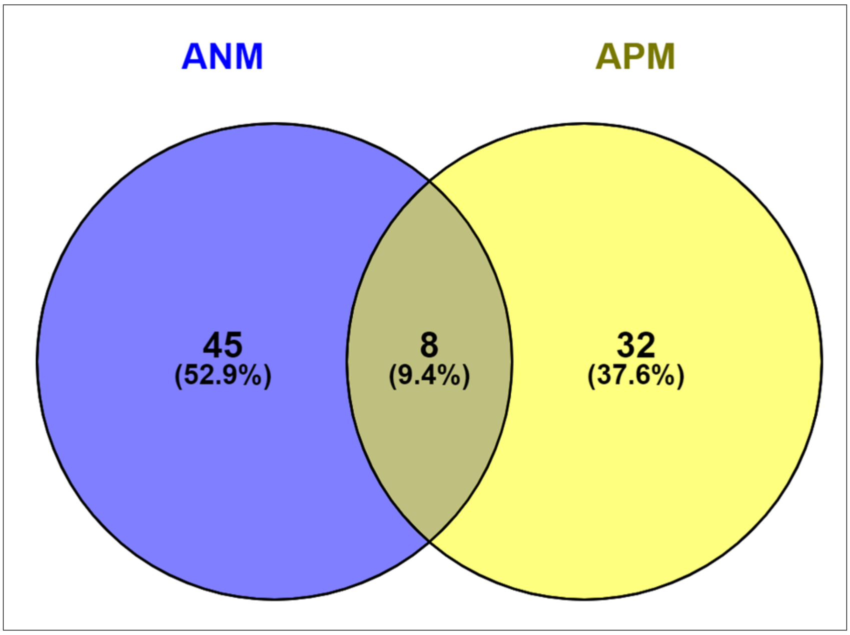

Figure S2. Venn diagram for comparison of chemical constituents commonly present in both Andrographis species. **ANM)** *Andrographis nallamalayana* methanol extract; **APM)** *Andrographis paniculata* methanol extract.

MS/MS spectra of the identified compounds

1. MS/MS Spectra of Chlorogenic Acid

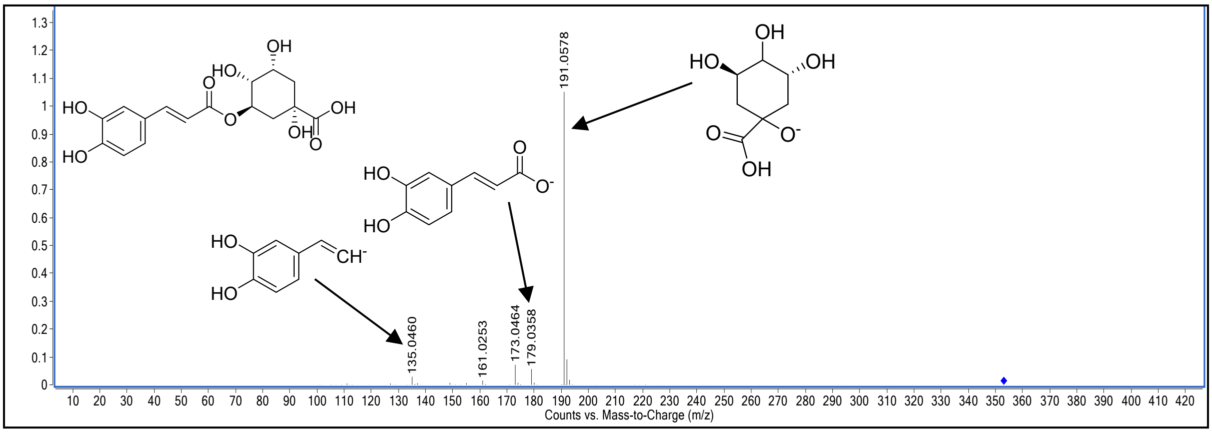

2. MS/MS Spectra of 1-*O*-Caffeoylquinic acid

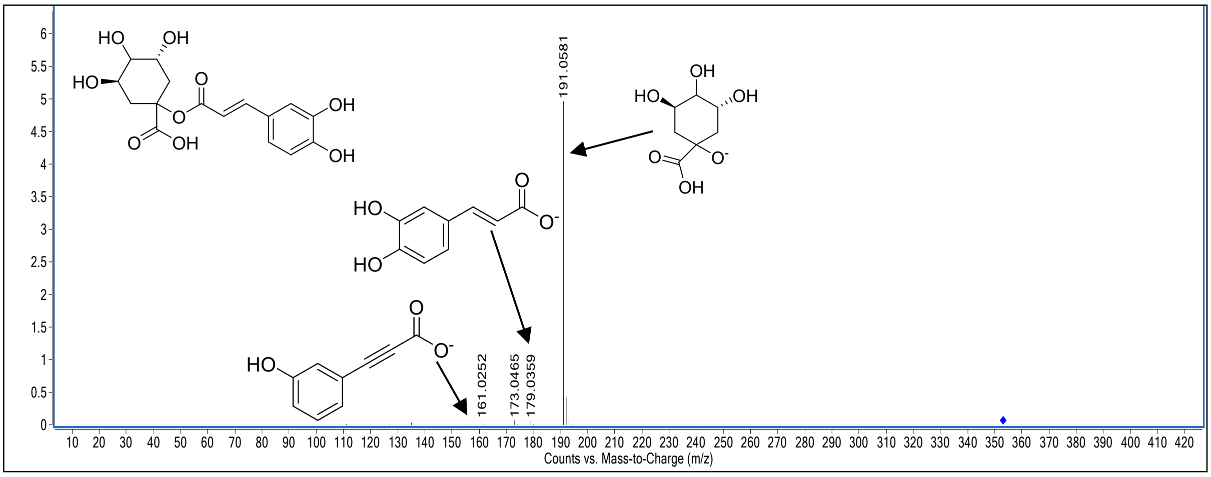

3. MS/MS Spectra of Isorhamnetin 3-glucoside

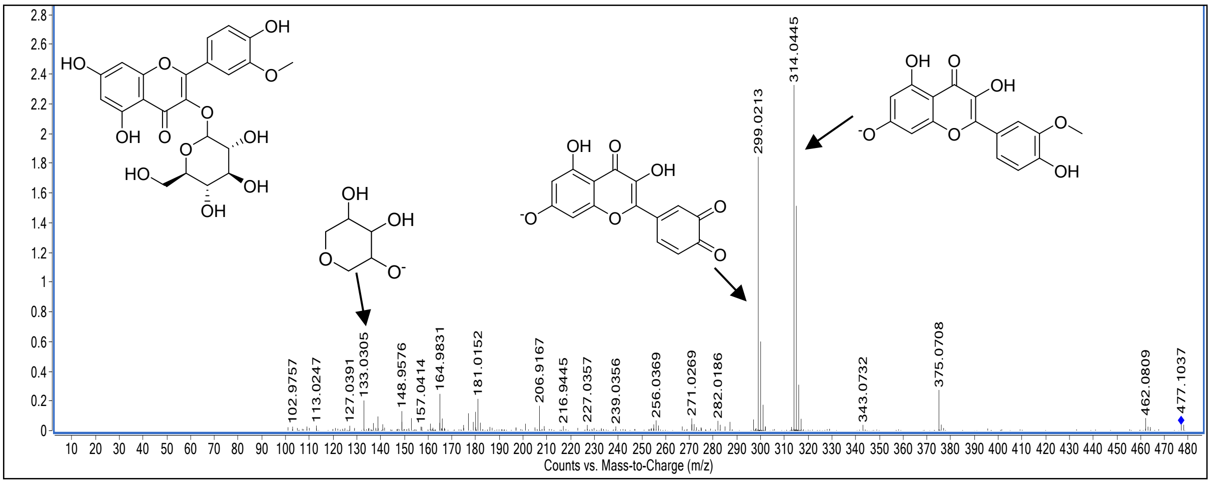

4. MS/MS Spectra of 3,4-Di-*O*-caffeoylquinic acid

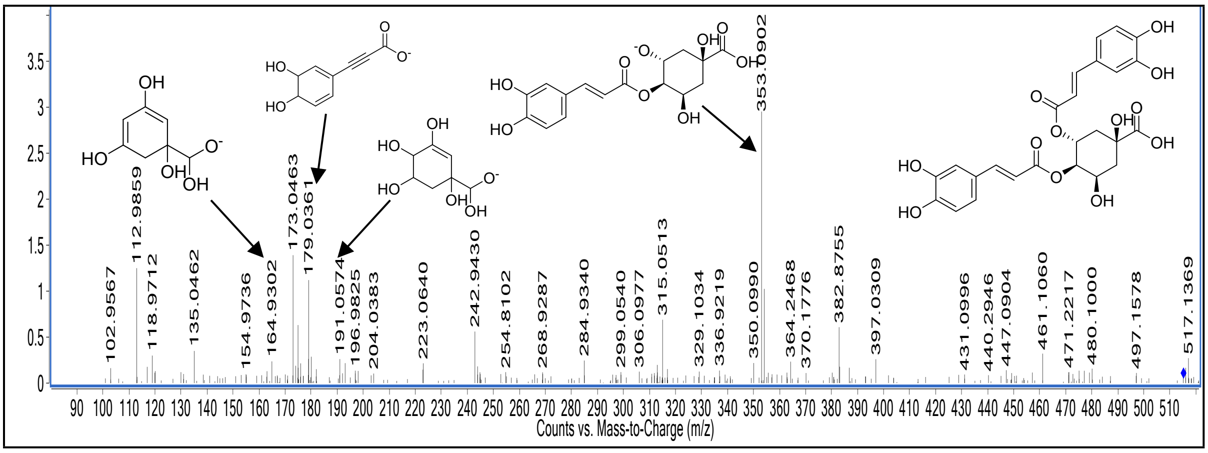

5. MS/MS Spectra of Hispidulin 7-glucoside

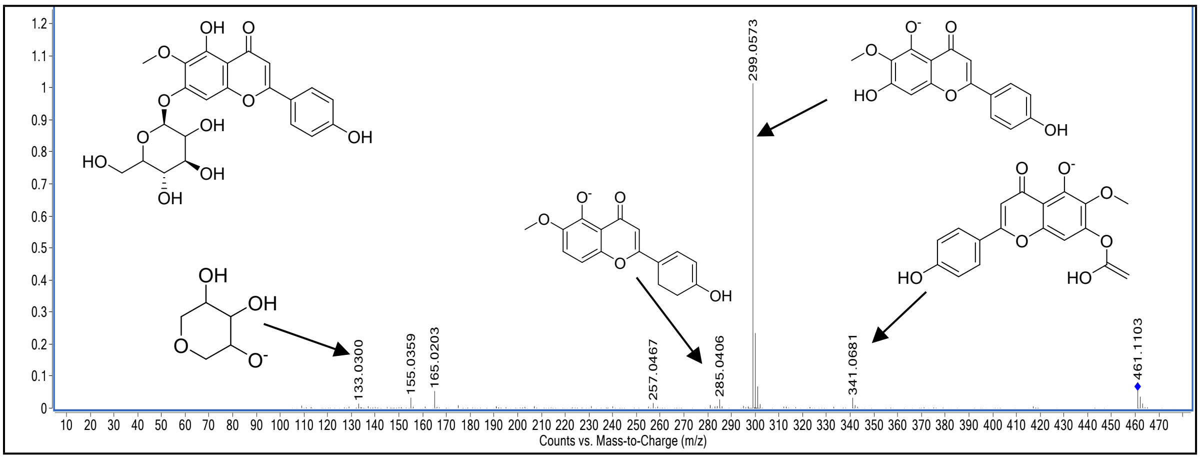

6. MS/MS Spectra of Luteolin 4'-glucoside

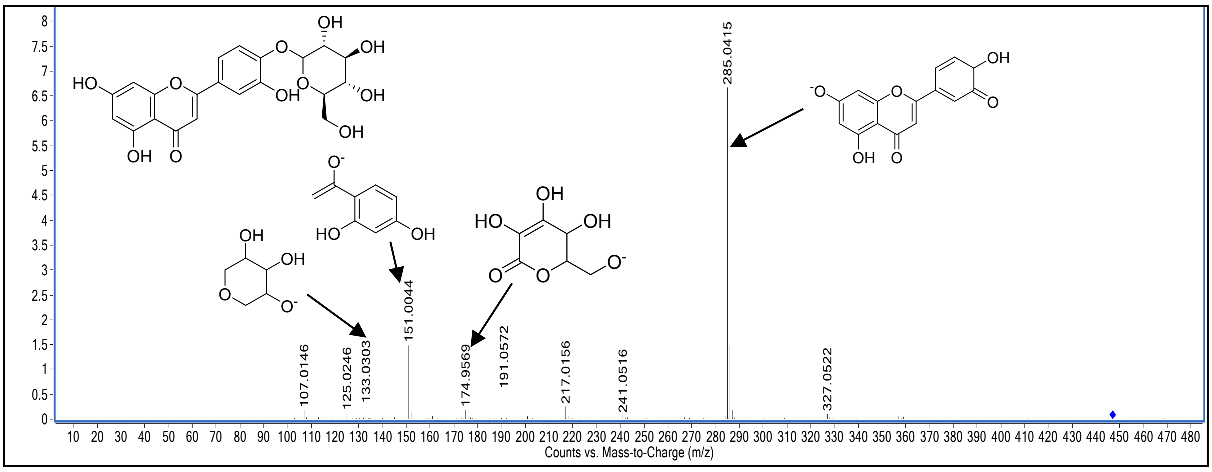

7. MS/MS Spectra of Neobignonoside

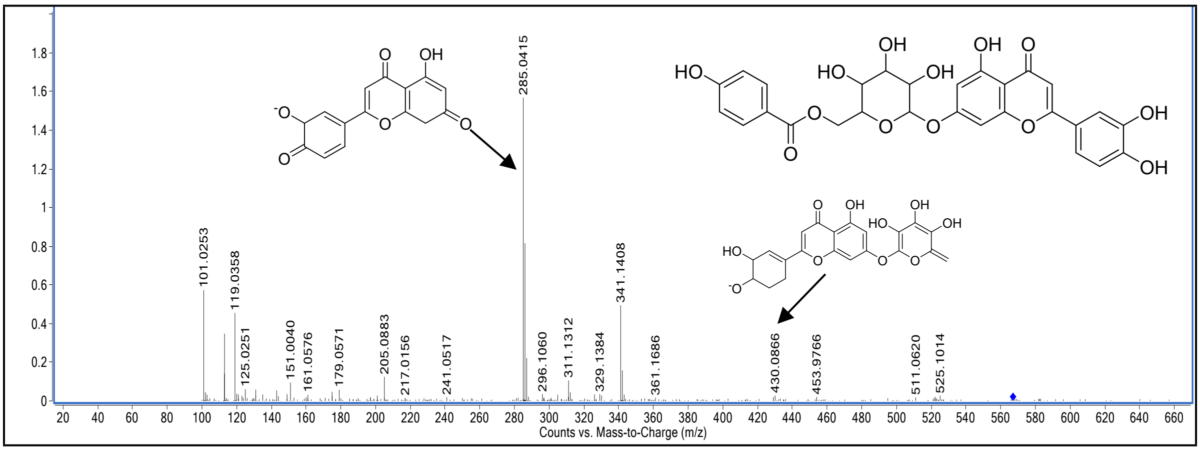

8. MS/MS Spectra of 5,2',6'-trihydroxy-7-methoxyflavone 2'-O-β-D-glucopyranoside

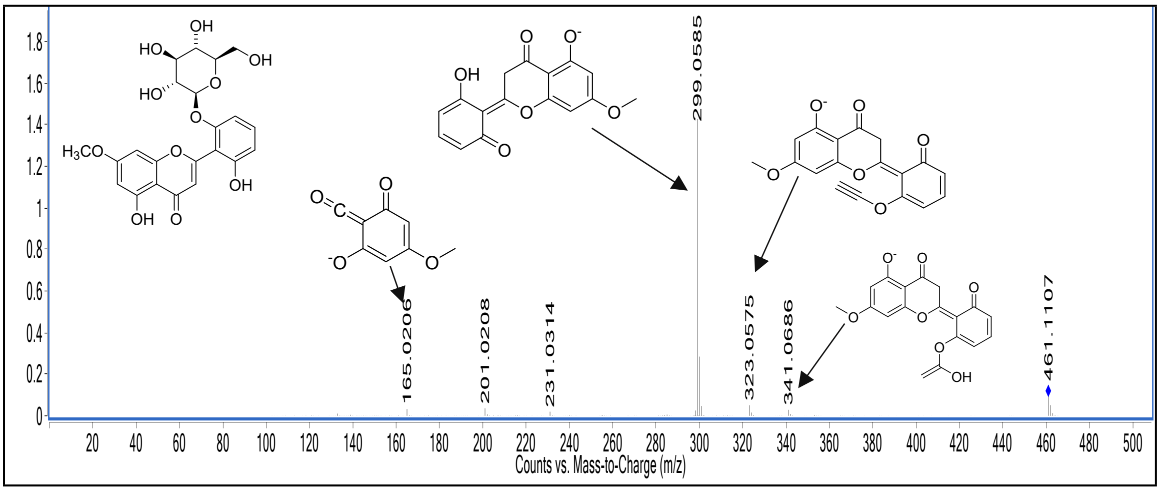

9. MS/MS Spectra of Luteolin 7-methyl ether 5-glucoside

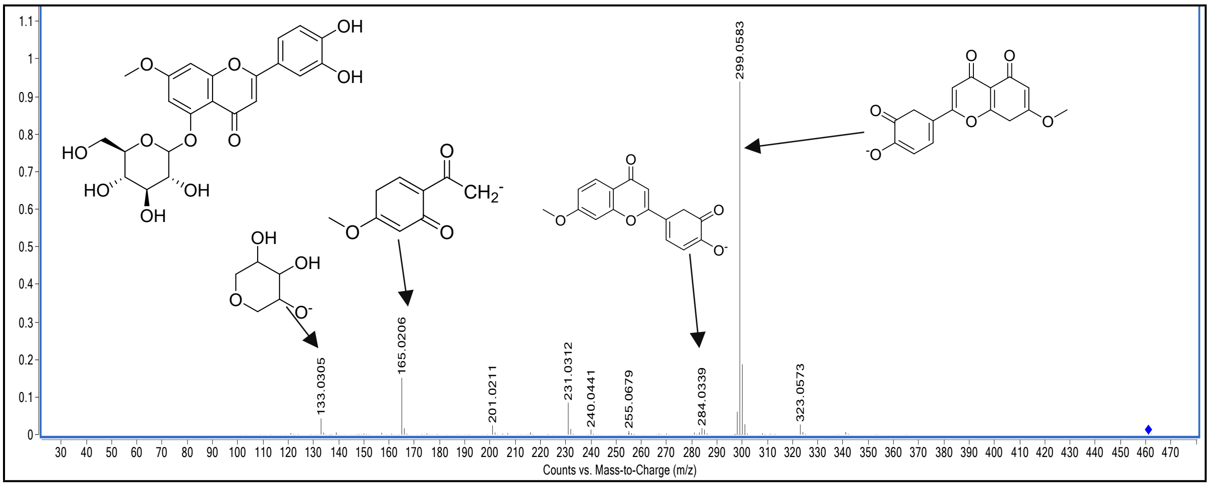

10. MS/MS Spectra of 3-p-coumaroyl quinic acid

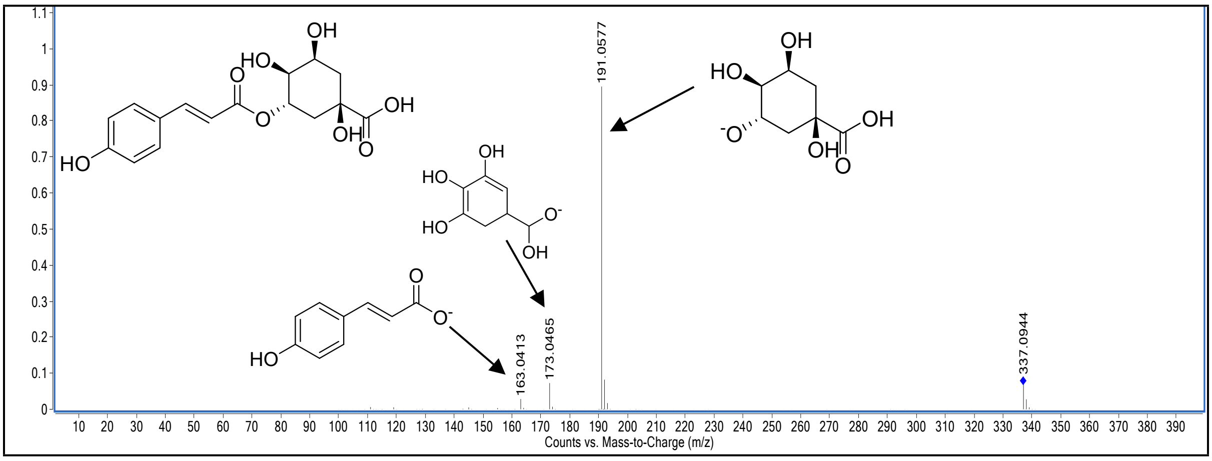

11. MS/MS Spectra of Scutellarein 7-methyl ether 6-galactoside

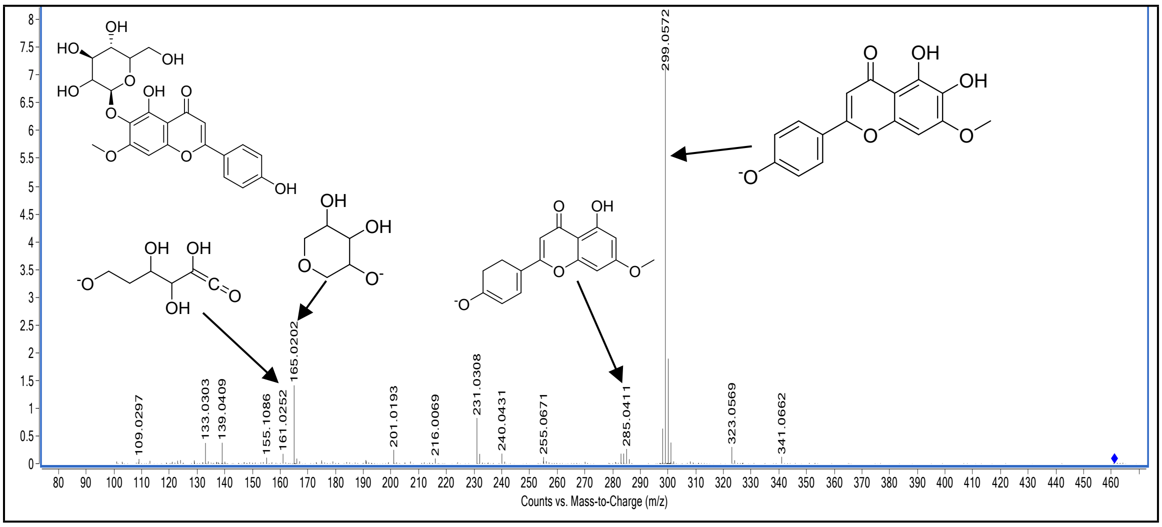

12. MS/MS Spectra of 2''-*O*-vanilloylvitexin

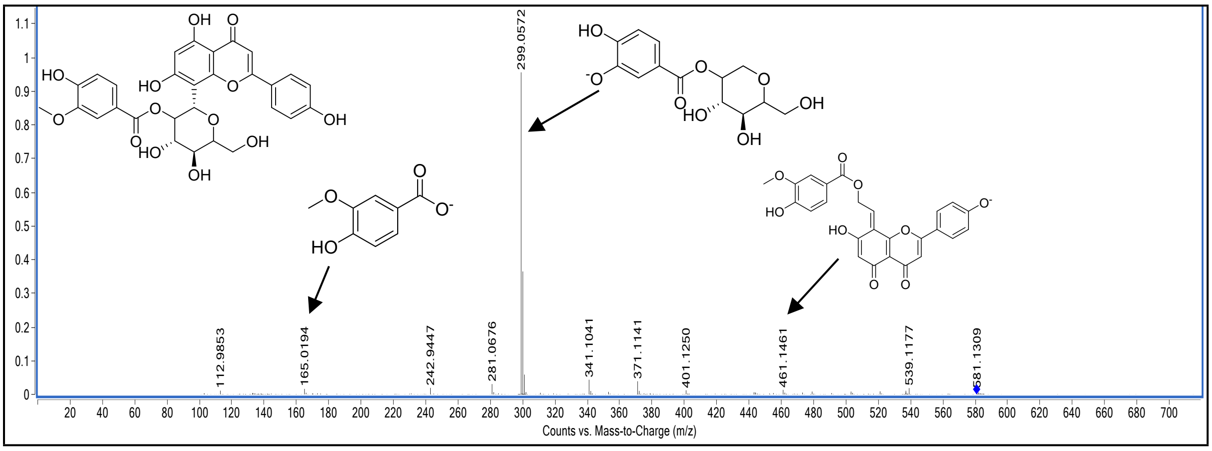

13. MS/MS Spectra of Chrysin 7-glucuronide

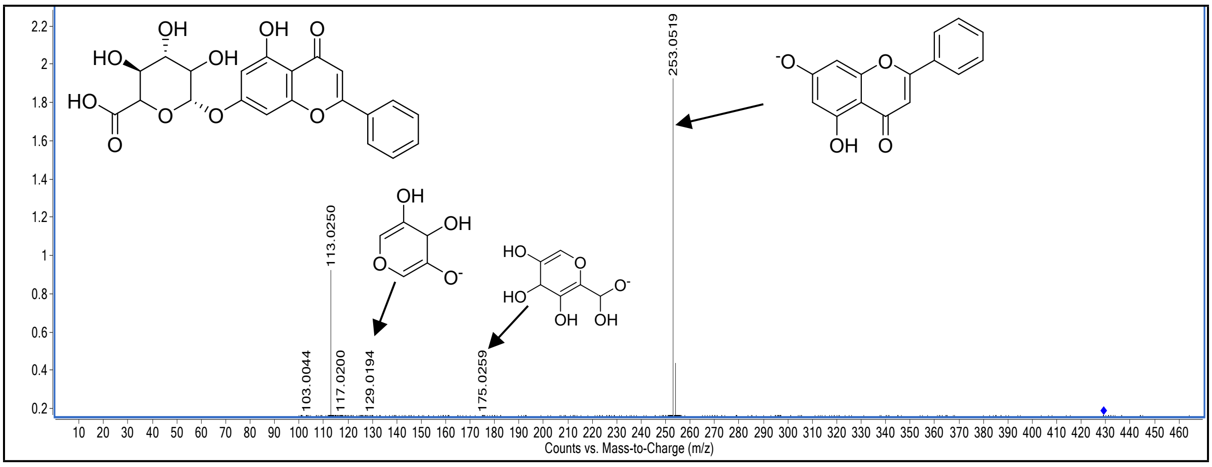

14. MS/MS Spectra of Peonidin 3-*O*-galactoside

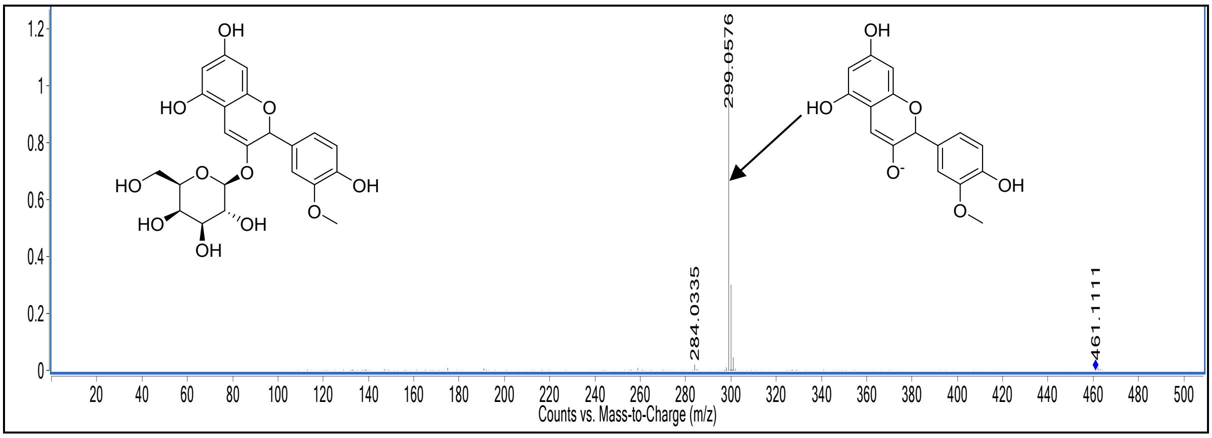

15. MS/MS Spectra of Echioidinin

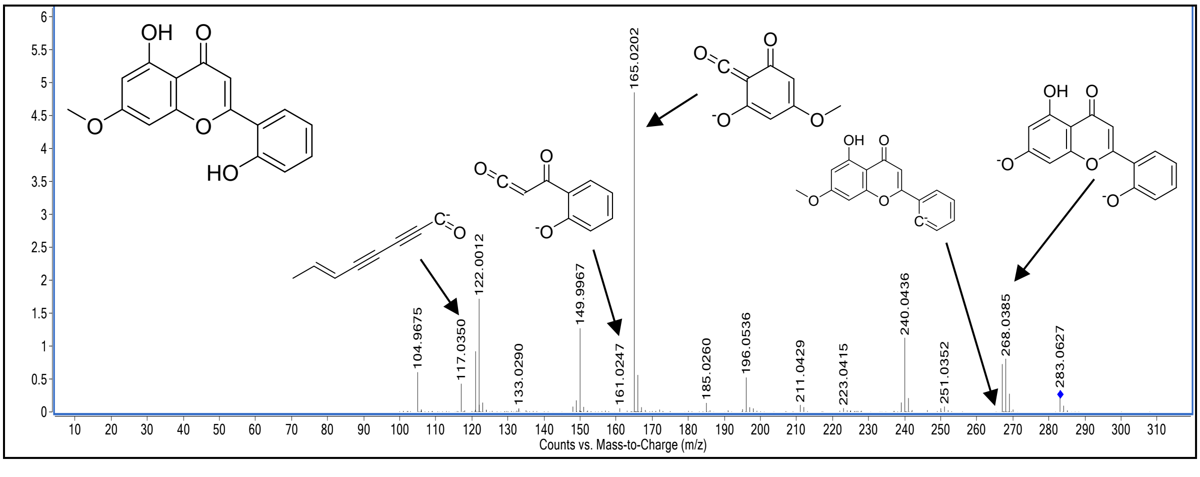

16. MS/MS Spectra of Oroxylin A

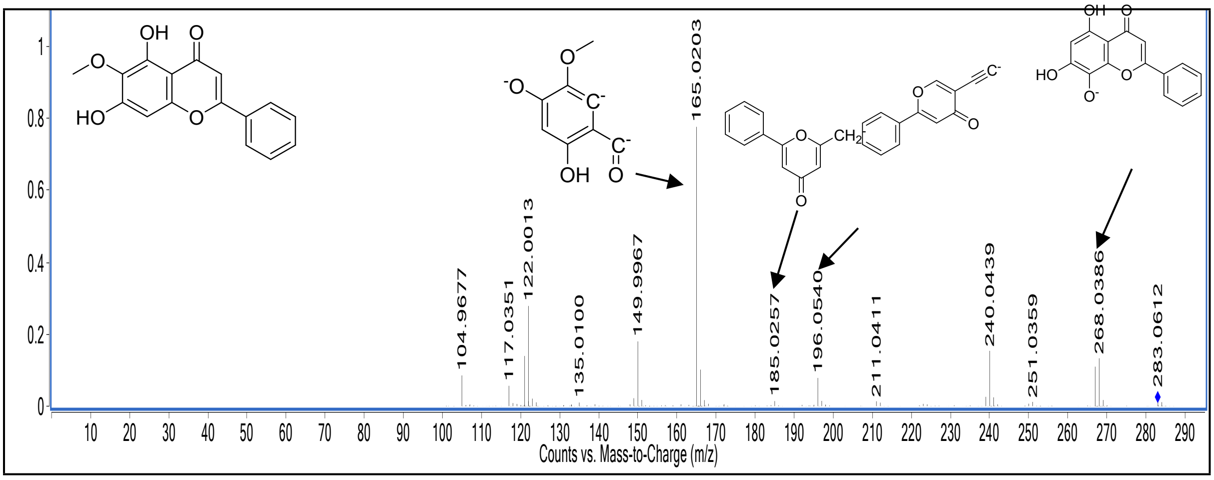

17. MS/MS Spectra of Wogonin

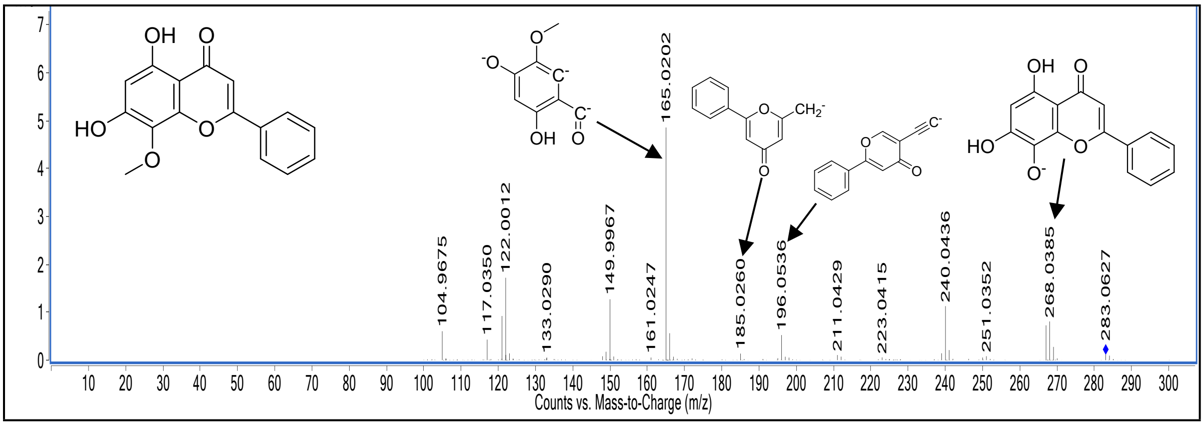

18. MS/MS Spectra of 3,5-Dihydroxy-7,8-dimethoxyflavone

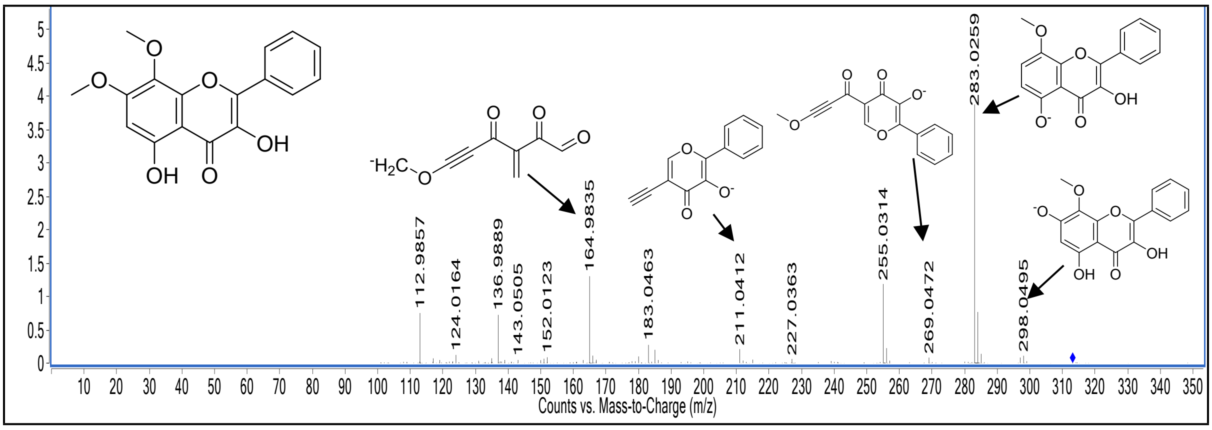

19. MS/MS Spectra of Skullcapflavone I

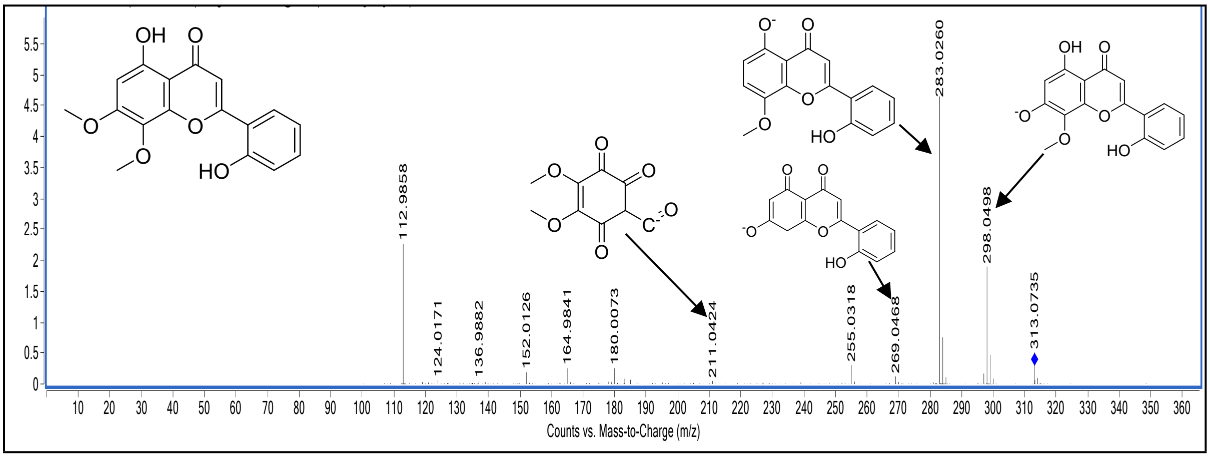

20. MS/MS Spectra of 7,2'-Dihydroxy-5-methoxyflavone

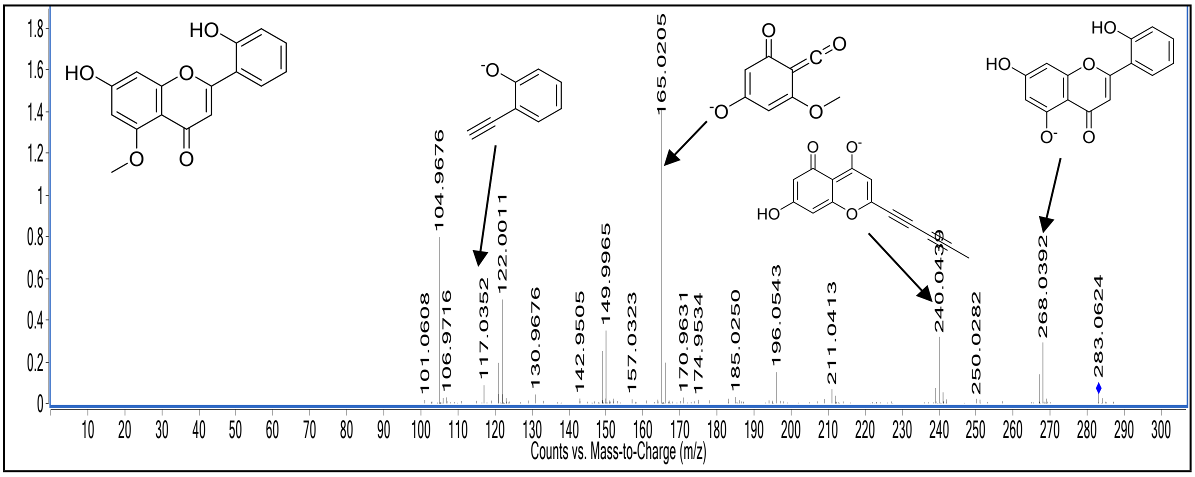

Figure S3. MS/MS fragmentation spectra of the identified compounds from the methanolic extract of *A. nallamalayana*.

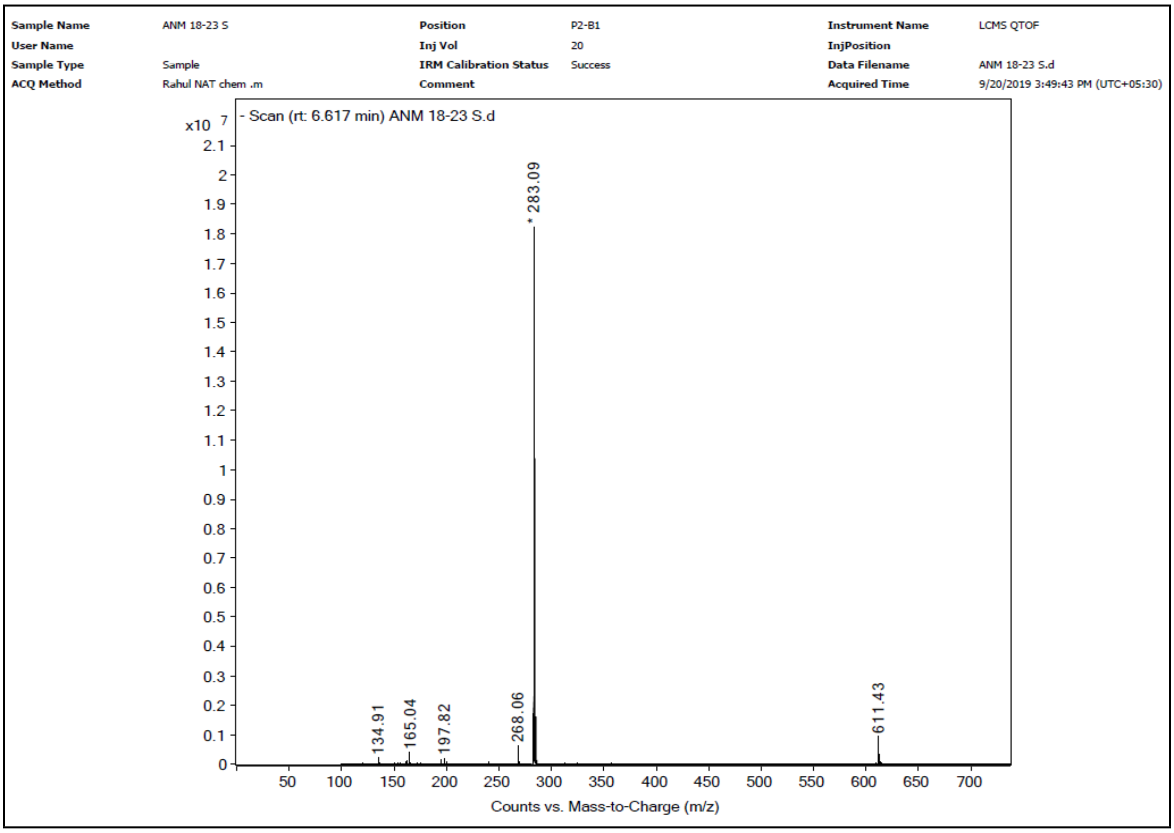

Figure S4. HRMS spectra of **Compound 1**.

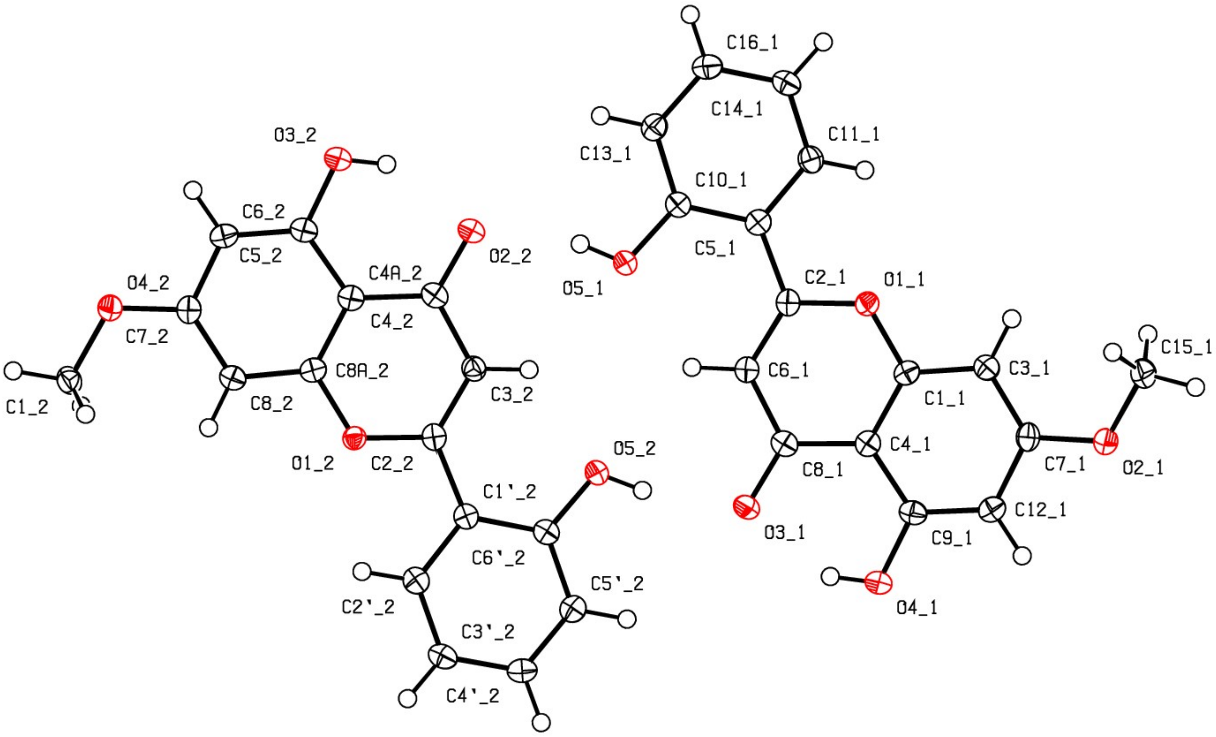

Figure S5. Thermal ellipsoid plot of **Compound 1 (CCDC No: 2072153)**. Ellipsoids are represented with 50% probability.

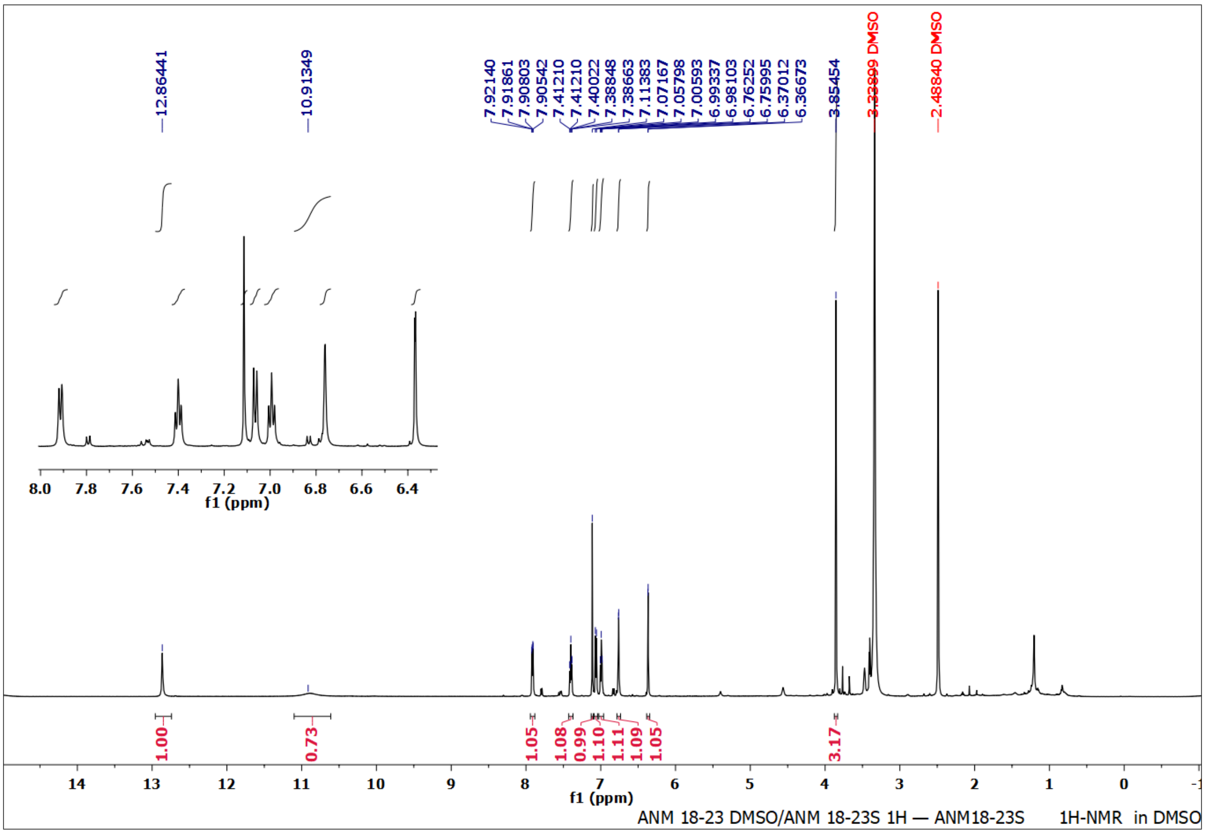

Figure S6. ^{1}H NMR of **Compound 1**.

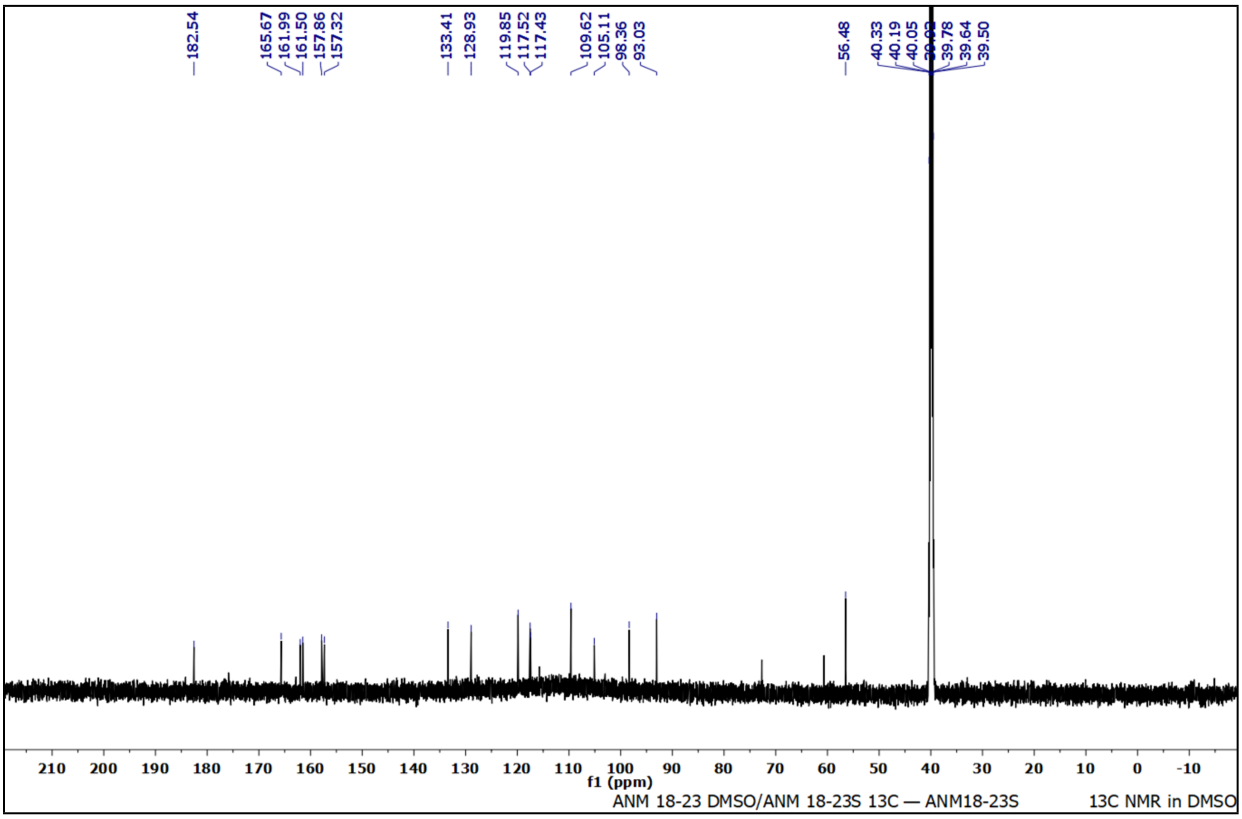

Figure S7. ^{13}C NMR of **Compound 1**.

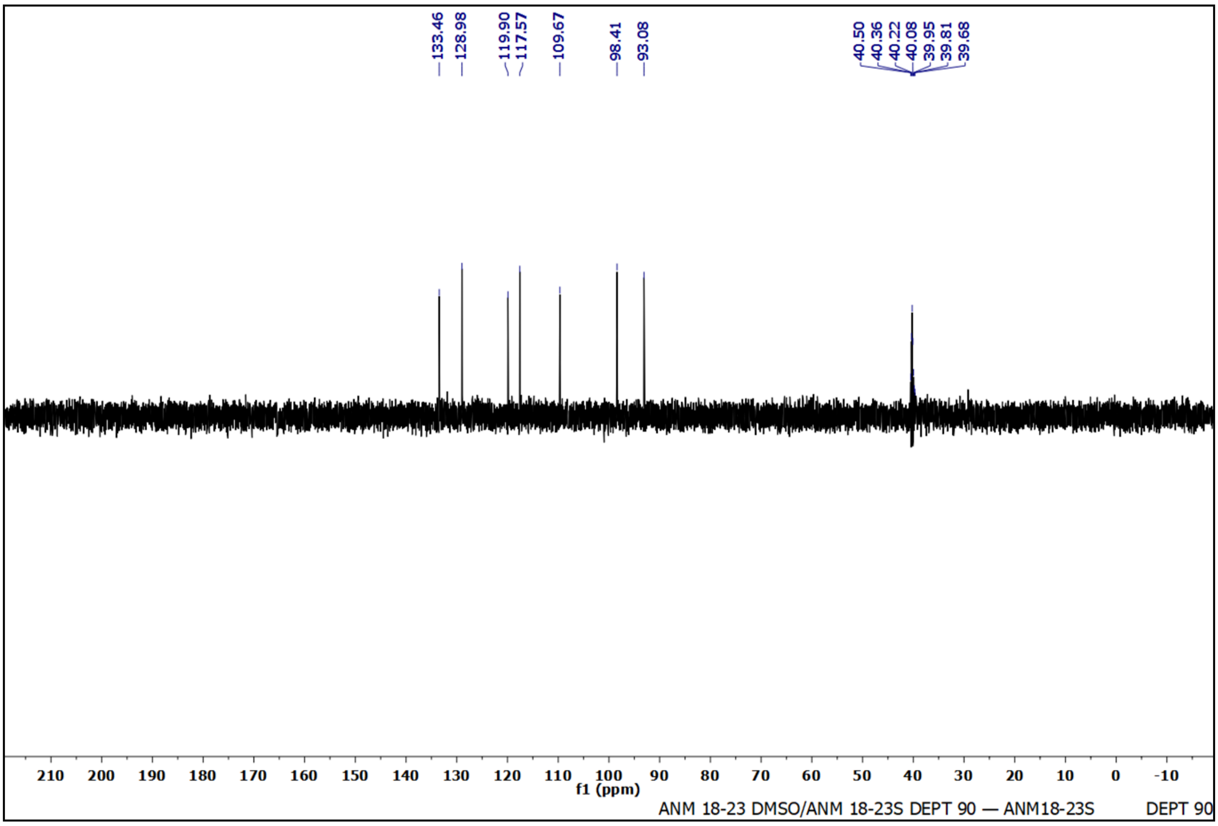

Figure S8. DEPT-90 NMR of **Compound 1.**

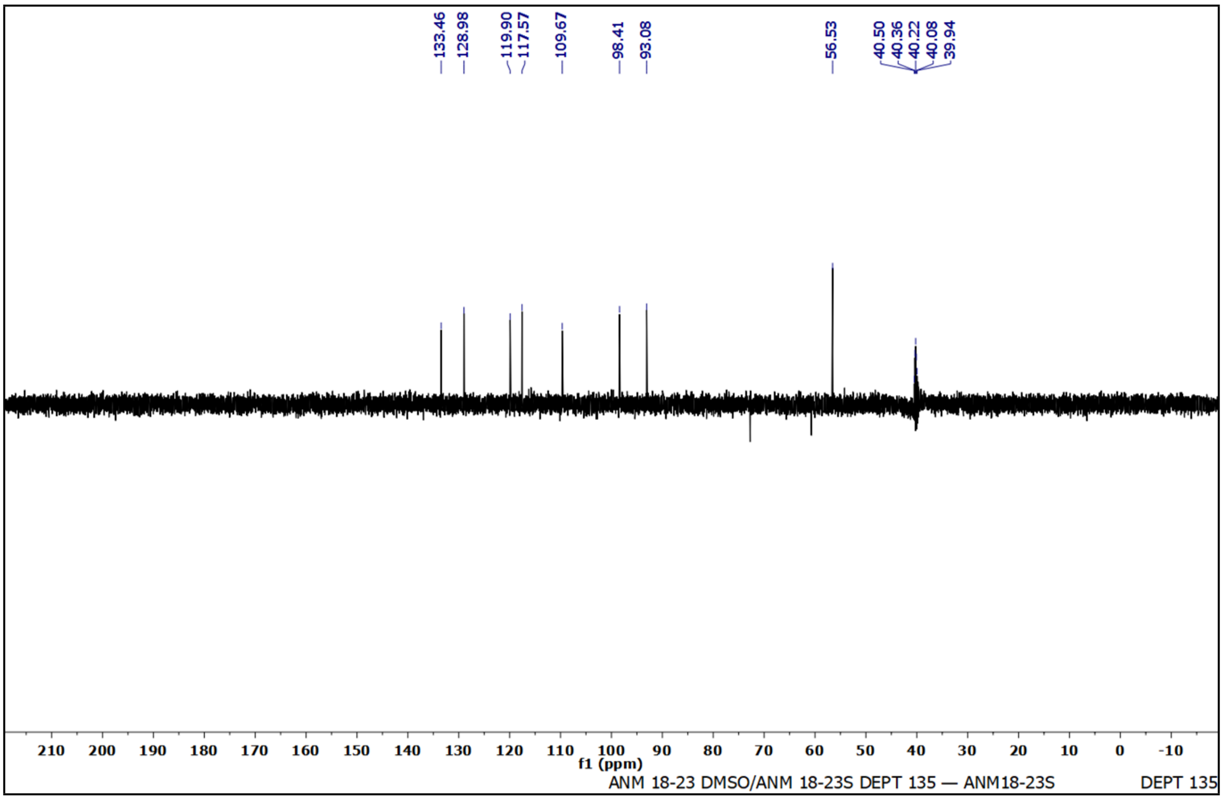

Figure S9. DEPT-135 NMR of **Compound 1.**

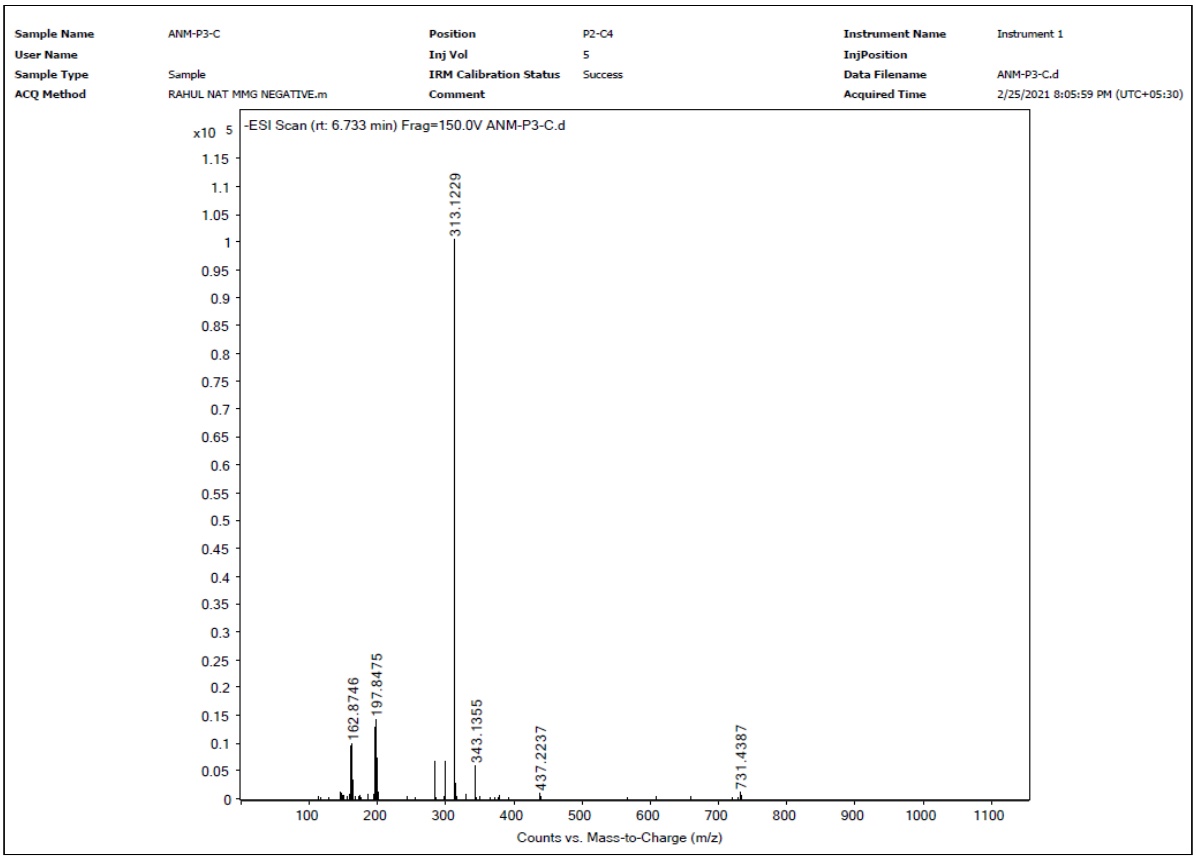

Figure S 10. HRMS Spectra of **Compound 2**.

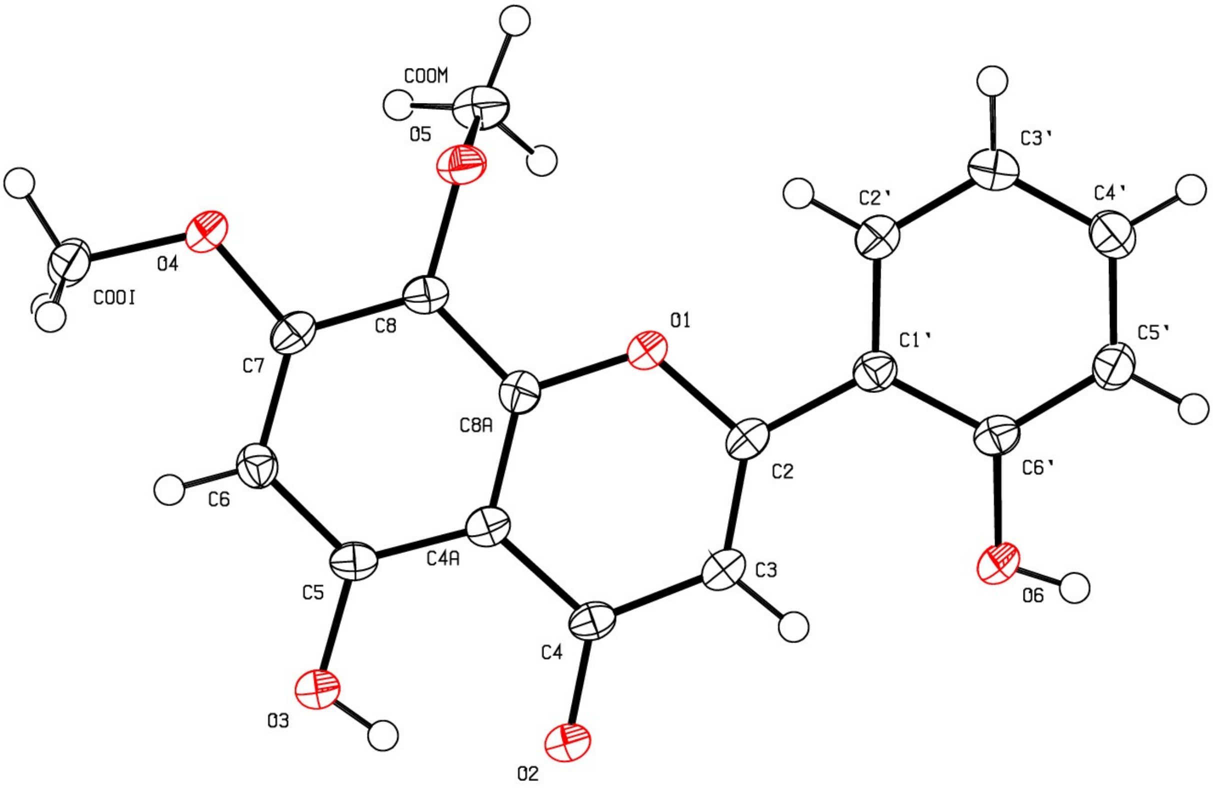

Figure S11. Thermal ellipsoid plot of **Compound 2 (CCDC No: 2072155)**. Ellipsoids are represented with 50% probability.

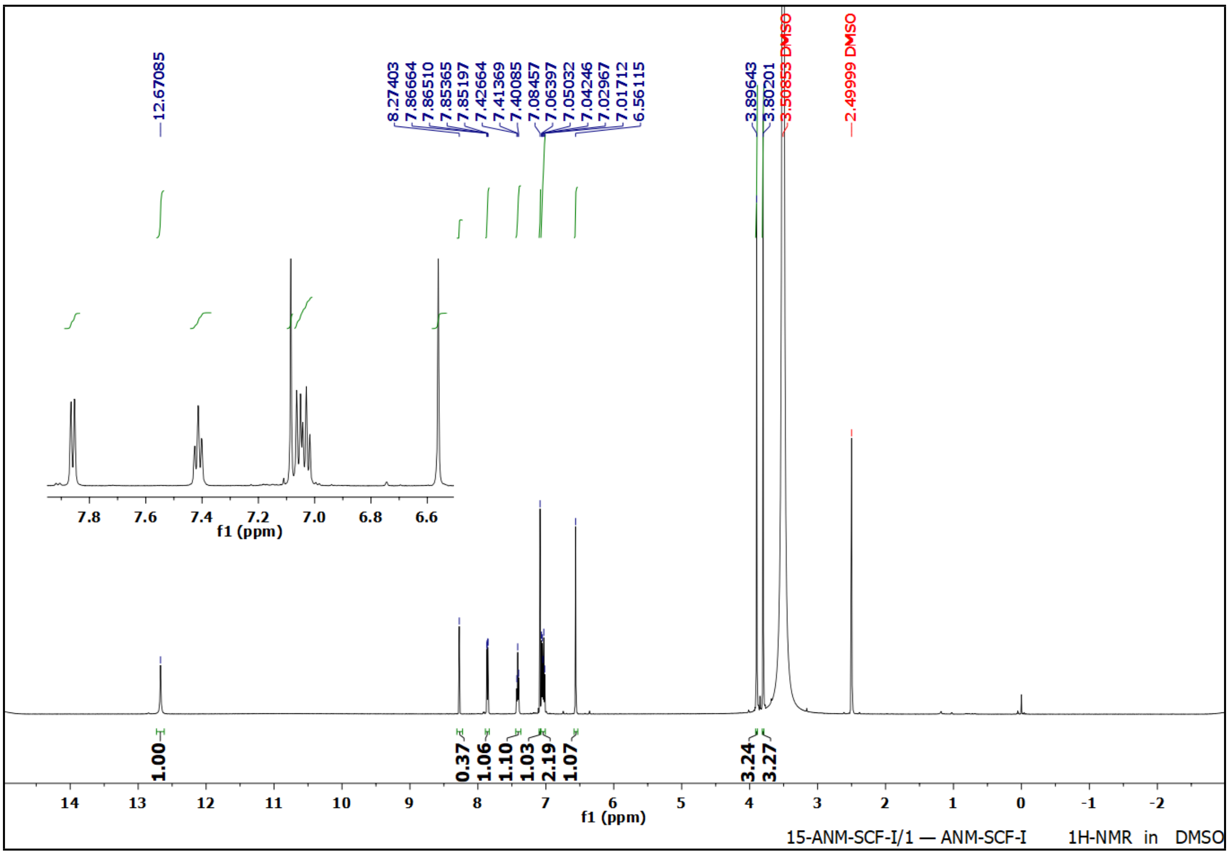

Figure S12. ^{1}H NMR of **Compound 2**.

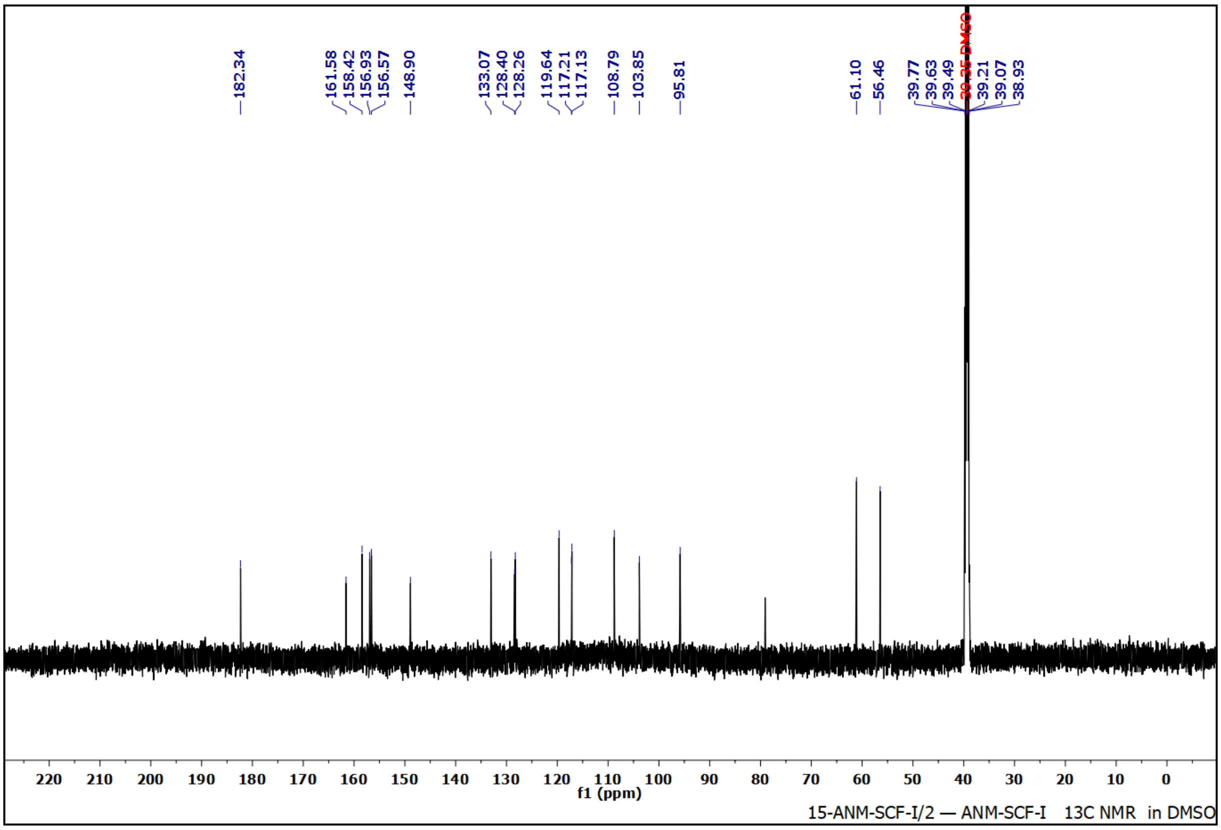

Figure S13. ^{13}C NMR of **Compound 2.**

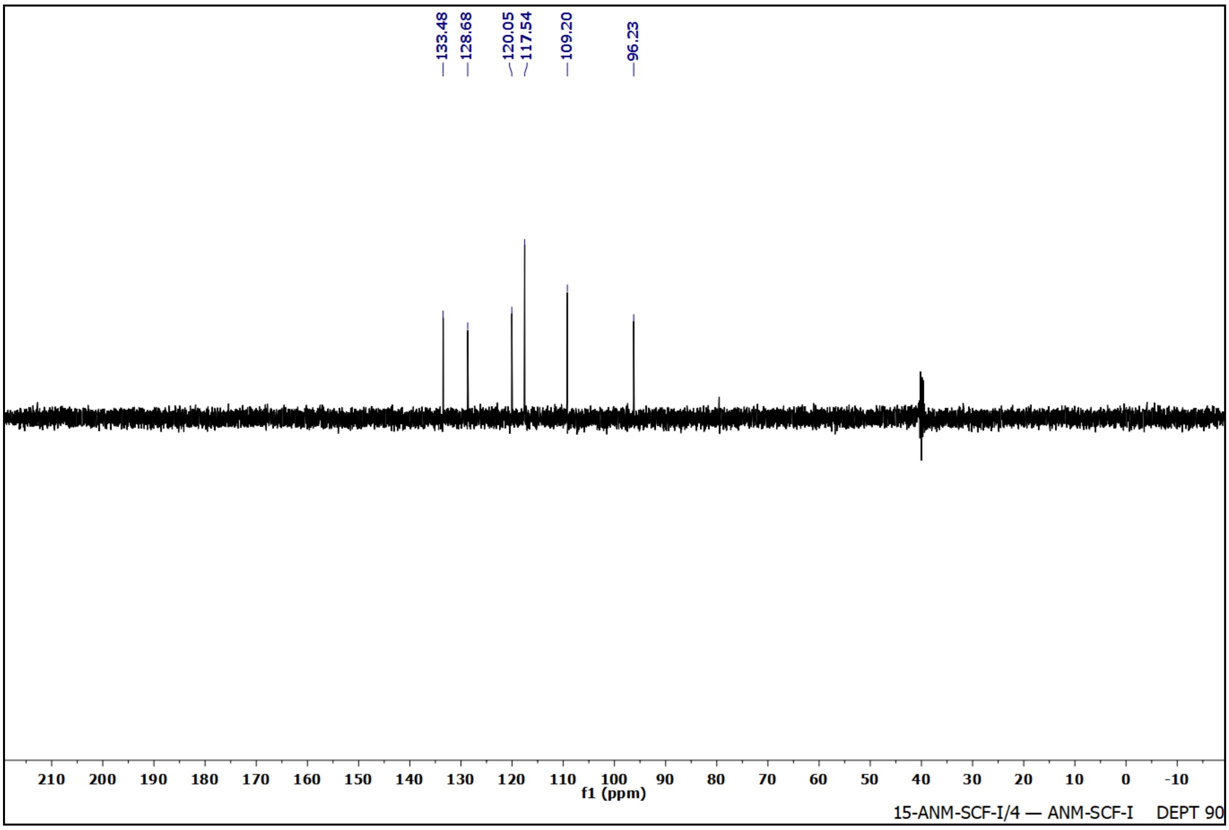

Figure S14. DEPT-90 NMR of **Compound 2.**

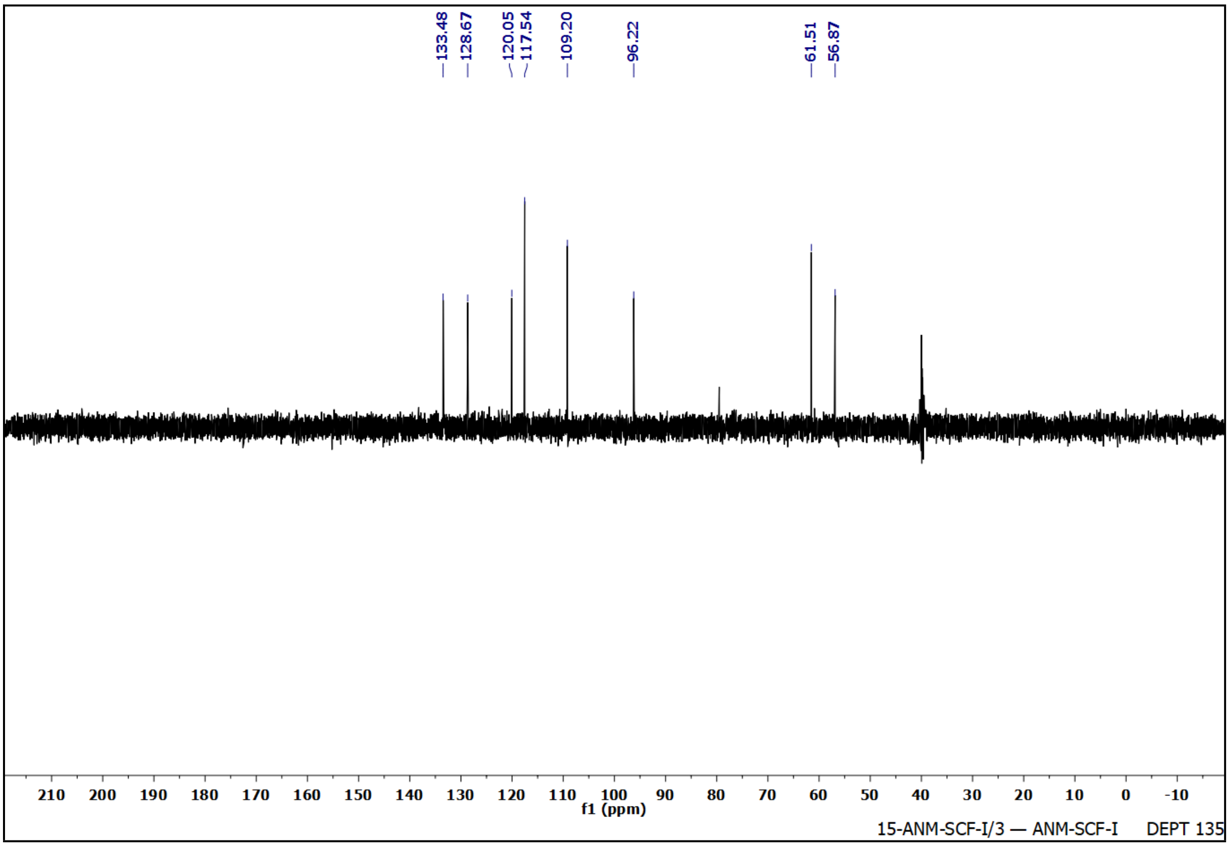

Figure S15. DEPT-135 NMR Spectra of **Compound 2**.

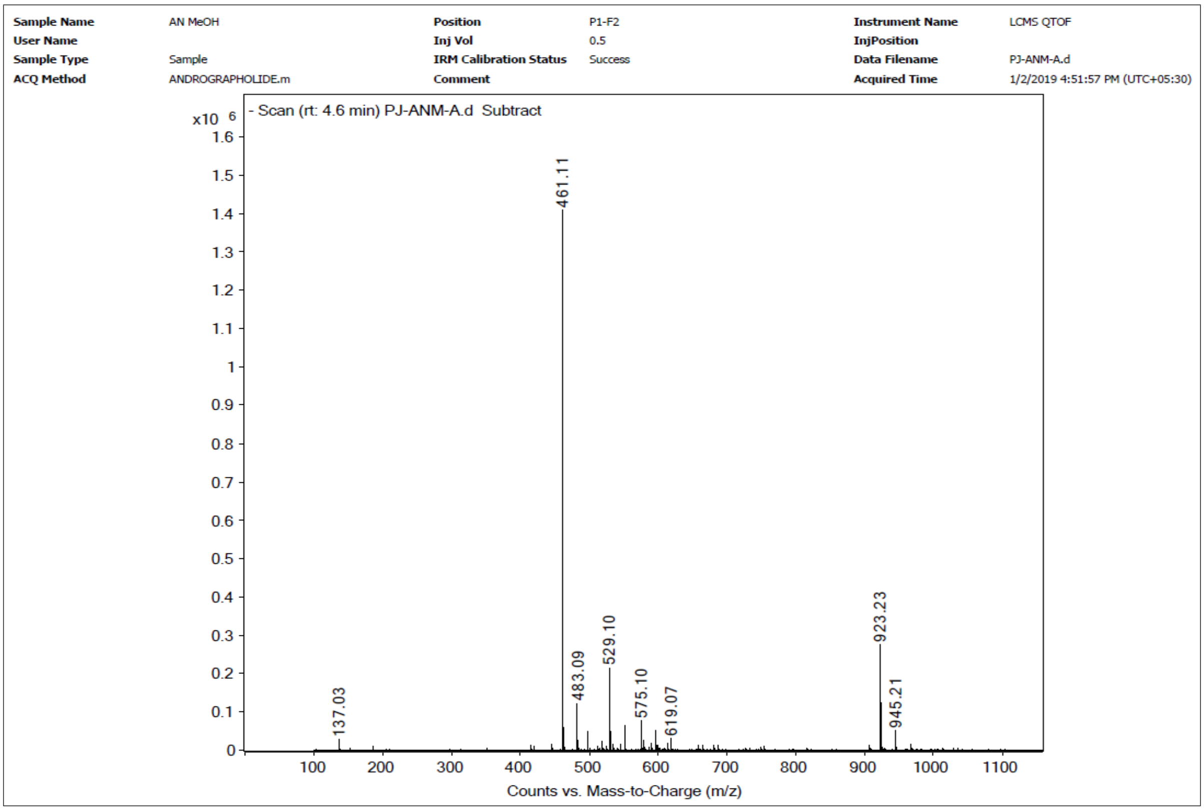

Figure S16. HRMS Spectra of **Compound 3**.

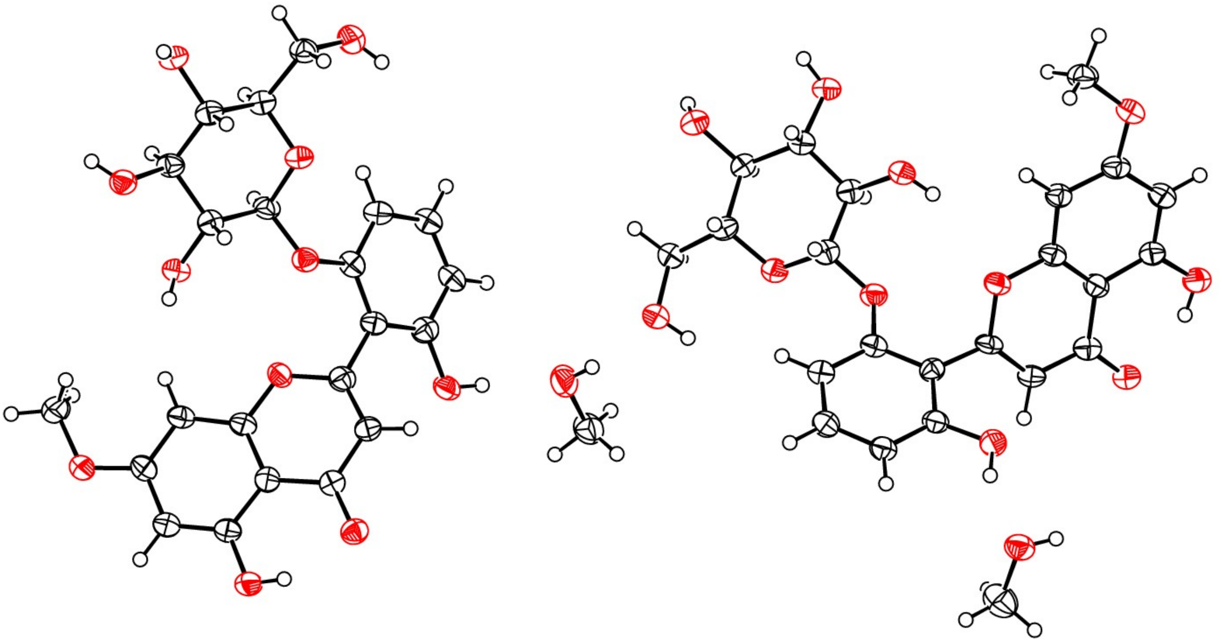

Figure S17. Thermal ellipsoid plot of **Compound 3 (CCDC No.: 2072714).** Ellipsoids are represented with 50% probability.

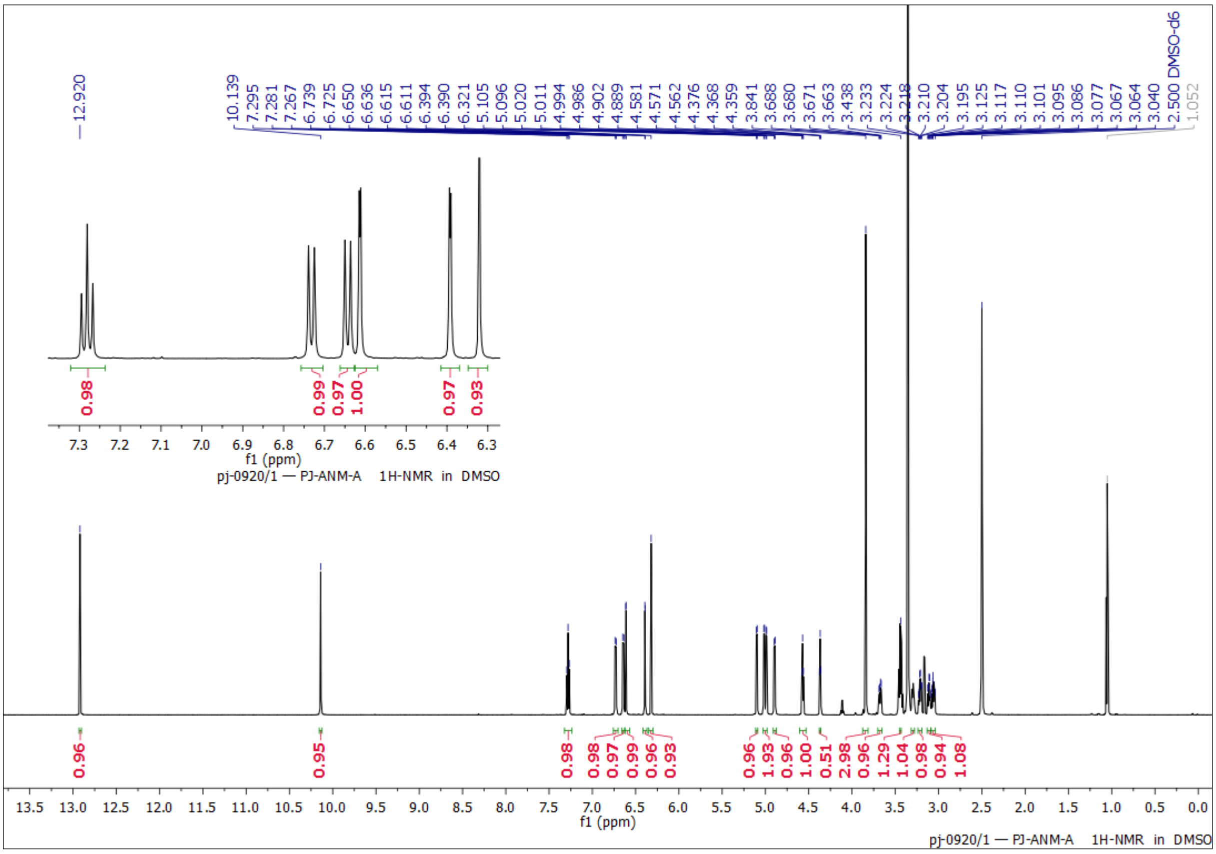

Figure S 18. ^{1}H NMR of **Compound 3.**

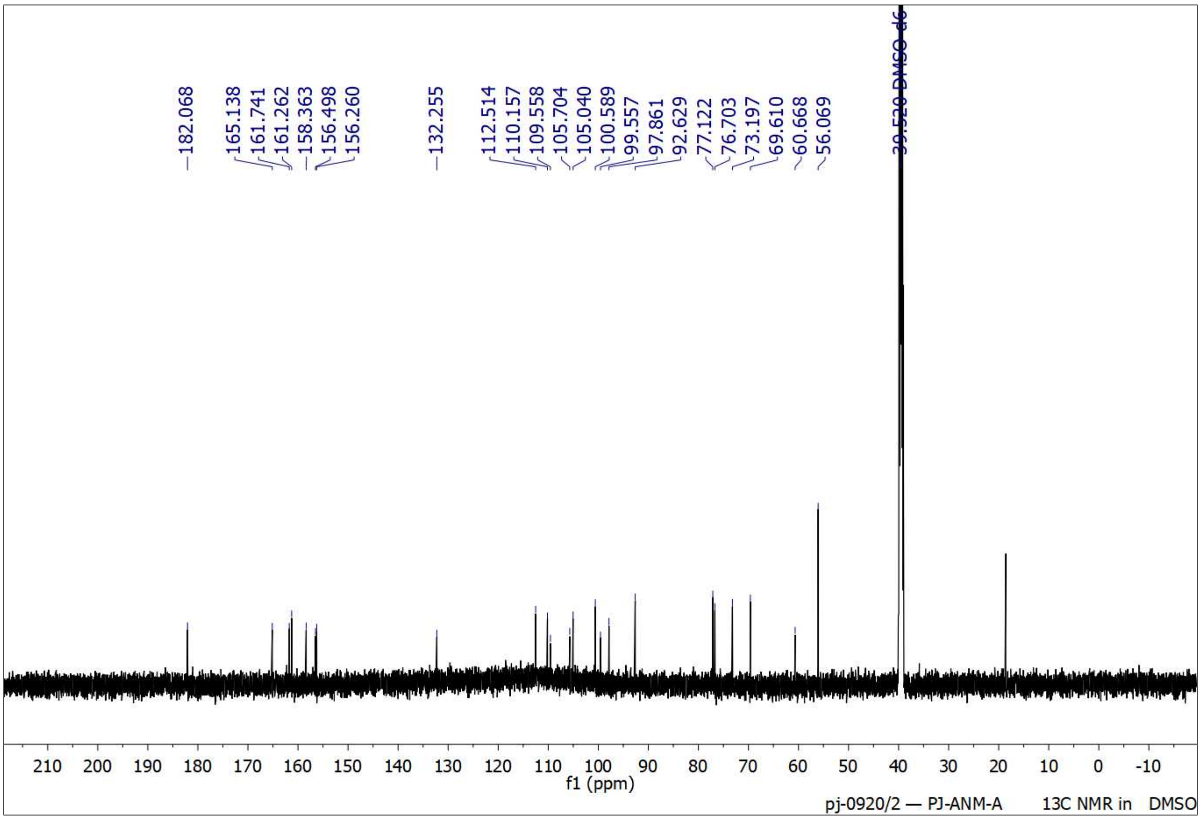

Figure S19. ^{13}C NMR of **Compound 3**.

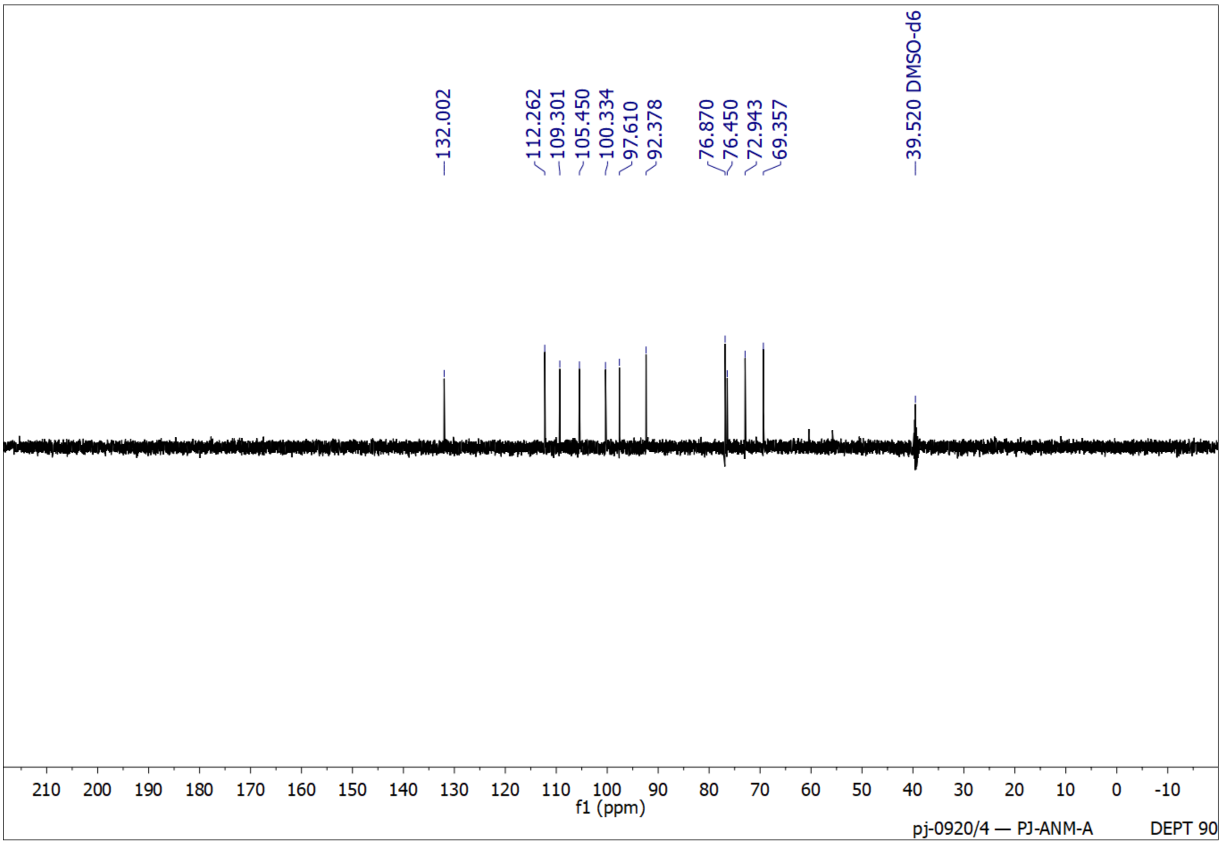

Figure S20. DEPT-90 NMR of **Compound 3**.

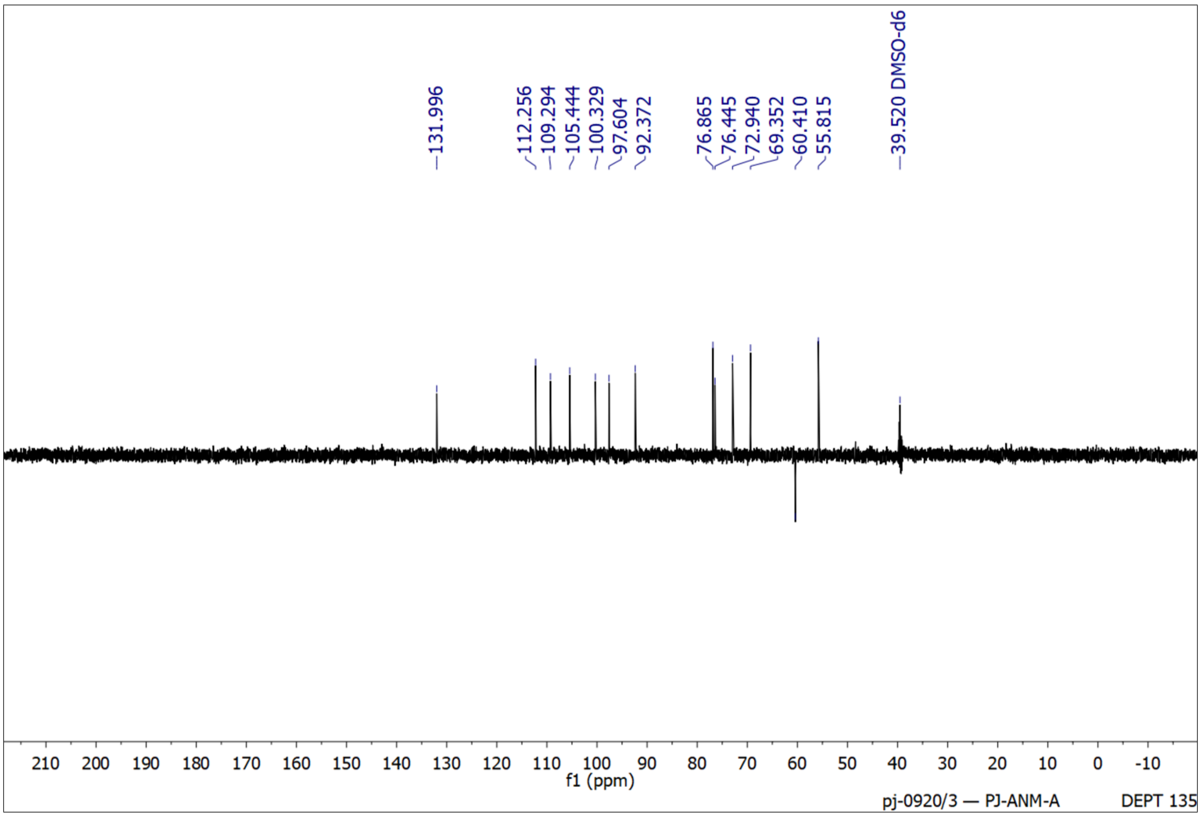

Figure S21. DEPT-135 NMR of **Compound 3**.

CheckCif alerts are listed according to Alert Level for Compound 3. Response is in bold.

Compound 3 (CCDC Deposition No.: 2072714)

Datablock: anm_28_ml_25_0m_a

Alert level B

Alert level B

PLAT417_ALERT_2_B Short Inter D-H..H-D H1 ..H5_2 . 2.06 Ang.

x,y,z = 1_555 Check

PLAT420_ALERT_2_B D-H Bond Without Acceptor O11_1 --H11_1 . Please Check

Author Response

We tried many times to get better data for this structure, but due to enantiomeric impurity, twinning, low crystal quality, the R_1 value is high. These alerts are all related to the hydrogen atoms of the solvent molecule and due to intermolecular hydrogen bonding within the compound. Since hydrogen atoms have low scattering power, it is challenging to locate them using X-ray data precisely. To add the hydrogen atoms, we used two methods: DIF-Fourier maps and HADD orders. The latest HADD instructions yield the best results. This structure is reported to support the molecule's conformation, which can be determined based on available data, and we are confident that the structural characterization is valid.

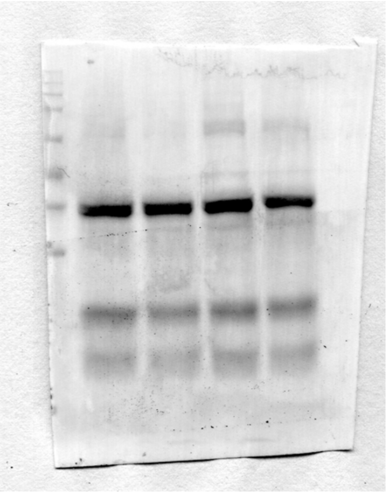

β-actin

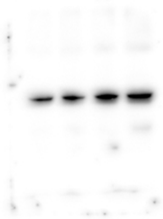

PUMA α

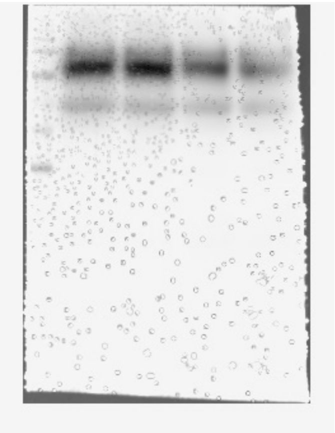

Bcl 2

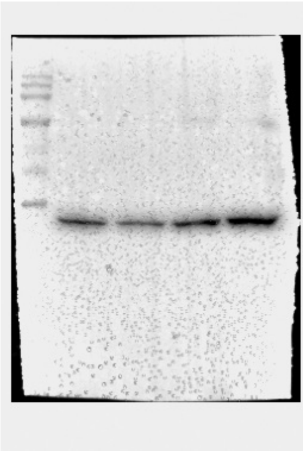

Cleaved Caspase 9

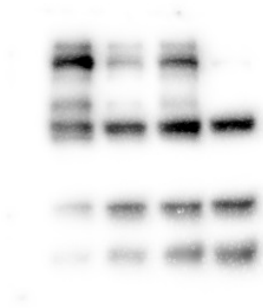

Cleaved Parp-1

Figure S22: Raw validation data and unprocessed original images of gels and western blots related to **Fig. 4**
